# Site Specific Preparation of *N‐*Glycosylated Peptides: Thioamide‐Directed Activation of Aspartate

**DOI:** 10.1002/anie.202210367

**Published:** 2022-09-21

**Authors:** Ameer B. Taresh, Craig A. Hutton

**Affiliations:** ^1^ School of Chemistry and Bio21 Molecular Science and Biotechnology Institute The University of Melbourne Melbourne Victoria 3010 Australia

**Keywords:** Glycopeptides, Glycosylation, Site-Specific, Thioamide

## Abstract

A site‐specific method for the preparation of *N*‐glycosylated peptides is described. Incorporation of a peptide backbone thioamide linkage adjacent to an Asp residue facilitates a Ag^I^‐promoted, site‐specific conversion to *N‐*glycosylated Asn residues in peptides.

## Graphical abstract

A single‐atom (O→S) substitution to introduce a backbone thioamide linkage in a peptide facilitates a Ag^I^‐promoted, site‐specific generation of Asn *N‐*glycopeptides.

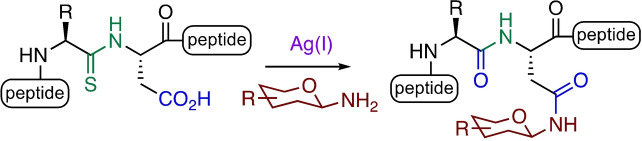

Glycoproteins are an important class of biomolecule that control a variety of important biological functions including immune and inflammatory responses, cell adhesion, signalling and protein folding.[^1^, ^2^, ^3^, ^4^] Erroneous glycosylation can lead to auto‐immune disease, cancer, and other disorders. Further, *N‐*glycopeptides are useful as biomarkers of disease.^[5]^

A variety of methods have been developed for the assembly of *N‐*glycopeptides and glycoproteins (Figure 1). The linear approach to *N‐*glycopeptides involves the synthesis of specific *N‐*glycosyl‐asparagine building blocks through the coupling of an aminoglycan precursor to an aspartic acid derivative, followed by incorporation into solid‐phase peptide synthesis (SPPS) to generate glycopeptides.[^6^, ^7^, ^8^] Disadvantages of this method include that an excess of the glyco‐Asn building block is required, and that some glycosidic bonds in complex oligosaccharides are prone to decomposition under the acidic conditions used in SPPS and cleavage from the resin.^[9]^

**Figure 1:**
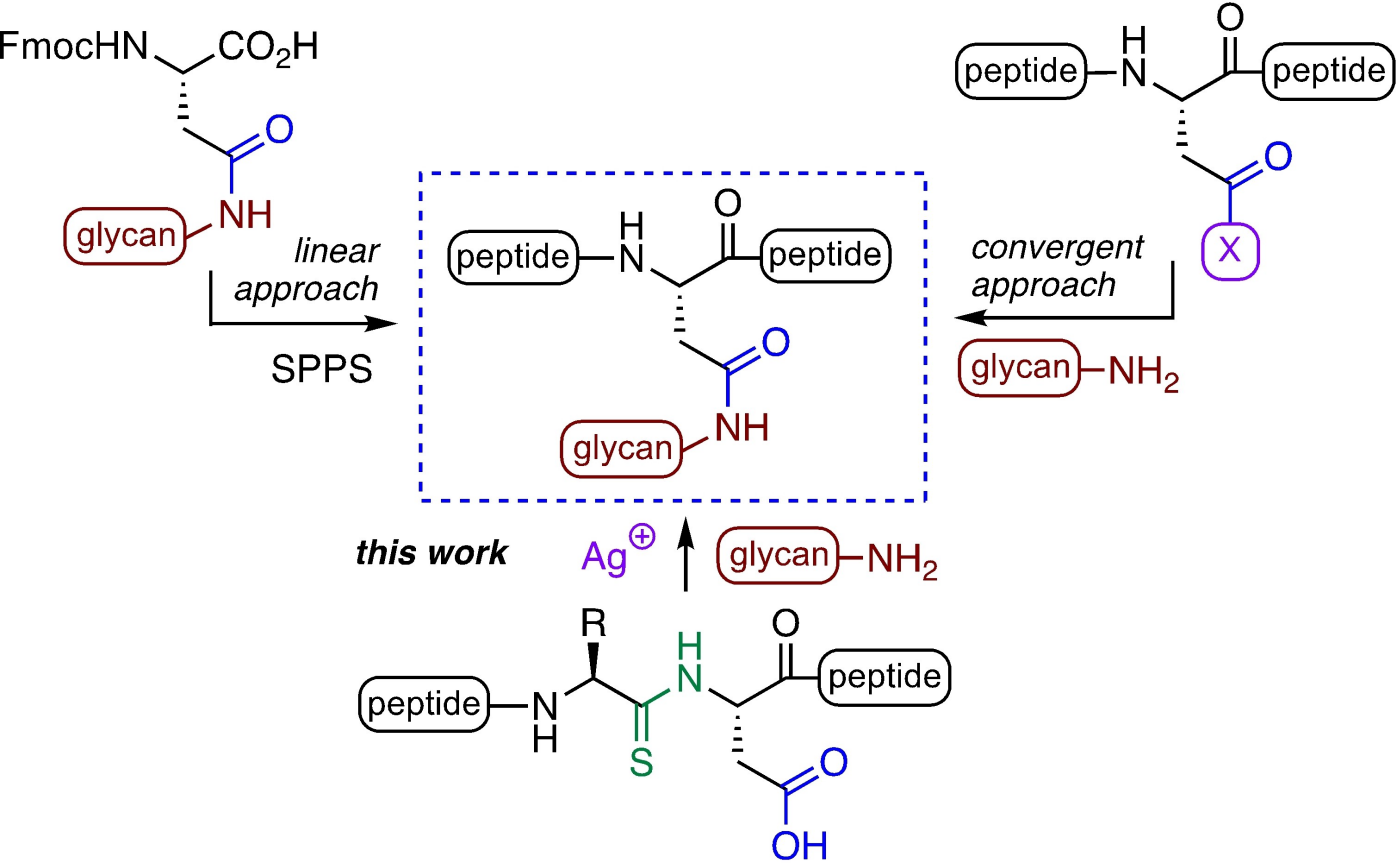
Approaches to *N‐*glycopeptides.

The convergent approach to glycopeptides involves the coupling of an aminoglycan to the aspartate side chain on a partially protected peptide to produce a full‐length glycopeptide.[^10^, ^11^] Although the convergent method has been applied to the synthesis of complex glycopeptides,[^12^, ^13^] it requires orthogonal protection of the targeted aspartate residue. In addition, activation of this aspartate side chain carboxyl group is commonly plagued by aspartimide formation.^[14]^ Methods to provide chemoselectivity include the use of aspartate thioacid,^[15]^ thioester^[16]^ or selenoester‐containing^[17]^ peptides. Selective amination of the modified aspartate provides the *N‐*glycopeptides, though orthogonal protection is still required for site‐selective activation of the aspartate, and aspartimide formation remains an issue.

We have recently developed new methods for the Ag^I^‐promoted chemoselective reaction of peptide thioamides.[^18^, ^19^] We have exploited this approach in the macrocyclization of peptides containing a single O→S atom substitution, and in the ring expansion of cyclic peptides.^[20]^ In these processes, Ag^I^‐promoted combination of the thioamide and carboxylate moieties generates isoimide and/or imide intermediates, that are trapped by amine nucleophiles to generate new amide bonds (Scheme 1).[^15^, ^21^, ^22^, ^23^, ^24^]

**Scheme 1:**
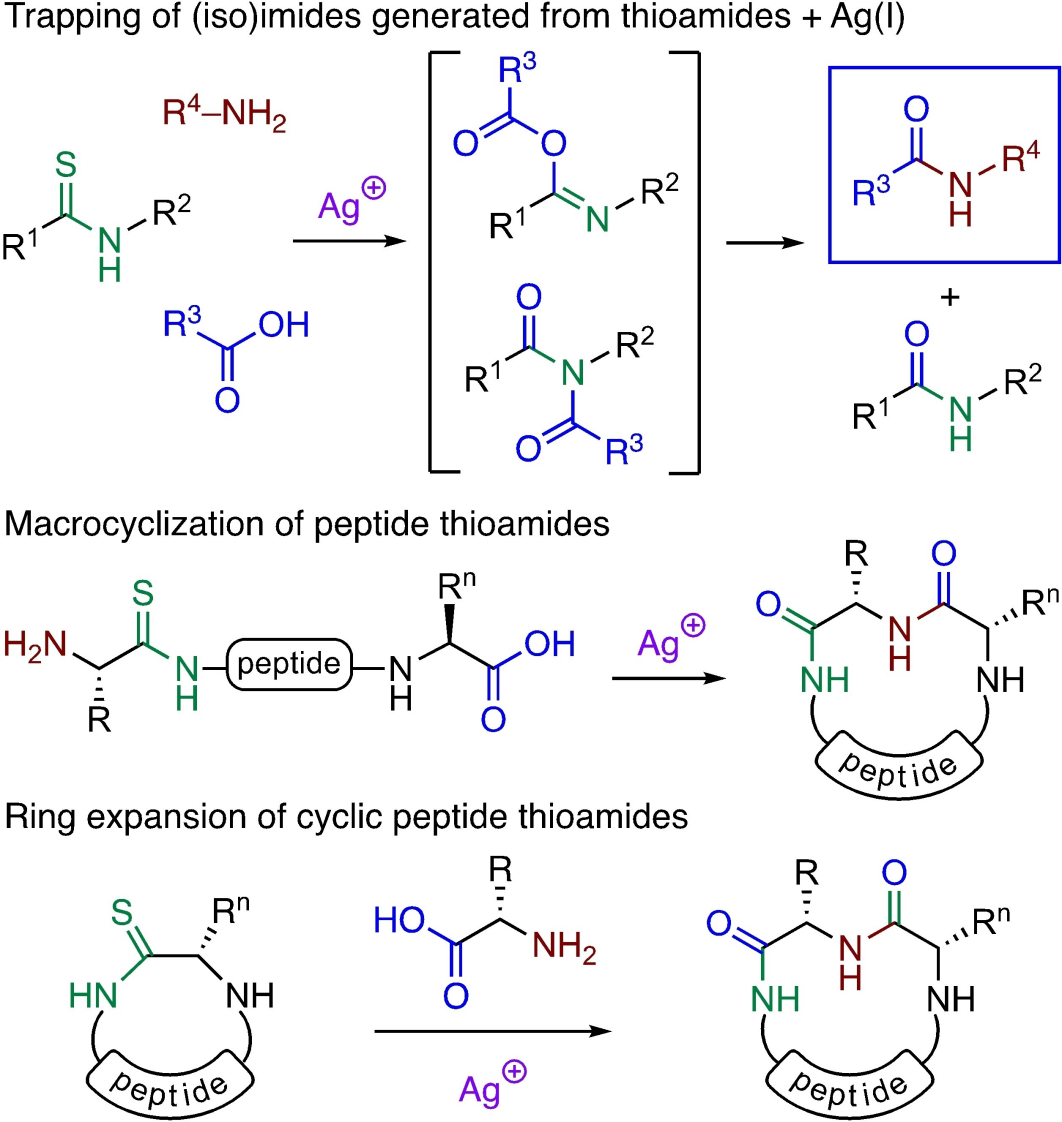
Ag^I^‐promoted reactions of peptide thioamides.

Herein we describe a site‐specific method for the formation of *N*‐glycosylated peptides (Scheme 2). This new method employs an activating group positioned on the backbone, rather than on the Asp side chain. Thioamide‐triggered chemoselective glycosylation of aspartic acid residues generates topographically‐defined *N*‐glycosylated asparagine derivatives and overcomes drawbacks of existing methods such as site selectivity and aspartimidation.

**Scheme 2:**
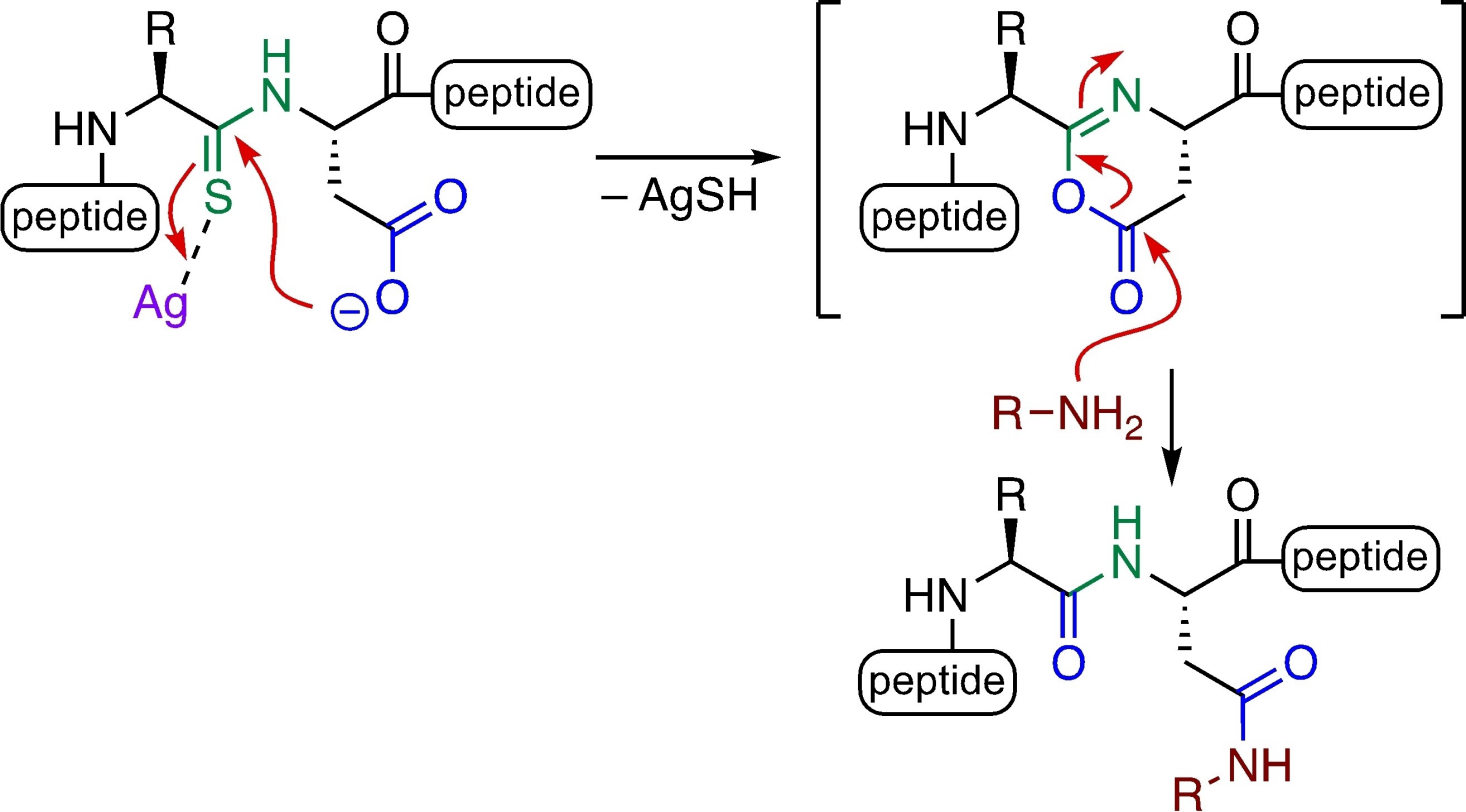
This work: Ag^I^‐promoted conversion of Asp‐thioamide to *N‐*glycosyl Asn.

Our previous studies of the reactions of thioamides with carboxylic acids demonstrated the formation of isoimide intermediates, which can undergo rearrangement to imides or acyl transfer to amines to generate amides. We envisaged that intramolecular reaction of an aspartate residue containing a backbone thioamide on the *N‐*terminal side should generate a cyclic isoimide intermediate (Scheme 2). 1,3‐Acyl transfer to generate the corresponding imide in this system is not possible due to stereoelectronic effects.[^20^, ^25^] We have employed such isoimide intermediates in the generation of lactam‐stapled peptides through intramolecular trapping with lysine side chains.^[26]^ In this work, we demonstrate the intermolecular trapping of such isoimides with aminosugars to generate *N‐*substituted Asn derivatives (Scheme 2).

In order to investigate the proposed transformation, dipeptide thioamide **1** (see Supporting Information) was treated with 2.0 equiv of Ag_2_CO_3_ and 2.0 equiv of cyclohexylamine, to generate the asparagine derivative **2** in 54 % isolated yield (Scheme 3).

**Scheme 3:**
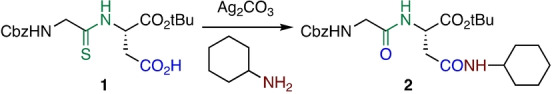
Model reaction: generation of *N‐*Cy Asn residue.

Following successful generation of the model *N‐*cyclohexyl asparagine derivative **2**, we turned to application of the Ag^I^‐promoted coupling to the generation of an *N‐*glycosylated asparagine motif. Accordingly, thioamide **3** was treated with varying amounts of silver carbonate and glucosylamine **4**, generating the *N‐*glucosylated asparagine **5** derivative in 32–78 % yields (Scheme 3 and Table 1). When using 2 equiv of aminosugar **4**, a slight excess of Ag_2_CO_3_ was found to be optimum, generating the amide **5** in 60 % yield (entry 5). Greater amounts of Ag^I^ resulted in a gradual decrease in the yield of the asparagine derivative. When using the optimized 1.2 equiv of Ag_2_CO_3_, a moderate excess (4 equiv) of aminosugar **4** gave optimum yields of **5** (entries 5–8). Use of different solvents did not alter the yields markedly, with CH_2_Cl_2_, CH_3_CN or mixtures thereof all providing the asparagine in 73–78 % yield (entries 7, 9, 10). In all cases complete consumption of the thioamide was observed, and the dipeptide (oxo)amide was observed as a minor byproduct.

**Table 1:** Optimisation of Ag^I^‐promoted conversion of Asp‐thioamide to *N‐*glycosyl Asn.

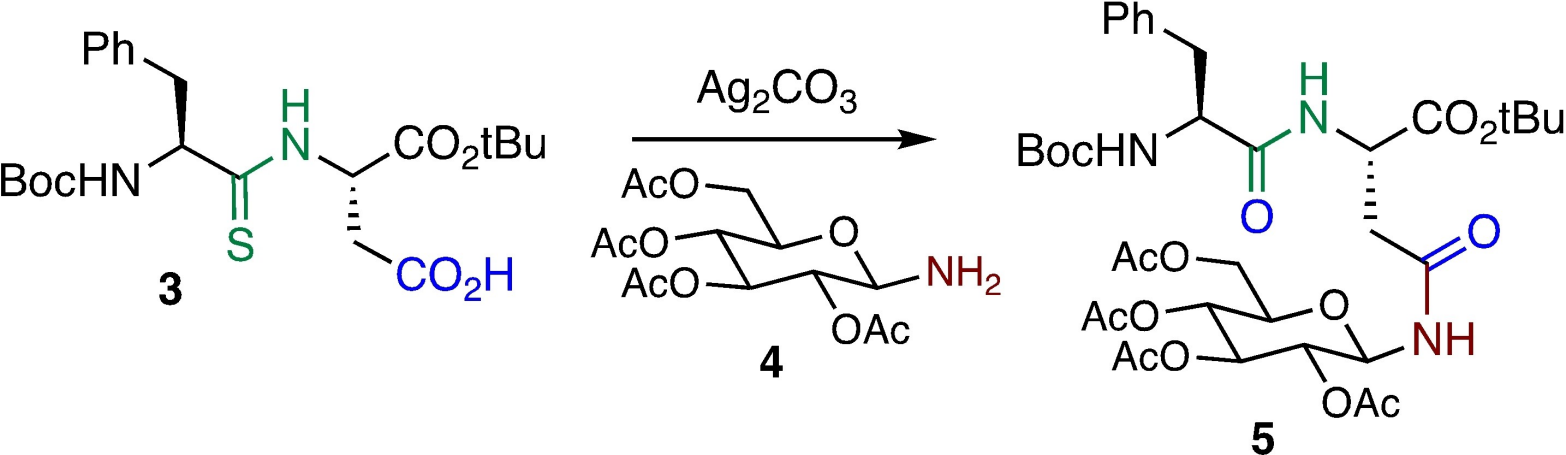				
Entry	Equiv. of Ag_2_CO_3_	Equiv. of **4**	Solvent	Yield of **5** [%]
1	1	2	CH_2_Cl_2_	40
2	2	2	CH_2_Cl_2_	51
3	3	2	CH_2_Cl_2_	32
4	1.5	2	CH_2_Cl_2_	54
5	1.2	2	CH_2_Cl_2_	60
6	1.2	3	CH_2_Cl_2_	68
7	1.2	4	CH_2_Cl_2_	74
8	1.2	5	CH_2_Cl_2_	70
9	1.2	4	CH_3_CN	73
10	1.2	4	CH_**2**_Cl_**2**_:CH_**3**_CN	78

With optimized conditions developed, the scope of the reaction with extended peptides was investigated. During biosynthesis, *N*‐glycosylation of proteins occurs at asparagine residues embedded in the consensus sequence N‐X‐S/T, where X can be any amino acid except proline. Thioamide‐containing peptides AZ^[S]^DAS **10** were therefore prepared, containing alanine at the X position and a variety of residues (Z) to the *N‐*terminal side of the aspartate, linked through a thioamide bond (Z=A, L, F, V, K, R, Scheme 4). Protected peptides **6** were prepared on Sieber amide resin, then incorporation of the thioamide linkage into the peptides was achieved through use of Fmoc aminoacyl benzotriazolides **7**.[^27^, ^28^, ^29^, ^30^, ^31^, ^32^] Following coupling with Ac‐Ala, peptide thioamides **10** were cleaved from the resin using 2 % TFA in DCM and purified by RP‐HPLC.

**Scheme 4:**
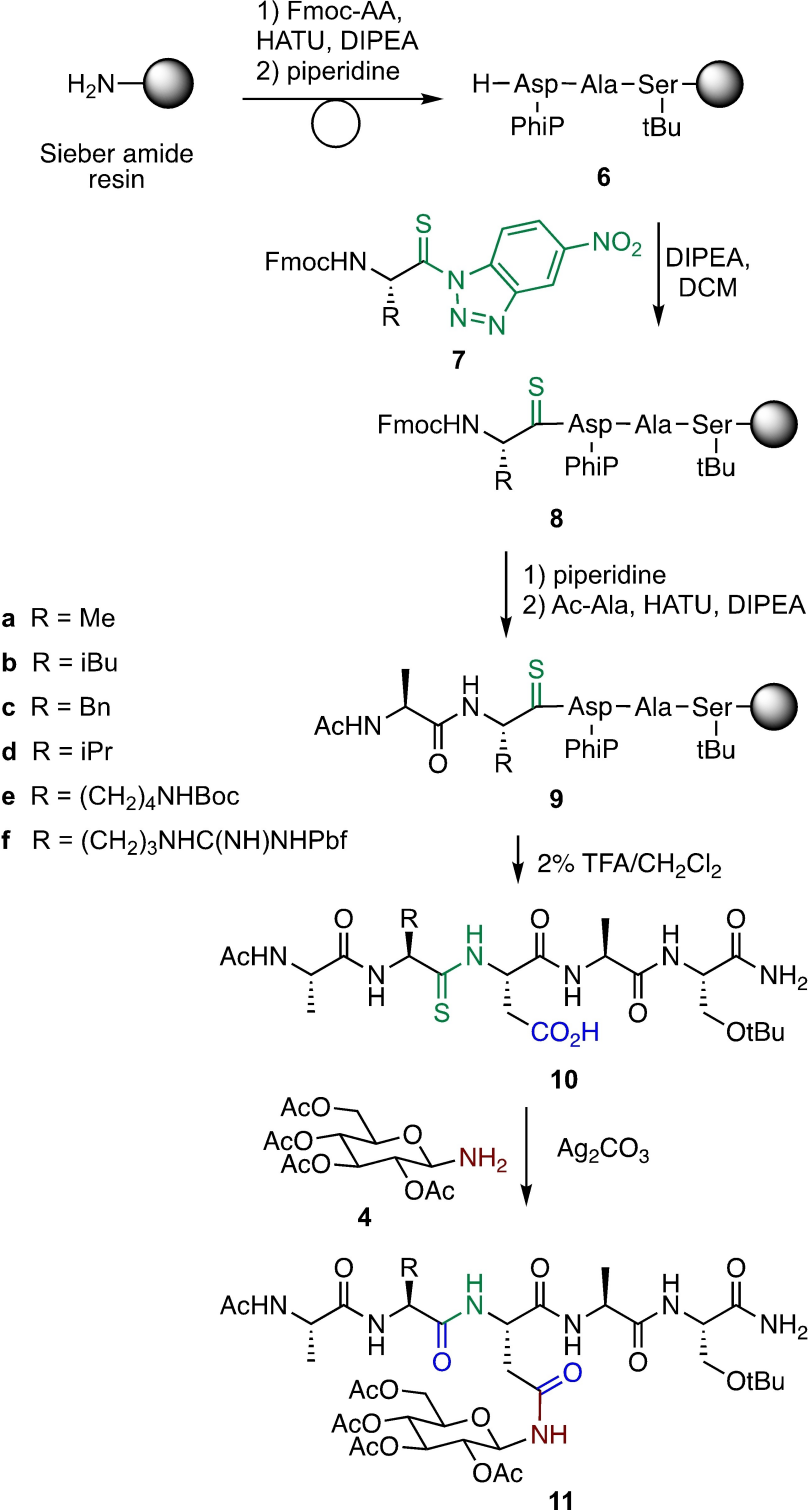
Synthesis Asn *N‐*glucosylated peptides **11 a**–**f**.

Conversion of thiopeptides **10 a**–**f** to the corresponding *N‐*glucosyl‐Asn adducts was investigated. The thiopeptides **10 a**–**f** were treated with Ag_2_CO_3_ and aminosugar **4** under the previously optimized conditions. Conversion of the thiopeptides to the corresponding glucosyl‐Asn adducts typically proceeded to >80 %, with trace amounts of the peptide oxo(amide) recovered. Following RP‐HPLC purification, the *N‐*glucosylated peptides **11 a**–**f** were obtained in good overall yields. The glucosyl peptides were characterized by NMR spectroscopy and HRMS, showing only a single product obtained for each glycopeptide (Scheme 4).

We next applied this method to synthesis of an *N‐*GlcNAc glycopeptide: *N‐*GlcNAc glycopeptides can be converted to complex glycopeptides through enzyme‐mediated glycosylations.[^33^, ^34^] Accordingly, the coupling of 1‐amino‐1‐deoxy‐*N‐*acetylglucosamine **12** with thiopeptide **10 c** afforded *N‐*GlcNAc glycopeptide **13** in 34 % yield after HPLC purification (Scheme 5).

**Scheme 5:**
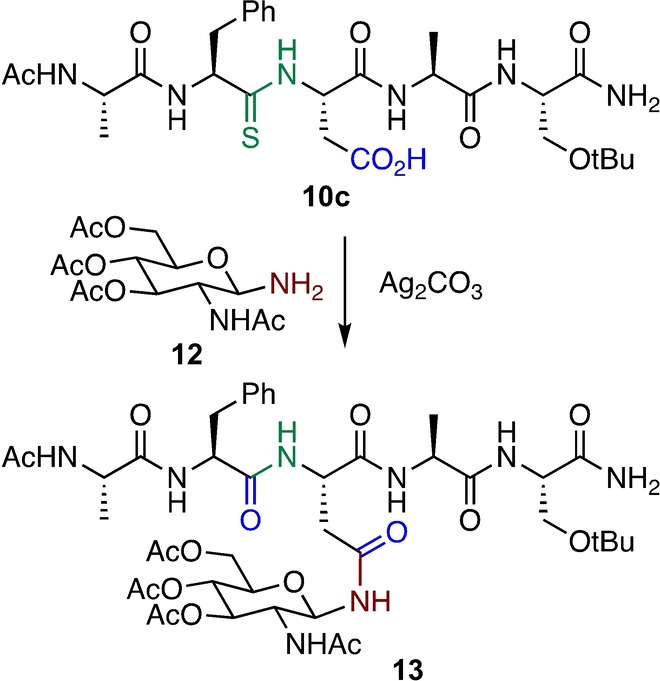
Synthesis of Asn *N‐*GlcNAc peptide.

With the successful preparation of *N‐*Glc/*N‐*GlcNAc peptides, the scope of the Ag^I^‐promoted coupling to effect site‐specific glycosylation was investigated using the central fragment of CSF114(Glc). The CSF114(Glc) glycopeptide contains one *N‐*glucosyl asparagine and has been developed as a biomarker for the diagnosis and monitoring of multiple sclerosis.^[5]^ The CSF114(4–10) fragment contains both aspartic acid and glutamic acid residues. The synthesis of the precursor peptide thioamides **14 a**–**d** was accomplished according to the general procedure used to prepare **10** (see Supporting Information). These peptides all contain free carboxylate sides chains at both Glu‐2 and Asp‐4.

Investigation of the glycosylation procedure was initiated with the Gly‐5‐Ala‐6 peptide thioamide **14 a** (Scheme 6). Employing the optimized glycosylation conditions, the *N‐*glucosylated peptide **15 a** was generated in reasonable yield with no evidence for reaction at the glutamate to generate an *N‐*glycosylated glutamine byproduct, indicating the process is site specific for the aspartate adjacent the backbone thioamide. The Phe‐5‐Ala‐6 analogue **14 b** gave the corresponding Asn *N‐*glycosylated peptide **15 b** in 29 % yield after HPLC purification. Intriguingly, when the native CSF114(4–10) sequence possessing a protected His(Ts)‐6 residue (**14 c**) was employed, only a trace of the *N‐*glycosylated peptide **15 c** was detected, with the major product being conversion of the thioamide to the oxoamide. Nevertheless, subsequent investigation revealed that incorporation of an unprotected His‐6 residue (**14 d**) led to a moderate yield of the glycopeptide **15 d** under the previously optimized conditions. Further investigations demonstrated that use of a greater excess of the aminosugar **4** (7 equiv) led to a significantly improved yield of 39 % of **15 d** after preparative RP‐HPLC, indicative of an increased rate of conversion of the cyclic isoimide intermediate to the N‐glycosylated product relative to hydrolysis of the isoimide (due to adventitious water) to generate the oxoamide byproduct. The excess aminosugar could be recovered.

**Scheme 6:**
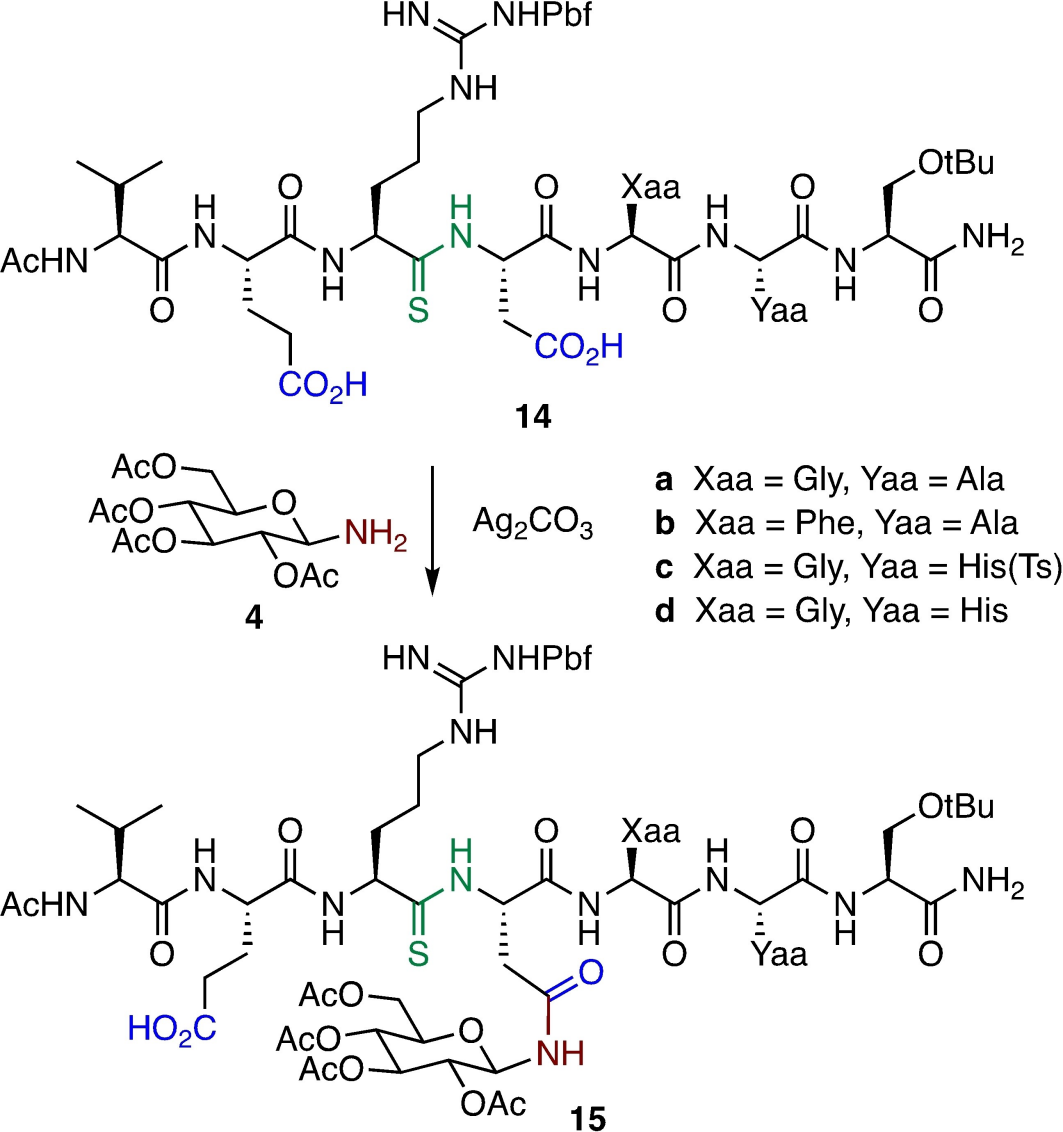
Site‐specific generation of *N‐*Glc Asn residue in CSF114 analogues.

In conclusion, a single‐atom (O→S) substitution of a linear peptide, through incorporation of a thioamide *N‐*terminal to an Asp residue, facilitates a Ag^I^‐promoted, site‐specific generation of *N‐*glycosylated Asn residues in peptides. This method overcomes drawbacks of existing methods such as site selectivity and aspartimidation. The results of this study and our related work[^18^, ^19^, ^20^] suggest that the method is amenable to peptides incorporating a wide variety of functionalized side chains, including unprotected His, Lys, Glu, Ser, Thr and protected residues including Cys. Further investigations including the incorporation of more complex glycans and application to on‐resin glycosylations are ongoing.

## Conflict of interest

The authors declare no conflict of interest.

## Supporting information

As a service to our authors and readers, this journal provides supporting information supplied by the authors. Such materials are peer reviewed and may be re‐organized for online delivery, but are not copy‐edited or typeset. Technical support issues arising from supporting information (other than missing files) should be addressed to the authors.

Supporting Information

## Data Availability

The data that support the findings of this study are available in the Supporting Information of this article.
