# Supplementary material for: Site Specific Preparation of N‐Glycosylated Peptides: Thioamide‐Directed Activation of Aspartate
Source: Angew Chem Int Ed Engl. 2022 Sep 21;61(43):e202210367. doi: 10.1002/anie.202210367 (PMC9826000; doi:10.1002/anie.202210367)
Supplement: Supplementary file 1 — Supporting Information [file ANIE-61-0-s001.pdf]

Angewandte Chemie
Eine Zeitschrift der Gesellschaft Deutscher Chemiker

Supporting Information

Site Specific Preparation of *N*-Glycosylated Peptides: Thioamide-Directed Activation of Aspartate

*A. B. Taresh, C. A. Hutton**

Supporting Information

Contents

General Information	S2
General Procedures	S3–S5
Experimental Procedures	S5–S26
NMR spectra of **S1**–**S7**, **10a**–**f**	S27–S41
NMR, mass spectra of **11a**–**f, 13**	S42–S48
HPLC traces and mass spectra of **14a**,**b**,**d** and **15a**,**b**,**d**	S49–S54
References	S55

General Information

Reactions requiring anhydrous conditions were carried out under an atmosphere of dry nitrogen or argon in flame- or oven-dried glassware. Anhydrous dichloromethane, tetrahydrofuran and dimethylformamide were obtained from solvent dispensing system where the solvent was dried by passage through two columns of neutral alumina. Most reagents were commercially available reagent grade chemicals and were used without further purification.

Analytical thin layer chromatography (TLC) was performed with aluminium-backed plates precoated with silica gel 60 F254 (0.2 mm), and chromatograms were visualised using was short- and long-wave UV light. Compounds were then stained with phosphomolybdic acid dip [phosphomolybdic acid (5 g), absolute ethanol (100 ml)]. Column chromatography was performed using silica gel (230–400 mesh); eluting solvents are reported as %volume/volume mixtures.

Analytical and preparative reverse phase HPLC (RP-HPLC) were performed using an Agilent 1200 series LC System. Analytical HPLC employed a Discovery C18 column (4.6 × 150 mm column, 5 µm particle size, flow rate of 1 mL min^{-1}). Preparative RP-HPLC employed a Phenomenex C18 column (21.2 × 150 mm, 5 µm particle size, flow rate 6 mL min $^{-}$1). The mobile phase consisted of eluents A (0.1% TFA in water) and B (0.1% TFA in acetonitrile). The results were analyzed on Agilent ChemStation version B.01.03 software.

^{1}H NMR spectra were recorded using an Agilent 500 (500 MHz) or a Varian Unity Inova 400 (400 MHz). Spectra were obtained in $CDCl_3$ (7.26) or d_6 DMSO (2.50). The spectra are reported as: parts per million (ppm) downfield shift, relative to the residual solvent peak; relative integral, multiplicity (s = singlet, br = broad, d = doublet, t = triplet, q = quartet, dd = doublet of doublets, dt = doublet of triplets, dq doublet of quartets, m = multiplet) and coupling constant (*J* in Hz). ^{13}C NMR spectra were recorded using an Agilent 500 (125 MHz) or a Varian Unity Inova 400 (101 MHz). Chemical shifts (δ) are reported in parts per million (ppm) relative to the internal standard of the solvent peak; $CDCl_3$ (77.16), and DMSO (39.52). Mass spectra were obtained using an MSFP OrbiTRAP infusion Mass Spectrometer.

General Procedures

Synthesis of of thionitrobenzotriazolides (7a–f)[1,2]

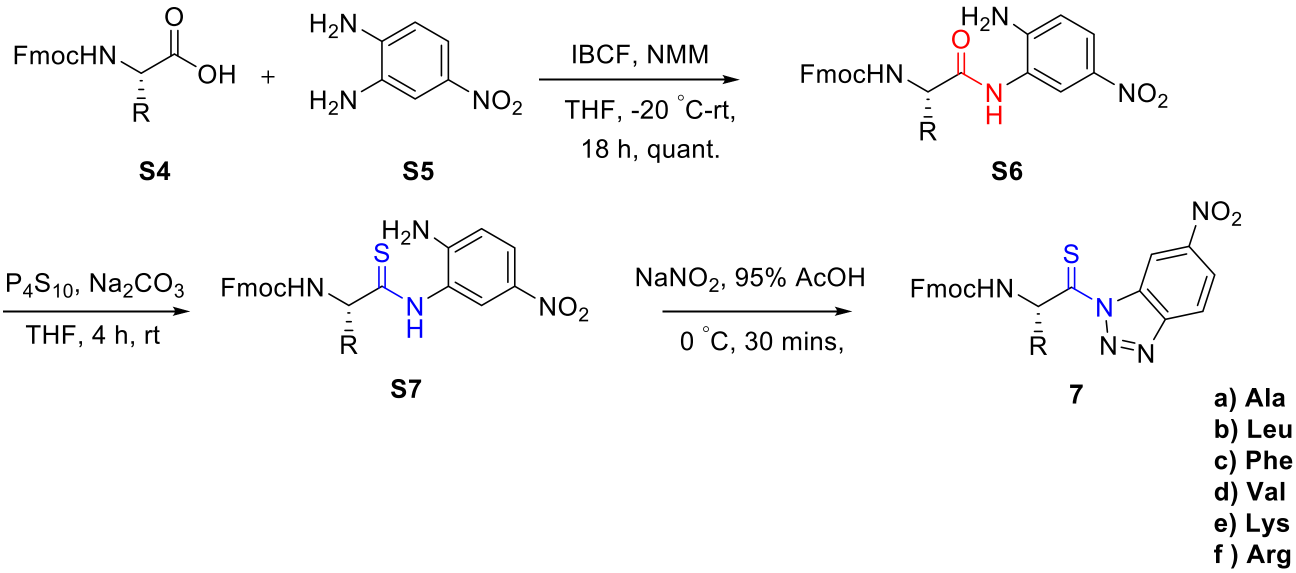

General Procedure A: Synthesis of anilides (7a–f)

A solution of Fmoc-amino acid **S4** (5.0 mmol) in dry tetrahydrofuran (50 mL) was cooled to –20 °C, and was added dropwise N-methylmorpholine (NMM) (1.1 mL, 10 mmol) and isobutyl chloroformate (IBCF) (0.714 mL, 5.5 mmol), and stirred for 15 min under Ar flow. Then, diaminonitrobenzene **S5** (0.842 g, 5.5 mmol) was added and the mixture was stirred at –20 °C for 2 h, then at room temperature overnight under Ar. The solvent was removed under reduced pressure, and the residue was dissolved in DMF (20mL). Upon addition of brine (100 mL) into the crude mixture, a yellow solid precipitated. The precipitant was filtered and rinsed with cold water. The crude mixture was purified by flash chromatography (5% methanol/dichloromethane) to give the title compounds **S6**.

General Procedure B: Formation of thioanilides (S7)

A solution of phosphorus pentasulfide (0.7 mmol) in dry tetrahydrofuran (10 mL) was added anhydrous sodium carbonate (0.7 mmol) and was stirred at room temperature for 1 h under argon. The reaction mixture was cooled to 0 °C and was added the amide **S6** (1.0 mmol). The resulting mixture was stirred at room temperature for 3.0 h. The mixture was filtered through a pad of Celite, and the solvent evaporated under reduced pressure. The residue was dissolved in ethyl acetate (25

mL), and was washed with aqueous sodium hydrogen carbonate solution (2 × 15 mL, 5% w/v) and brine (20 mL). The solvent was evaporated in reduced pressure and the residue was purified by flash chromatography (50% ethyl acetate/hexane) to give the title compounds (**S7**).

General Procedure C: Formation of nitrobenzotriazolides (7)

A thioanilide (**S7**) (0.2 mmol) was dissolved in 95% glacial acetic acid (2.0 mL), and cooled to 0 °C. Sodium nitrite (0.3 mmol) was added and the mixture was stirred at 0 °C for 30 min. The reaction was quenched with ice-water (10 mL), and the precipitate was isolated by filtration and washed with cold water (10 mL x 3). The crude product was dried to give the nitrobenzotriazolide (**7**) which was used in the next step without purification.

General Procedure D: Synthesis of thiopeptides on solid support

Thiopeptides were synthesised using standard Fmoc SPPS coupling methods on Sieber amide resin (100-200 mesh, 0.51 mmol/g loading) in (12 mL) fritted syringes. The resin was swelled in DMF (6 mL) for 1 hour, then the solvent was drained. All peptides were synthesised on 0.1 mmol scale using Fmoc-amino acids (4 equiv; 0.4 mmol for a 0.1mmol scale) that were activated using HATU (4 equiv.) in the presence of DIPEA (8 equiv.). The coupling reactions were stirred for 1 hour at room temperature. Fmoc removal was performed using 20% v/v piperidine in DMF (2 x 5 minutes). The peptide was elongated up to the thioamide position. Before thioamide coupling, the resin was washed with dry DCM (2 x 5 mL). Next, the required Fmoc-amino acid thiobenzotriazolide **7** (0.15 mmol., 1.5 equiv.) and DIPEA (0.15 mmol, 1.5 equiv.) in dry DCM (5 mL) were added and the reaction was stirred for 1 hour. The remaining amino acids were coupled using standard SPPS. The peptide was cleaved from the resin using (1% TFA in DCM, 6 mL) solution for 30 minutes. The solution was drained and collected and this process was repeated. The combined solutions were concentrated to a yellow oil under a stream of nitrogen. The crude peptide was precipitated by treatment with cold diethyl ether (10 mL) and the mixture was centrifuged at 300 rpm for 5 minutes. The collected

precipitate was washed with cold diethyl ether (3x 10 mL) followed by centrifugation. The thiopeptides was purified by RP-HPLC using gradient elution with buffer B 5–50% over 30 minutes, monitoring at a wavelength of 214 nm.

General Procedure E: Synthesis of glycopeptides

To a solution of the thiopeptide (1.0 equiv.) in dichloromethane:acetonitrile (1:1) (2 mL/0.1 mmol), was added silver carbonate (1.2 equiv.) and aminosugar **4**[3] (4.0 equiv.) The mixture was stirred at room temperature for 6 h. The black Ag_2S precipitate was removed by centrifugation. The glycopeptide was purified by RP-HPLC using gradient elution with buffer B 5–70% over 30 minutes, monitoring at a wavelength of 214 nm.

Experimental Procedures

Boc-Phe-Asp(OMe)-O*t*Bu (S2)

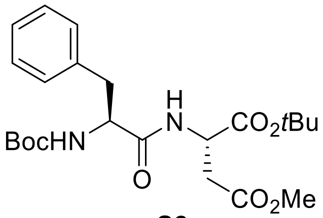

S2

To a solution of *N*-Boc-Phe-OH (266 mg, 1.0 mmol) and H-Asp(OMe)-OtBu hydrochloride (240 mg, 1.0 mmol) in DMF (10 mL) at 0 °C was added EDC.Cl (1.1 mmol), HOBt (1.1 mmol) and DIEA (2.2 mmol), and was stirred for 15 minute The reaction mixture was then stirred at room temperature overnight. The mixture was diluted with EtOAc (30 mL) and water (10 mL). The aqueous layer was extracted with EtOAc (2 × 20 mL) and the combined organic extracts were washed with an aqueous HCl solution (30 mL, 1N), saturated aqueous $NaHCO_3$ solution (30 mL), brine (30 mL) and dried with $MgSO_4$. The solvent was removed in reduced pressure and the crude product was purified by column chromatography (5% MeOH/DCM) to afford the title compound **S2** (415 mg, 92%) as colorless crystals; R_f 0.33 (5% MeOH/DCM). **^{1}H NMR** (400 MHz, $CDCl_3$) δ 7.34–7.10 (m, 5H), 6.82 (d, *J*=7.5 Hz, 1H), 4.93 (d, *J*=8.2 Hz, 1H), 4.63 (m, 1H), 4.39 (m, 1H), 3.65 (s, 3H), 3.08 (m, 2H), 2.93 (dd, *J*=16.9, 4.3 Hz, 1H), 2.79 (dd, *J*=16.8, 5.0 Hz, 1H), 1.43 (s, 9H), 1.39 (s, 9H).**^{13}C NMR** (101 MHz, $CDCl_3$) δ 171.0, 170.9, 169.0, 155.2, 136.3, 129.3, 128.6, 126.9, 82.6, 80.1, 55.4, 51.8, 49.3, 38.3, 36.3, 28.2, 27.8.**MS** (ESI) *m/z* 451 [$(M+H)^+$,100%]. **HRMS** (ESI, $[M+H]^+$)calcd. for $C_{23}H_{35}N_2O_7$ 451.2439, found 451.2441.

Boc-Phe[S]-Asp(OMe)-O*t*Bu (S3)

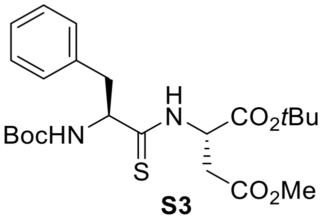

S3

To a solution of the dipeptide **S2** (193 mg, 0.49 mmol) in toluene (5 mL) was added Lawesson's reagent (243 mg, 0.6 mmol). The reaction mixture was heated at 60 °C for 2 h. The solvent was evaporated under reduced pressure. Purification of the residue by column chromatography (50% EtOAc/ hexane) gave the title compound **S3** (165mg, 82%) as a light yellow oil; R_f 0.50 (50% EtOAc/ hexane. **^{1}H NMR** (400 MHz, $CDCl_3$) δ 8.44 (d, *J*=7.2 Hz, 1H), 7.34–7.09 (m, 5H), 5.16 (m, 1H), 5.07 (m, 1H), 4.73–4.52 (m, 1H), 3.63 (s, 3H), 3.17 (m, 2H), 3.08 (dd, *J*=17.1, 4.9 Hz, 1H), 2.99 (dd, *J*=17.1, 4.0 Hz, 1H), 1.41 (s, 9H), 1.38 (s, 9H). **^{13}C NMR** (101 MHz, $CDCl_3$) δ 202.8, 170.8, 168.1, 154.9, 136.2, 129.2, 128.6, 127.0, 83.2, 80.4, 62.6, 54.1, 51.9, 41.5, 34.7, 28.2, 27.8. **MS** (ESI) *m/z* 467 [$(M+H)^+$,100%]. **HRMS** (ESI, $[M+H]^+$) calcd. for $C_{23}H_{35}N_2O_6S$ 467.2210, found 467.2202.

Boc-Phe[S]-Asp-O*t*Bu (3)

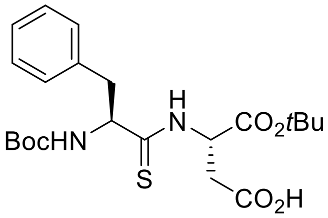

1

To a solution of **S3** (150 mg, 0.32 mmol) in a mixture of MeOH: H_2O (1:1) (4 mL) was cooled to 0 °C, and added $LiOH.H_2O$ (40 mg,0.96 mmol). The reaction mixture was stirred for 10 minutes at 0 °C and 2 hours at room temperature. The solution was concentrated and was diluted with water (10 mL) then acidified to pH 4 with aqueous hydrochloric acid (1 M). The solution was extracted with EtOAc (3×10 mL) and washed with water (20 mL), brine (20 mL) and dried with sodium sulfate. The solvent was evaporated in reduced pressure to give the crude acid **3** which was used in the next step

without further purification. **MS** (ESI) *m/z* 453 [$(M+H)^+$,100%]; **HRMS** (ESI, $[M+H]^+$) calcd. for $C_{22}H_{33}N_2O_6S$ 453.2054, found 453.2057.

Boc-Phe-Asn(Glc(Ac)$_4$)-O*t*Bu (5)

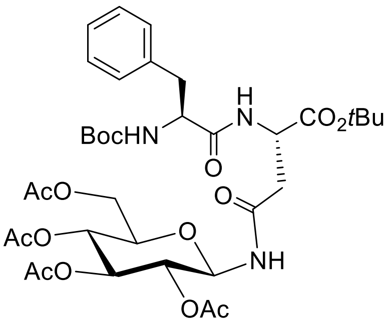

A solution of **3** (46 mg, 0.1 mmol) in (DCM:CH_3CN) (1:1) (2 mL) was added silver carbonate (33 mg, 0.12 mmol) and aminosugar **4** (139 mg, 0.4 mmol) and stirred at room temperature for 6 h. The solvents were evaporated and the residue purified by flash chromatography (10% methanol/dichloromethane) to give the compound **5** (59 mg, 77%) as a white solid. **^{1}H NMR** (400 MHz, $CDCl_3$) δ 7.36–7.17 (m, 6H), 7.14 (d, *J*=7.5 Hz, 1H), 6.79 (d, *J*=9.4 Hz, 1H), 5.28 (t, *J*=9.4 Hz, 1H), 5.18 (t, *J*=9.3 Hz, 1H), 5.11 (m, 1H), 5.03 (t, *J*=9.7 Hz, 1H), 4.95 (t, *J*=9.5 Hz, 1H), 4.62 (m, 1H), 4.52–4.30 (m, 2H), 4.02 (d, *J*=12.2 Hz, 1H), 3.77 (m, 1H), 3.17 (dd, *J*=14.0, 5.2 Hz, 1H), 2.96 (m, 1H), 2.85–2.65 (m, 2H), 2.05 (s, 3H), 2.04 (s, 3H), 2.02 (s, 3H), 2.00 (s, 3H), 1.44 (s, 9H), 1.37 (s, 9H). **^{13}C NMR** (101 MHz, $CDCl_3$) δ 170.8, 170.8, 170.0, 169.6, 169.0, 155.4, 136.4, 129.3, 129.2, 128.6, 127.2, 127.0, 82.8, 78.1, 73.8, 72.8, 70.5, 68.1, 61.5, 55.6, 49.6, 38.2, 38.0, 28.2, 27.8, 20.7, 20.7, 20.6. **MS** (ESI) *m/z* 766 [$(M+H)^+$,100%]. **HRMS** (ESI, $[M+H]^+$) calcd. for $C_{36}H_{52}N_3O_{15}$ 766.3393, found 766.3393.

Fmoc-Ala 2-amino-5-nitroanilide (S6a)

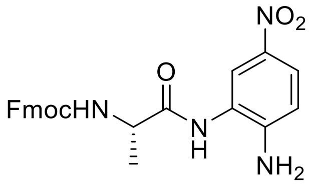

The title compound **S6a** was prepared from **S4a** (1.557 g, 5 mmol) according to General Procedure A. After purification The title compound **S6a** was isolated as a yellow solid (2.054 g, 92%); R_f 0.45 (5% MeOH/DCM). **^{1}H NMR** (400 MHz, DMSO) δ 9.35 (s, 1H), 8.16 (d, *J*=2.7 Hz, 1H), 7.91–7.80 (m, 3H), 7.77–7.67 (m, 3H), 7.40 (t, *J*=7.5 Hz, 2H), 7.32 (t, *J*=7.4 Hz, 2H), 6.75 (d, *J*=9.1 Hz, 1H), 6.42 (brs, 2H), 4.35–4.27 (m, 2H), 4.26–4.16 (m, 2H), 1.32 (d, *J*=7.0 Hz, 3H). **^{13}C NMR** (101 MHz, DMSO) δ 172.6, 156.5, 149.9, 144.3, 144.2, 141.2, 135.9, 128.1, 127.5, 125.8, 125.7, 123.6, 122.3, 121.6, 120.6, 114.0, 66.2, 51.1, 47.1, 18.1. **MS** (ESI) *m/z* 447 [(M+H)$^+$,100%]; **HRMS** (ESI, [M+H]$^+$) calcd. for $C_{24}H_{23}N_4O_5$ 447.1663, found 447.1667.[1,2]

Fmoc-Leu 2-amino-5-nitroanilide (S6b)

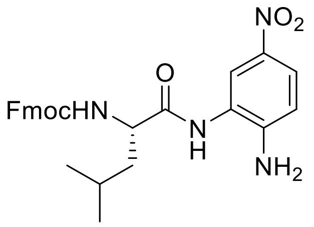

The title compound **S6b** was prepared from **S4b** (1.767 g, 5 mmol) according to General Procedure A. After purification The title compound **S6b** was isolated as a yellow solid (2.247 g, 92%); R_f 0.42 (5% MeOH/DCM). **^{1}H NMR** (400 MHz, DMSO) δ 9.39 (s, 1H), 8.15 (d, *J*=2.6 Hz, 1H), 7.91–7.82 (m, 3H), 7.75–7.66 (m, 3H), 7.44–7.35 (m, 2H), 7.35–7.26 (m, 2H), 6.75 (d, *J*=9.1 Hz, 1H), 6.40 (brs, 2H), 4.37–4.14 (m, 4H), 1.68 (m, 1H), 1.62–1.50 (m, 2H), 0.92 (d, *J*=6.5 Hz, 3H), 0.90 (d, *J*=6.5 Hz, 3H). **^{13}C NMR** (101 MHz, DMSO) δ 172.5, 156.7, 149.8, 144.3, 144.2, 141.2, 135.9, 128.1, 127.5, 125.7, 123.6, 122.3, 121.6, 120.6, 114.1, 71.0, 66.1, 54.0, 47.1, 24.7, 23.5, 22.0, 19.4. **MS** (ESI) *m/z* 489 [(M+H)$^+$,100%]; **HRMS** (ESI, [M+H]$^+$) calcd. for $C_{27}H_{29}N_4O_5$ 489.2132, found 489.2135. [1,2]

Fmoc-Phe 2-amino-5-nitroanilide (S6c)

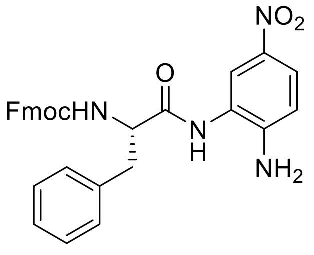

The title compound **S6c** was prepared from **S4c** (1.937 g, 5 mmol) according to General Procedure A. After purification The title compound **S6c** was isolated as a yellow solid (2.22 g, 85%); R_f 0.32 (5% MeOH/DCM). **^{1}H NMR** (400 MHz, DMSO) δ 9.41 (s, 1H), 8.04 (d, *J*=2.6 Hz, 1H), 7.92–7.79 (m, 4H), 7.66 (t, *J*=6.5 Hz, 2H), 7.39 (t, *J*=7.2 Hz, 2H), 7.35–7.19 (m, 7H), 6.73 (d, *J*=9.1 Hz, 1H), 6.32 (brs, 2H), 4.42 (m, 1H), 4.27–4.10 (m, 3H), 3.09 (dd, *J*=13.7, 5.7 Hz, 1H), 2.93 (dd, *J*=13.7, 9.3 Hz, 1H). **^{13}C NMR** (101 MHz, DMSO) δ 171.5, 156.5, 149.9, 144.2, 144.2, 141.1, 138.2, 135.9, 129.8, 128.6, 128.1, 127.5, 126.9, 125.8, 125.7, 123.7, 122.4, 121.4, 120.6, 114.0, 66.2, 57.0, 47.0, 40.6, 40.4, 40.2, 40.0, 39.8, 39.5, 39.3, 37.6. **MS** (ESI) *m/z* 523 [$(M+H)^+$,100%]; **HRMS** (ESI, $[M+H]^+$) calcd. for $C_{30}H_{27}N_4O_5$ 523.1976, found 523.1972.[1,2]

Fmoc-Val 2-amino-5-nitroanilide (S6d)

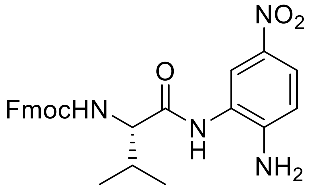

The title compound **S6d** was prepared from **S4d** (1.697 g, 5 mmol) according to General Procedure A. After purification The title compound **S6b** was isolated as a yellow solid (2.112 g, 89%); R_f 0.41 (5% MeOH/DCM).**^{1}H NMR** (400 MHz, DMSO) δ 9.40 (s, 1H), 8.22 (d, *J*=2.6 Hz, 1H), 7.92–7.82 (m, 3H), 7.79–7.67 (m, 3H), 7.44–7.35 (m, 2H), 7.35–7.27 (m, 2H), 6.76 (d, *J*=9.0 Hz, 1H), 6.41 (brs, 2H), 4.35–4.18 (m, 3H), 4.01 (t, *J*=7.8 Hz, 1H), 2.07 (h, *J*=6.9 Hz, 1H), 0.95 (d, *J*=1.9 Hz, 3H), 0.94 (d, *J*=1.9 Hz, 3H). **^{13}C NMR** (101 MHz, DMSO) δ 171.5, 157.0, 149.4, 144.3, 144.2, 141.2, 136.0, 128.1, 127.5, 125.8, 123.5, 121.7, 121.6, 120.6, 114.2, 66.3, 61.4, 47.1, 30.3, 19.7, 19.1. **MS** (ESI)

m/z 475 [$(M+H)^+$,100%]; **HRMS** (ESI, $[M+H]^+$) calcd. for $C_{26}H_{27}N_4O_5$ 475.1976, found 475.1977.[1,2]

Fmoc-Lys(Boc) 2-amino-5-nitroanilide (S6e)

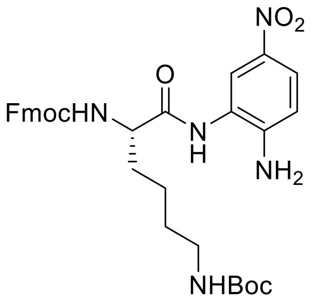

The title compound **S6e** was prepared from **S4e** (2.343 g, 5 mmol) according to General Procedure A. After purification The title compound **S6e** was isolated as a yellow solid (2.747 g, 91%); R_f 0.38 (5% MeOH/DCM).**^{1}H NMR** (400 MHz, DMSO-*d*6) δ 9.36 (s, 1H), 8.18 (d, *J*=2.7 Hz, 1H), 7.91–7.81 (m, 3H), 7.76–7.66 (m, 3H), 7.44–7.36 (m, 2H), 7.35–7.28 (m, 2H), 6.81–6.71 (m, 2H), 6.40 (brs, 2H), 4.33–4.18 (m, 3H), 4.12 (m, 1H), 2.91 (d, *J* = 6.6 Hz, 2H), 1.80–1.57 (m, 2H), 1.44–1.31 (m, 13H). **^{13}C NMR** (101 MHz, DMSO-*d*6) δ 172.1, 156.8, 156.0, 149.7, 144.3, 144.2, 141.2, 135.9, 128.1, 127.5, 125.8, 123.6, 122.1, 121.6, 120.6, 114.1, 77.8, 66.2, 55.7, 47.1, 31.6, 29.7, 28.7, 23.4. **MS** (ESI) *m/z* 604 [$(M+H)^+$,100%]; **HRMS** (ESI, $[M+H]^+$ calcd. for $C_{32}H_{38}N_5O_7$ 604.2766, found 604.2768.[1,2]

Fmoc-Arg(Pbf) 2-amino-5-nitroanilide (S6f)

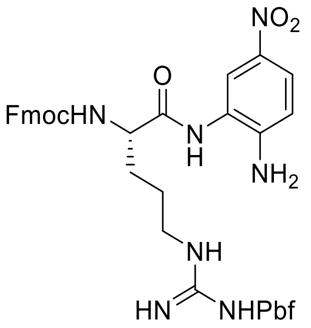

The title compound **S6f** was prepared from **S4f** (3.244 g, 5 mmol) according to General Procedure A. After purification The title compound **S6f** was isolated as a yellow solid (3.724 g, 95%); R_f 0.38 (5%

MeOH/DCM). ^{1}**H NMR** (400 MHz, DMSO-d_6) δ 9.39 (s, 1H), 8.18 (d, J=2.6 Hz, 1H), 7.93–7.80 (m, 3H), 7.79–7.65 (m, 3H), 7.39 (t, J=7.5 Hz, 2H), 7.31 (t, J=7.4 Hz, 2H), 6.75 (d, J=9.1 Hz, 1H), 6.42 (brs, 2H), 4.34–4.26 (m, 2H), 4.22 (m, 1H), 4.14 (m, 1H), 3.11–3.01 (m, 2H), 2.90 (s, 2H), 2.46 (s, 3H), 2.40 (s, 3H), 1.96 (s, 3H), 1.74 (m, 1H), 1.59 (m, 1H), 1.48 (m, 2H), 1.37 (s, 6H). ^{13}**C NMR** (101 MHz, DMSO-d_6) δ 171.9, 157.9, 156.7, 156.5, 149.7, 144.3, 144.2, 141.2, 137.7, 135.9, 131.9, 128.1, 127.5, 125.7, 124.8, 123.6, 122.2, 121.5, 120.6, 116.7, 114.1, 86.7, 66.2, 55.3, 47.1, 42.9, 29.3, 28.7, 19.4, 18.0, 12.7. **MS** (ESI) *m/z* 784 [(M+H)$^+$,100%]; **HRMS** (ESI, [M+H]$^+$ calcd. for $C_{40}H_{46}N_7O_8S$ 784.3123, found 784.3124.[1,2]

Fmoc-Ala[S] 2-amino-5-nitroanilide (S7a)

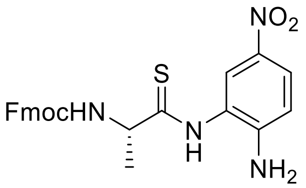

The title compound **S7a** was prepared from **S6a** (447 mg, 1.0 mmol) according to General Procedure B. After purification The title compound **S7a** was isolated as a yellow solid (379 mg, 82%); R_f 0.34 (50% ethyl acetate / hexane). ^{1}**H NMR** (400 MHz, DMSO-d_6) δ 11.23 (s, 1H), 7.97–7.91 (m, 2H), 7.90–7.88 (m, 2H), 7.87 (s, 1H), 7.74 (d, J=7.5 Hz, 1H), 7.71 (d, J=7.5 Hz, 1H), 7.45–7.36 (m, 2H), 7.35–7.27 (m, 2H), 6.77 (d, J=9.1 Hz, 1H), 6.37 (brs, 2H), 4.51 (m, 1H), 4.30 (m, 1H), 4.27–4.15 (m, 2H), 1.43 (d, J = 6.8 Hz, 3H). ^{13}**C NMR** (101 MHz, DMSO-d_6) δ 208.7, 156.6, 151.1, 144.3, 141.2, 144,1, 135.7, 128.1, 127.6, 125.8, 125.7, 125.4, 125.2, 122.9, 120.6, 114.3, 66.3, 57.4, 47.1, 20.7. **MS** (ESI) *m/z* 463 [(M+H)$^+$,100%]; **HRMS** (ESI, [M+H]$^+$ calcd. for $C_{24}H_{23}N_4O_4S$ 463.1435, found 463.1433. [1,2]

Fmoc-Leu[S] 2-amino-5-nitroanilide (S7b)

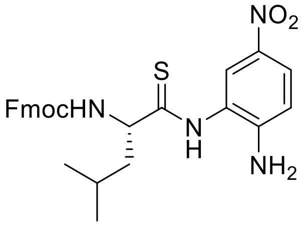

The title compound **S7b** was prepared from **S6b** (489 mg, 1.0 mmol) according to General Procedure B. After purification The title compound **S7b** was isolated as a yellow solid (429 mg, 85%); R_f 0.33 (50% ethyl acetate / hexane).**^{1}H NMR** (400 MHz, DMSO-*d*$_6$) δ 11.34 (s, 1H), 7.97–7.91 (m, 2H), 7.88 (d, *J*=7.5 Hz, 2H), 7.83 (d, *J*=2.6 Hz, 1H), 7.72 (dd, *J*=12.1, 7.5 Hz, 2H), 7.40 (t, *J*=7.4 Hz, 2H), 7.30 (t, *J*=7.4 Hz, 2H), 6.77 (d, *J*=9.1 Hz, 1H), 6.34 (brs, 2H), 4.49 (m, 1H), 4.31 (m, 1H), 4.27–4.15 (m, 2H), 1.81 – 1.60 (m, 3H), 0.95 (d, *J*=5.9 Hz, 3H), 0.92 (d, *J*=6.0 Hz, 3H). **^{13}C NMR** (101 MHz, DMSO-*d*$_6$) δ 208.8, 157.0, 151.1, 144.3, 144.0, 141.2, 135.8, 128.1, 127.5, 127.5, 125.9, 125.7, 125.4, 125.1, 122.9, 120.6, 114.4, 66.3, 60.1, 47.1, 43.2, 24.8, 23.3, 22.4. **MS** (ESI) *m/z* 505 [$(M+H)^+$,100%]; **HRMS** (ESI, $[M+H]^+$ calcd. for $C_{27}H_{29}N_4O_4S$ 505.1904, found 505.1903. [1,2]

Fmoc-Phe[S] 2-amino-5-nitroanilide (S7c)

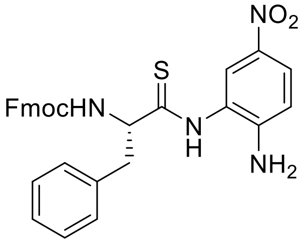

The title compound **S7c** was prepared from **S6c** (523 mg, 1.0 mmol) according to General Procedure B. After purification The title compound **S7c** was isolated as a yellow solid (469 mg, 87%); R_f 0.33 (50% ethyl acetate / hexane). **^{1}H NMR** (400 MHz, DMSO-*d*$_6$) δ 11.25 (s, 1H), 8.13 (d, *J*=6.6 Hz, 1H), 7.87 (d, *J*=7.6 Hz, 2H), 7.69 (t, *J*=7.7 Hz, 2H), 7.55 (d, *J*=2.7 Hz, 1H), 7.43–7.21 (m, 10H), 6.73 (d, *J*=9.2 Hz, 1H), 6.18 (brs, 2H), 4.67 (m, 1H), 4.28 (m, 1H), 4.18 (m, 2H), 3.20–3.02 (m, 2H). **^{13}C NMR** (101 MHz, DMSO-*d*$_6$) δ 207.3, 156.7, 151.0, 144.2, 144.0, 141.1, 137.8, 135.7, 130.0, 128.6, 128.1, 128.1, 127.6, 127.5, 127.0, 125.9, 125.7, 125.4, 125.0, 122.6, 120.6, 114.3, 66.3, 63.0, 47.0.

MS (ESI) *m/z* 539 [$(M+H)^+$,100%]; **HRMS** (ESI, $[M+H]^+$ calcd. for $C_{30}H_{27}N_4O_4S$ 539.1748, found 539.1747.[1,2]

Fmoc-Val[S] 2-amino-5-nitroanilide (S7d)

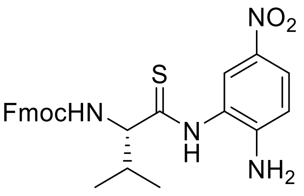

The title compound **S7d** was prepared from **S6d** (475 mg, 1.0 mmol) according to General Procedure B. After purification The title compound **S7d** was isolated as a yellow solid (392 mg, 82%); R_f 0.32 (50% ethyl acetate / hexane).**^{1}H NMR** (400 MHz, DMSO-d_6) δ 11.39 (s, 1H), 8.02 (d, *J*=6.9 Hz, 1H), 7.95 (dd, *J*=9.1, 2.7 Hz, 1H), 7.88 (d, *J*=7.5 Hz, 2H), 7.81 (d, *J*=2.6 Hz, 1H), 7.73 (dd, *J*=14.4, 7.4 Hz, 2H), 7.40 (t, *J*=7.4 Hz, 2H), 7.31 (t, *J*=7.4 Hz, 2H), 6.77 (d, *J*=9.1 Hz, 1H), 6.34 (brs, 2H), 4.37–4.26 (m, 2H), 4.26–4.15 (m, 2H), 4.10 (m, 1H), 2.13 (m, 1H), 1.04 (d, *J* = 6.5 Hz, 3H), 0.98 (d, *J* = 6.8 Hz, 3H). **MS** (ESI) *m/z* 491 [$(M+H)^+$,100%]; **HRMS** (ESI, $[M+H]^+$ calcd. for $C_{26}H_{27}N_4O_4S$ 491.1748, found 491.1749.[1,2]

Fmoc-Lys[S](Boc) 2-amino-5-nitroanilide (S7e)

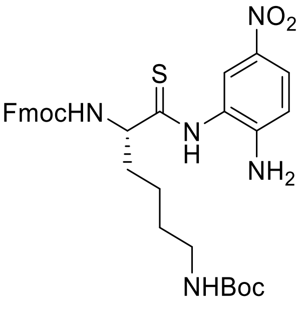

The title compound **S7e** was prepared from **S6e** (604 mg, 1.0 mmol) according to General Procedure B. After purification The title compound **S7e** was isolated as a yellow solid (527 mg, 85%); R_f 0.3 (50% ethyl acetate / hexane).**^{1}H NMR** (400 MHz, DMSO-d_6) δ 11.33 (s, 1H), 8.00–7.91 (m, 2H), 7.89 (s, 1H), 7.86 (d, *J*=4.1 Hz, 2H), 7.74 (d, *J*=7.5 Hz, 1H), 7.71 (d, *J*=7.5 Hz, 1H), 7.40 (t, *J*=7.5

Hz, 2H), 7.31 (t, *J*=7.4 Hz, 2H), 6.82 (6.82 (t, *J*=5.9, 5.3 Hz, 1H), 6.76 (d, *J*=9.2 Hz, 1H), 6.36 (brs, 2H), 4.38 (m, 1H), 4.30 (m, 1H), 4.26–4.16 (m, 2H), 2.92 (t, *J*=6.5 Hz, 2H), 1.90–1.65 (m, 2H), 1.45–1.30 (m, 13H). **^{13}C NMR** (101 MHz, DMSO-*d*6) δ 208.4, 157.0, 156.1, 151.1, 144.3, 144.0, 141.1, 135.7, 128.1, 127.6, 127.5, 125.9, 125.7, 125.5, 125.2, 122.8, 120.6, 114.3, 77.8, 66.3, 61.8, 60.2, 47.0, 33.9, 29.7, 28.7, 23.3, 21.2, 14.5. **MS** (ESI) *m/z* 620 [(M+H)$^+$,100%]; **HRMS** (ESI, [M+H]$^+$ calcd. for $C_{32}H_{38}N_5O_6S$ 620.2537, found 620.2536. [1,2]

Fmoc-Arg[S](Pbf) 2-amino-5-nitroanilide (S7f)

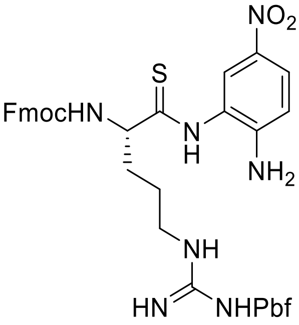

The title compound **S7f** was prepared from **S6f** (784 mg, 1.0 mmol) according to General Procedure B. After purification The title compound **124f** was isolated as a yellow solid (648 mg, 81%); R*f* 0.3 (50% ethyl acetate / hexane).**^{1}H NMR** (400 MHz, DMSO-*d*6) δ 11.33 (s, 1H), 7.99–7.91 (m, 2H), 7.88 (s, 1H), 7.86 (m, 2H), 7.73 (d, *J*=7.5 Hz, 1H), 7.70 (d, *J*=7.4 Hz, 1H), 7.40 (t, *J*=7.5 Hz, 2H), 7.30 (t, *J*=7.5 Hz, 2H), 6.77 (d, *J*=9.1 Hz, 1H), 6.36 (brs, 2H), 4.40 (q, *J*=6.9 Hz, 1H), 4.36–4.27 (m, 1H), 4.23 (d, *J*=6.1 Hz, 2H), 3.09 (q, *J*=6.7 Hz, 2H), 2.92 (s, 2H), 2.48 (s, 3H), 2.42 (s, 3H), 1.98 (s, 3H), 1.90–1.67 (m, 2H), 1.64–1.42 (m, 2H), 1.37 (s, 6H); **^{13}C NMR** (101 MHz, DMSO-*d*6) δ 207.9, 170.8, 157.9, 156.9, 156.5, 151.1, 144.3, 144.0, 141.2, 137.7, 135.8, 131.9, 128.1, 127.6, 127.5, 125.9, 125.7, 125.5, 125.1, 124.8, 122.7, 120.6, 116.7, 114.4, 86.7, 66.4, 61.5, 60.2, 47.1, 42.9, 31.7, 28.7, 21.2, 19.4, 18.1, 14.5, 12.7; **MS** (ESI) *m/z* 800 [(M+H)$^+$,100%]; **HRMS** (ESI, [M+H]$^+$ calcd. for $C_{40}H_{46}N_7O_7S_2$ 800.2895, found 800.2893. [1,2]

Ac-Ala-Ala[S]-Asp-Ala-Ser(*t*Bu)-NH2 (10a)

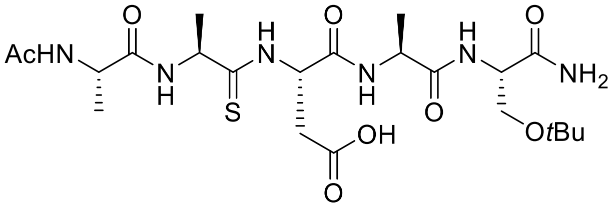

The title compound **10a** was prepared according to General Procedure D. After HPLC purification, the title compound **10a** was obtained as a white solid (14 mg, 25%).).**^{1}H NMR** (500 MHz, DMSO-d_6) δ 12.48 (brs, 1H), 10.02 (d, *J*=7.2 Hz, 1H), 8.12 (d, *J*=4.0 Hz, 1H), 8.11 (d, *J*=4.0 Hz, 1H), 8.02 (d, *J*=7.1 Hz, 1H), 7.62 (d, *J*=8.0 Hz, 1H), 7.18 (s, 1H), 7.11 (s, 1H), 5.12 (m, 1H), 4.62 (m, 1H), 4.29–4.16 (m, 3H), 3.47–3.41 (m, 2H), 2.82 (dd, *J*=17.0, 5.3 Hz, 1H), 2.73 (dd, *J*=16.9, 8.0 Hz, 1H), 1.83 (s, 3H), 1.26 (d, *J*=6.9 Hz, 3H), 1.20 (s, 3H), 1.18 (s, 3H), 1.10 (s, 9H). **^{13}C NMR** (126 MHz, DMSO-d_6) δ 206.1, 172.3, 172.0, 171.9, 169.7, 169.1, 73.2, 62.1, 55.2, 54.6, 53.6, 49.1, 40.6, 48.7, 35.5, 27.7, 22.9, 21.7, 18.4, 18.2. **MS** (ESI) *m/z* 547 [(M+H)$^+$,100%]. **HRMS** (ESI, [M+H]$^+$ calcd. for $C_{22}H_{39}N_6O_8S$ 547.2545, found 547.2547.

Ac-Ala-Leu[S]-Asp-Ala-Ser(*t*Bu)-NH2 (10b)

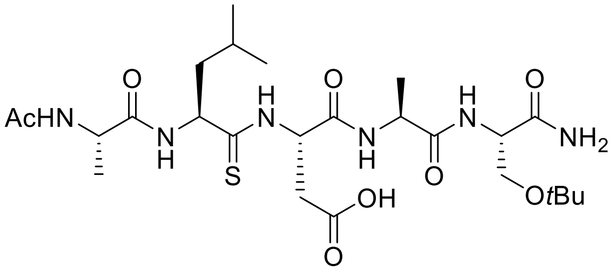

The title compound **10b** was prepared according to General Procedure D. After HPLC purification, the title compound **10b** was obtained as a white solid (9 mg, 16%).). **^{1}H NMR** (500 MHz, DMSO-d_6) δ 12.48 (brs, 1H), 10.07 (d, *J*=7.0 Hz, 1H), 8.58 (dd, *J*=4.3, 1.3 Hz, 1H), 8.08 (d, *J*=7.3 Hz, 1H), 8.04 (d, *J*=7.2 Hz, 1H), 7.91 (d, *J*=8.2 Hz, 1H), 7.64 (d, *J*=8.0 Hz, 1H), 7.20 (s, 1H), 7.11 (s, 1H), 5.10 (m, 1H), 4.67 (m, 1H), 4.29–4.20 (m, 2H), 4.19 (m, 1H), 3.52–3.47 (m, 2H), 2.82 (dd, *J*=17.0, 5.3 Hz, 1H), 2.72 (dd, *J*=17.1, 8.2 Hz, 1H), 1.82 (s, 3H), 1.61 (m, 1H), 1.49 (m, 2H), 1.19 (s, 3H),

1.17 (s, 3H), 1.10 (s, 9H), 0.88–0.82 (m, 6H). **MS** (ESI) *m/z* 589 [$(M+H)^+$,100%]. **HRMS** (ESI, $[M+H]^+$ calcd. for $C_{25}H_{45}N_6O_8S$ 589.3014, found 589.3012.

Ac-Ala-Phe[S]-Asp-Ala-Ser(*t*Bu)-NH2 (10c)

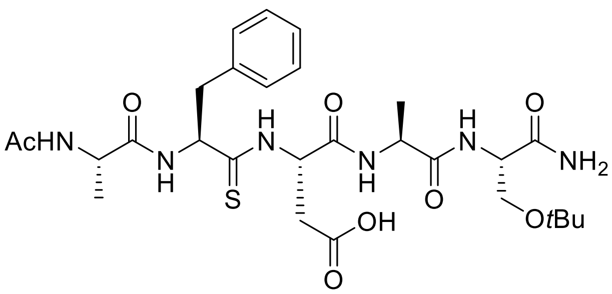

The title compound **10c** was prepared according to General Procedure D. After HPLC purification, the title compound **10c** was obtained as a white solid (14 mg, 23%).**^{1}H NMR** (500 MHz, DMSO-*d*6) δ 12.49 (brs, 1H), 10.14 (d, *J*=7.2 Hz, 1H), 8.34 (d, *J*=8.3 Hz, 1H), 8.06 (d, *J*=7.0 Hz, 1H), 7.93 (d, *J*=7.0 Hz, 1H), 7.57 (d, *J*=8.0 Hz, 1H), 7.30–7.12 (m, 7H), 5.21–5.14 (m, 1H), 4.87–4.80 (m, 1H), 4.28–4.16 (m, 3H), 3.45–3.41 (m, 3H), 3.13 (dd, *J*=13.9, 3.4 Hz, 1H), 2.87–2.73 (m, 3H), 1.78 (s, 3H), 1.20 (d, *J*=7.0 Hz, 3H), 1.10 (s, 9H), 0.91 (d, *J*=7.0 Hz, 3H). **^{13}C NMR** (126 MHz, DMSO) δ 204.71, 172.99, 172.03, 171.90, 171.87, 169.81, 169.18, 138.15, 129.69, 128.41, 126.78, 73.17, 62.14, 60.58, 55.54, 53.61, 49.18, 48.76, 40.98, 35.65, 27.68, 22.78, 18.21. **MS** (ESI) *m/z* 623 [$(M+H)^+$,100%]. **HRMS** (ESI, $[M+H]^+$ calcd. for $C_{28}H_{43}N_6O_8S$ 623.2858, found 623.2858.

Ac-Ala-Phe[S]-Asp-Ala-Ser(*t*Bu)-NH2 (10d)

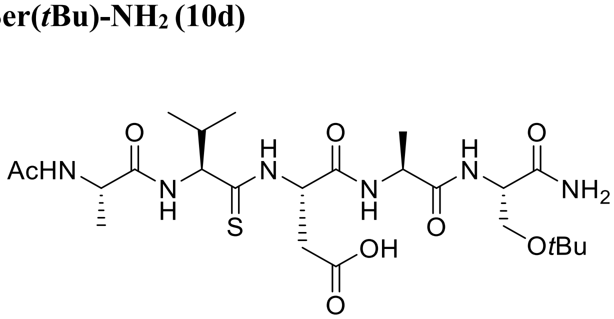

The title compound **10d** was prepared according to General Procedure D. After HPLC purification, the title compound **10d** was obtained as a white solid (10 mg, 17%). **^{1}H NMR** (500 MHz, DMSO-*d*6) δ 12.45 (brs, 1H), 9.85 (d, *J*=7.4 Hz, 1H), 8.37 (d, *J*=7.4 Hz, 1H), 8.25 (d, *J*=6.2 Hz, 1H), 7.69 (d,

J=7.0 Hz, 1H), 7.47 (d, *J*=8.2 Hz, 1H), 7.12 (m, 2H), 5.16 (m, 1H), 4.56 (m, 1H), 4.28–4.15 (m, 3H), 3.49 (dd, *J*=9.2, 5.5 Hz, 1H), 3.43 (m, 1H), 2.88–2.83 (m, 2H), 1.82 (s, 3H), 1.61 (m, 1H), 1.21–1.15 (m, 6H), 1.10 (s, 9H), 0.87 (d, *J*=6.2 Hz, 3H), 0.83 (d, *J*=6.1 Hz, 3H). **MS** (ESI) *m/z* 623 [$(M+H)^+$,100%]. **HRMS** (ESI, $[M+H]^+$ calcd. for $C_{24}H_{43}N_6O_8S$ 575.2858, found 575.2856.

Ac-Ala-Lys[S](Boc)-Asp-Ala-Ser(*t*Bu)-NH2 (10e)

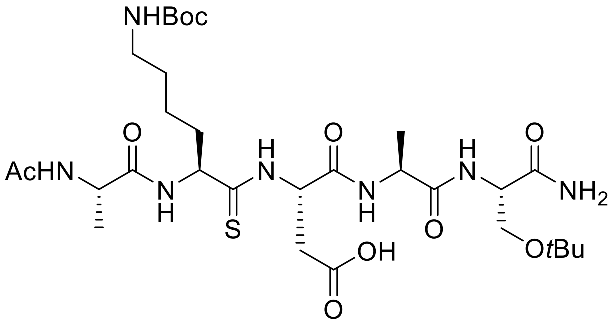

The title compound **10e** was prepared according to General Procedure D. After HPLC purification, the title compound **10e** was obtained as a white solid (17 mg, 24%). **^{1}H NMR** (500 MHz, DMSO-*d*$_6$) δ 12.48 (brs, 1H), 10.08 (d, *J*=7.1 Hz, 1H), 8.14–8.04 (m, 2H), 7.94 (d, *J*=8.0 Hz, 1H), 7.63 (d, *J*=8.0 Hz, 1H), 7.15 (d, *J*=38.4 Hz, 2H), 6.75 (t, *J*=5.7 Hz, 1H), 5.18–5.02 (m, 1H), 4.62–4.50 (m, 1H), 4.29–4.21 (m, 2H), 4.21–4.16 (m, 1H), 3.51–3.48 (m, 2H), 2.89–2.77 (m, 3H), 2.72 (dd, *J*=17.0, 8.2 Hz, 1H), 1.83 (s, 3H), 1.73–1.60 (m, 1H), 1.54–1.47 (m, 1H), 1.35 (s, 9H), 1.3–1.27 (m, 2H), 1.28–1.21 (m, 2H), 1.19 (s, 3H), 1.18 (s, 3H), 1.10 (s, 9H); **^{13}C NMR** (126 MHz, DMSO-*d*$_6$) δ 205.3, 172.4, 172.1, 171.9, 169.6, 169.1, 156.0, 77.8, 73.2, 62.2, 58.6, 55.2, 53.6, 49.1, 48.7, 35.5, 29.7, 28.7, 27.7, 23.1, 22.9, 18.4, 18.2; **MS** (ESI) *m/z* 704 [$(M+H)^+$,100%]; **HRMS** (ESI, $[M+H]^+$ calcd for $C_{30}H_{53}N_7O_{10}S$ 704.3647, found 704.3650.

Ac-Ala-Arg[S](Pbf)-Asp-Ala-Ser(*t*Bu)-NH2 (10f)

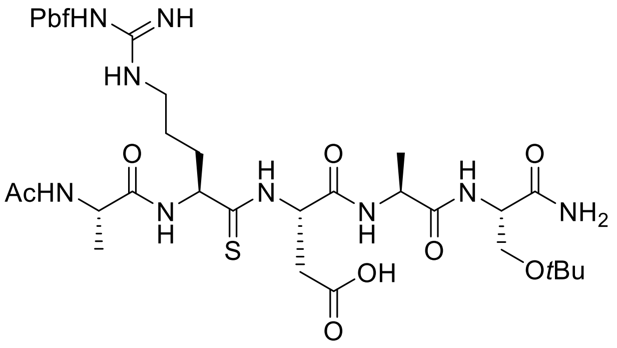

The title compound **10f** was prepared according to General Procedure D. After HPLC purification, the title compound **10f** was obtained as a white solid (16 mg, 18%). **^{1}H NMR** (500 MHz, DMSO-*d*6) δ 12.48 (s, 1H), 10.11 (d, *J*=7.0 Hz, 1H), 8.11–8.07 (m, 2H), 7.94 (d, *J*=8.0 Hz, 1H), 7.63 (d, *J*=8.0 Hz, 1H), 7.19 (s, 1H), 7.11 (s, 1H), 6.61 (s, 1H), 6.53–6.20 (m, 2H), 5.09 (m, 1H), 4.59 (m, 1H), 4.22 (m, 3H), 3.0 (m, 2H), 2.96 (s, 2H), 2.81 (dd, *J*=17.1, 5.3 Hz, 1H), 2.71 (dd, *J*=17.1, 8.2 Hz, 1H), 2.50–2.48 (m, 2H), 2.47 (s, 3H), 2.41 (s, 3H), 2.00 (s, 3H), 1.83 (s, 3H), 1.68 (m, 1H), 1.53 (m, 1H), 1.46–1.38 (m, 8H), 1.19 (s, 3H), 1.18 (s, 3H), 1.10 (s, 9H). **MS** (ESI) *m/z* 884 [(M+H)$^+$,100%]; **HRMS** (ESI, [M+H]$^+$ calcd for $C_{38}H_{62}N_9O_{11}S_2$ 884.4005, found 884.4009.

Ac-Ala-Ala-Asn(Glc(Ac)4)-Ala-Ser(tBu)-NH2 (11a)

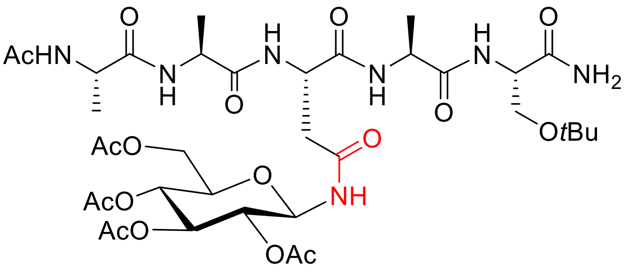

The title compound **11a** was prepared according from thiopeptide **10a** (9 mg, 0.017mmol) to General Procedure E. After HPLC purification, the title compound **11a** was obtained as a white solid (4.2 mg, 29%). **^{1}H NMR** (500 MHz, DMSO-*d*6) δ 8.77 (d, *J*=9.3 Hz, 1H), 8.09 (d, *J*=7.8 Hz, 1H), 8.03–8.00 (m, 2H), 7.78 (d, *J*=7.1 Hz, 1H), 7.73 (d, *J*=8.2 Hz, 1H), 7.14 (brs,1H), 7.07 (brs,1H), 5.36–5.29 (m, 2H), 4.88 (t, *J*=9.8 Hz, 1H), 4.80 (t, *J*=9.4 Hz, 1H), 4.52 (m, 1H), 4.30–4.10 (m, 5H), 4.03 (m, 1H), 3.96 (dd, *J*=12.4, 2.2 Hz, 1H), 3.48–3.45 (m, 2H), 2.62 (m, 1H), 2.45 (m, 1H), 1.99 (s, 3H), 1.98 (s,

3H), 1.94 (s, 3H), 1.92 (s, 3H), 1.82 (d, *J*=2.7 Hz, 3H), 1.21–1.16 (m, 9H), 1.11 (s, 9H). **MS** (ESI) *m/z* 860 [(M+H)$^+$,100%]. **HRMS** (ESI, [M+H]$^+$ calcd. for $C_{36}H_{58}N_7O_{17}$ 860.3884, found 860.3884.

Ac-Ala-Leu-Asn(Glc(Ac)$_4$)-Ala-Ser(*t*Bu)-NH$_2$ (11b)

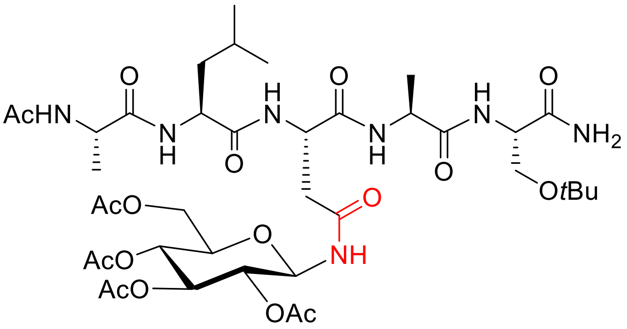

The title compound **11b** was prepared from thiopeptide **10b** (7 mg, 0.012 mmol) according to General Procedure E. After HPLC purification, the title compound **11b** was obtained as a white solid (3.2 mg, 30%). **^{1}H NMR** (500 MHz, DMSO-*d*$_6$) δ 8.77 (d, *J*=9.4 Hz, 1H), 8.12 (d, *J*=7.9 Hz, 1H), 8.01 (d, *J*=7.3 Hz, 1H), 7.89 (d, *J*=7.9 Hz, 1H), 7.76 (d, *J*=7.2 Hz, 1H), 7.74 (d, *J*=8.1 Hz, 1H), 7.13 (s, 1H), 7.08 (s, 1H), 5.36–5.28 (m, 2H), 4.89 (t, *J*=9.8 Hz, 1H), 4.80 (t, *J*=9.4 Hz, 1H), 4.52 (m, 1H), 4.30–4.18 (m, 4H), 4.15 (dd, *J*=12.4, 4.2 Hz, 1H), 4.03 (m, 1H), 3.96 (dd, *J*=12.4, 2.2 Hz, 1H), 3.50–3.46 (m, 4H)2.64 (dd, *J*=16.4, 6.4 Hz, 1H), 2.44 (dd, *J*=16.2, 6.4 Hz, 1H), 1.99 (s, 3H), 1.98 (s, 3H), 1.95 (s, 3H), 1.92 (s, 3H), 1.82 (s, 3H), 1.58 (hept, *J*=6.7 Hz, 1H), 1.47–1.40 (m, 2H), 1.21–1.14 (m, 6H), 1.11 (s, 9H), 0.86 (d, *J*=6.6 Hz, 3H), 0.82 (d, *J*=6.5 Hz, 3H). **MS** (ESI) *m/z* 902 [(M+H)$^+$,100%]. **HRMS** (ESI, [M+H]$^+$ calcd. for $C_{39}H_{64}$N7O_{17} 902.4353, found 902.4350.

Ac-Ala-Phe-Asn(Glc(Ac)$_4$)-Ala-Ser(*t*Bu)-NH$_2$ (11c)

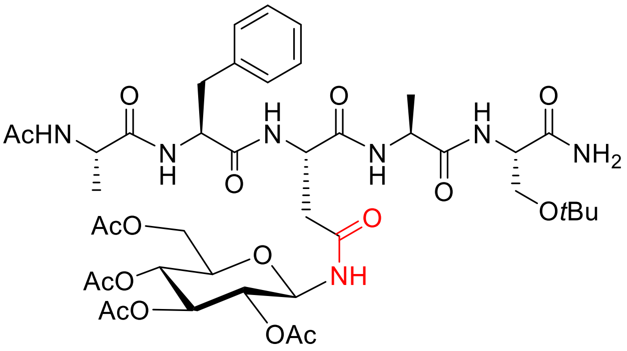

The title compound **11c** was prepared from thiopeptide **10c** (10 mg, 0.016 mmol) according to General Procedure E. After HPLC purification, the title compound **11c** was obtained as a white solid (5 mg, 33%%). **^{1}H NMR** (500 MHz, DMSO-*d*$_6$) δ 8.79 (d, *J*=9.5 Hz, 1H), 8.29 (d, *J*=8.3 Hz, 1H), 7.97 (d, *J*=7.4 Hz, 1H), 7.90 (d, *J*=7.1 Hz, 1H), 7.85 (d, *J*=7.8 Hz, 1H), 7.75 (d, *J*=8.3 Hz, 1H), 7.25–7.15 (m, 5H), 7.13 (s, 1H), 7.08 (s, 1H), 5.35 (t, *J*=9.4 Hz, 1H), 5.31 (t, *J*=9.6 Hz, 1H), 4.89 (t, *J* = 9.8 Hz, 1H), 4.81 (t, *J*=9.4 Hz, 1H), 4.55 (m,1H), 4.43 (m, 1H), 4.27–4.14 (m, 4H), 4.03 (m, 1H), 3.97 (dd, *J*=12.5, 2.2 Hz, 1H), 3.54–3.43 (m, 2H), 3.01 (dd, *J*=14.0, 4.6 Hz, 1H), 2.78 (dd, *J*=13.8, 9.0 Hz, 1H), 2.65 (m, 1H), 2.46–2.43 (m, 1H), 1.98 (d, *J*=3.5 Hz, 6H), 1.94 (s, 3H), 1.92 (s, 3H), 1.79 (s, 3H), 1.20 (d, *J*=7.1 Hz, 3H), 1.14–1.07 (m, 12H). **MS** (ESI) *m/z* 936 [(M+H)$^+$,100%]. **HRMS** (ESI, [M+H]$^+$ calcd. for $C_{42}H_{62}N_7O_{17}$ 936.4197, found 936.4205.

Ac-Ala-Val-Asn-*N*-(Glc(Ac)$_4$)-Ala-Ser(OtBu)-NH$_2$ (11d)

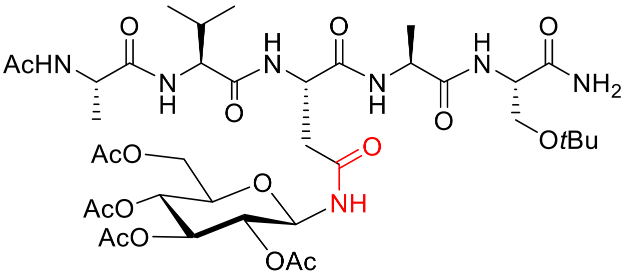

The title compound **11d** was prepared from thiopeptide **10d** (7 mg, 0.012 mmol) according to General Procedure E. After HPLC purification, the title compound **11d** was obtained as a white solid (2.9 mg, 27%). **^{1}H NMR** (500 MHz, DMSO) δ 8.77 (d, *J*=9.4 Hz, 1H), 8.21 (d, *J*=7.8 Hz, 1H), 8.04 (d, *J*=7.5 Hz, 1H), 7.82 (d, *J*=7.2 Hz, 1H), 7.77 (d, *J*=8.2 Hz, 1H), 7.69 (d, *J*=8.5 Hz, 1H), 7.11 (d, *J*=31.7 Hz, 2H), 5.38–5.27 (m, 2H), 4.89 (t, *J*=9.8 Hz, 1H), 4.80 (t, *J*=9.4 Hz, 1H), 4.56 (m, 1H), 4.31 (m, 1H), 4.27–4.18 (m, 2H), 4.18–4.09 (m, 2H), 4.03 (m, 1H), 3.96 (m, 1H), 3.52–3.44 (m, 2H), 2.65 (dd, *J*=16.3, 6.4 Hz, 1H), 2.43 (dd, *J*=16.2, 6.7 Hz, 1H), 1.99 (s, 3H), 1.9 –1.95 (m, 4H), 1.94 (s, 3H), 1.92 (s, 3H), 1.82 (s, 3H), 1.21–1.14 (m, 6H), 1.11 (s, 9H), 0.81 (d, *J*=6.8 Hz, 3H), 0.79 (d, *J*=6.8 Hz, 3H). **MS** (ESI) *m/z* 888 [(M+H)$^+$,100%]. **HRMS** (ESI, [M+H]$^+$ calcd. for $C_{38}H_{62}N_7O_{17}$ 888.4197, found 888.4195.

Ac-Ala-Lys-*N*-(Boc)-Asn-*N*-(Glc(Ac)$_4$)-Ala-Ser(OtBu)-NH$_2$ (11e)

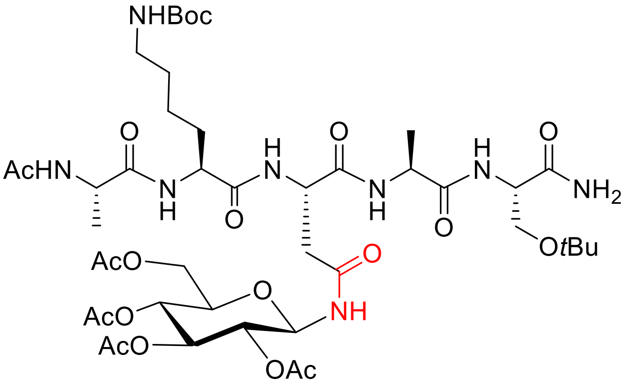

The title compound **11e** was prepared from thiopeptide **10e** (8 mg, 0.017 mmol) according to General Procedure E. After HPLC purification, the title compound **11e** was obtained as a white solid (4.5 mg, 26%). **^{1}H NMR** (500 MHz, DMSO-*d*$_6$) δ 8.77 (d, *J*=9.4 Hz, 1H), 8.13 (d, *J*=7.9 Hz, 1H), 8.02 (d, *J*=7.3 Hz, 1H), 7.88 (d, *J*=7.6 Hz, 1H), 7.81 (d, *J*=7.2 Hz, 1H), 7.73 (d, *J*=8.1 Hz, 1H), 7.12 (s, 1H), 7.08 (s, 1H), 6.71 (t, *J*=5.6 Hz, 1H), 5.24–5.40 (m, 2H), 4.89 (t, *J*=9.8 Hz, 1H), 4.80 (t, *J*=9.5 Hz, 1H), 4.53 (m, 1H), 4.31–4.07 (m, 5H), 4.06–3.99 (m, 1H), 3.96 (dd, *J*=12.4, 2.2 Hz, 1H), 3.52–3.44 (m, 2H), 2.85 (m, 2H), 2.63 (dd, *J*=16.1, 6.5 Hz, 1H), 2.44 (dd, *J*=16.1, 6.5 Hz, 1H), 1.99 (s, 3H), 1.97 (s, 3H), 1.94 (s, 3H), 1.91 (s, 3H), 1.83 (s, 3H), 1.61(m, 1H), 1.47 (m, 1H), 1.36 (s, 9H), 1.35–1.26 (m, 2H), 1.26–1.20 (m, 2H), 1.21–1.13 (m, 6H), 1.11 (s, 9H). **MS** (ESI) *m/z* 1017 [(M+H)$^+$,100%]; **HRMS** (ESI, [M+H]$^+$ calcd. for $C_{44}H_{73}N_8O_{19}$ 1017.4986, found 1017.4988.

Ac-Ala-Arg-*N*-(Pbf)-Asn-*N*-(Glc(Ac)$_4$)-Ala-Ser(OtBu)-NH$_2$ (11f)

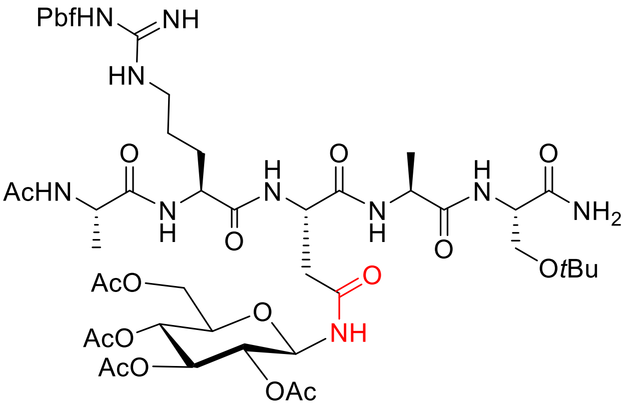

The title compound **11f** was prepared from thiopeptide **10f** (11 mg, 0.012 mmol) according to General Procedure E. After HPLC purification, the title compound **11f** was obtained as a white solid (4.9 mg, 34%). **^{1}H NMR** (500 MHz, DMSO-d_6) δ 8.77 (d, *J*=9.5 Hz, 1H), 8.14 (d, *J*=7.8 Hz, 1H), 8.03 (d, *J*=7.2 Hz, 1H), 7.93 (d, *J*=7.7 Hz, 1H), 7.84 (d, *J*=7.2 Hz, 1H), 7.75 (d, *J*=8.1 Hz, 1H), 7.13 (s,1H), 7.08 (s,1H), 6.94–6.46 (m, 2H), 6.37 (s, 1H), 5.38–5.26 (m, 2H), 4.89 (t, *J*=9.8 Hz, 1H), 4.80 (t, *J*=9.5 Hz, 1H), 4.53 (m, 1H), 4.30–4.10 (m, 5H), 4.02 (m, 1H), 3.96 (dd, *J*=12.5, 2.3 Hz, 1H), 3.00 (q, *J*=6.6 Hz, 2H), 2.96 (s, 2H), 2.62 (dd, *J*=16.2, 6.4 Hz, 1H), 2.49–2.43 (m, 4H), 2.41 (s, 3H), 2.00 (s, 3H), 1.99 (s, 3H), 1.98 (s, 3H), 1.93 (s, 3H), 1.92 (s, 3H), 1.82 (s, 3H), 1.65 (m, 1H), 1.48 (m, 1H), 1.45–1.28 (m, 8H), 1.18 (m, 6H), 1.10 (s, 9H). **MS** (ESI) *m/z* 1197 [(M+H)$^+$,100%]. **HRMS** (ESI, [M+H]$^+$ calcd. for $C_{52}H_{81}N_{10}O_{20}S$ 1197.5344, found 1197.5361.

Ac-Ala-Phe-Asn(GlcNH(Ac)$_4$)-Ala-Ser(*t*Bu)-NH$_2$ (13)

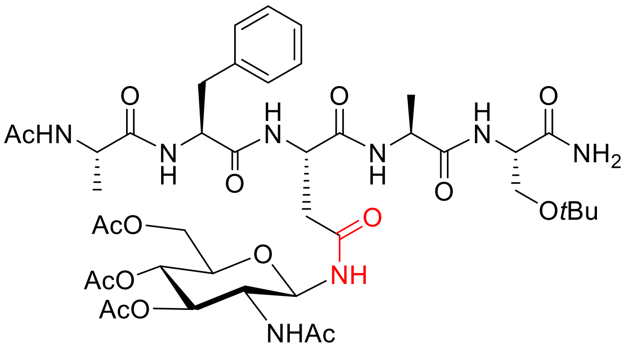

The title compound **13** was prepared from thiopeptide **10c** (11 mg, 0.012 mmol) and amino sugar **12** according to General Procedure E. After HPLC purification, the title compound **3** was obtained as a white solid (4.9 mg, 34%). **^{1}H NMR** (500 MHz, DMSO-d_6) δ 8.65 (d, *J*=9.2 Hz, 1H), 8.33 (d, *J*=7.9 Hz, 1H), 8.16 (d, *J*=8.3 Hz, 1H), 7.96 (d, *J*=6.9 Hz, 1H), 7.92 (d, *J*=9.1 Hz, 1H), 7.88 (d, *J*=7.0 Hz, 1H), 7.75 (d, *J*=8.2 Hz, 1H), 7.25–7.17 (m, 5H), 7.13 (s, 1H), 7.08 (s, 1H), 5.17 (t, *J*=9.5 Hz, 1H), 5.10 (t, *J*=9.9 Hz, 1H), 4.81 (t, *J*=9.8 Hz, 1H), 4.57 (m, 1H), 4.47 (m, 1H), 4.27–4.12 (m, 4H), 3.94 (dd, *J*=12.6, 2.3 Hz, 1H), 3.86 (m, 1H), 3.77 (m, 1H), 3.51–3.44 (m, 2H), 3.04 (dd, *J*=13.8, 3.5 Hz, 1H), 2.76–2.64 (m, 2H), 2.44 (m, 1H), 1.98 (s, 3H), 1.95 (s, 3H), 1.90 (s, 3H), 1.78 (s, 3H), 1.75 (s,

3H), 1.21 (d, *J*=7.1 Hz, 3H), 1.13–1.06 (m, 12H), 0.91 (d, *J*=7.1 Hz, 3H). **MS** (ESI) *m/z* 935 [(M+H)$^+$,100%]; **HRMS** (ESI, [M+H]$^+$ calcd. for $C_{42}H_{63}N_8O_{16}$ 935.4357, found 935.4359.

Ac-Val-Glu-Arg$^{[S]}$(Pbf)-Asp-Gly-Ala-Ser(*t*Bu)-NH$_2$ (14a)

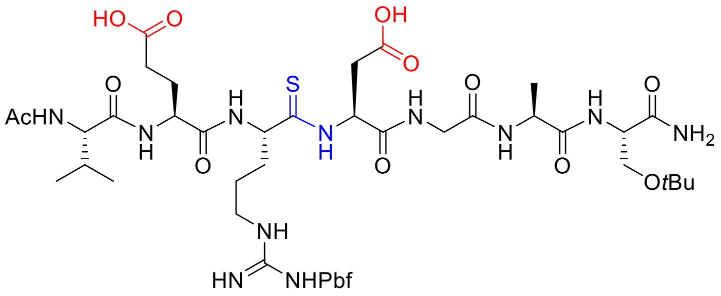

The title compound **14as** was prepared according to General Procedure F (0.05 mmol scale). After HPLC purification, the title compound **14a** was obtained as a white solid (6 mg, 11%). **MS** (ESI) *m/z* 1098 [(M+H)$^+$,100%]. **HRMS** (ESI, [M+H]$^+$) calcd. for $C_{47}H_{76}N_{11}O_{15}S_2$ 1098.4958, found 1098.4958.

Ac-Val-Glu-Arg$^{[S]}$(Pbf)-Asp-Phe-Ala-Ser(*t*Bu)-NH$_2$ (14b)

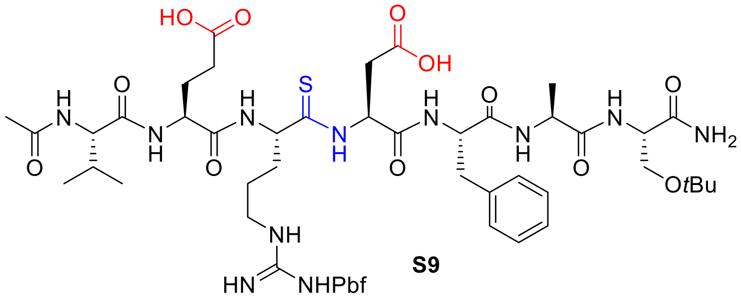

S9

The title compound **14b** was prepared according to General Procedure F on (0.05 mmol) scale. After HPLC purification, the title compound **14b** was obtained as a white solid (7 mg, 13%). **MS** (ESI) *m/z* 1188 [(M+H)$^+$,100%]. **HRMS** (ESI, [M+H]$^+$) calcd. for $C_{54}H_{82}N_{11}O_{15}S_2$ 1188.5428, found 1188.5428.

Ac-Val-Glu-Arg[S](Pbf)-Asp-Gly-His-Ser(tBu)-NH2 (14d)

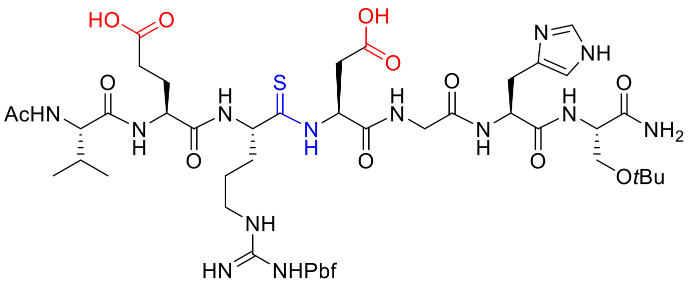

The title compound **14d** was prepared according to General Procedure F on (0.05 mmol) scale. After HPLC purification, the title compound **14d** was obtained as a white solid (7 mg, 12%). **MS** (ESI) *m/z* 1164 [$(M+H)^+$,100%]. **HRMS** (ESI, $[M+H]^+$) calcd. for $C_{50}H_{78}N_{13}O_{15}S_2$ 1164.5176, found 1164.5175.

Ac-Val-Glu-Arg(Pbf)-Asn(Glc(Ac)4)-Gly-Ala-Ser(*t*Bu)-NH2 (15a)

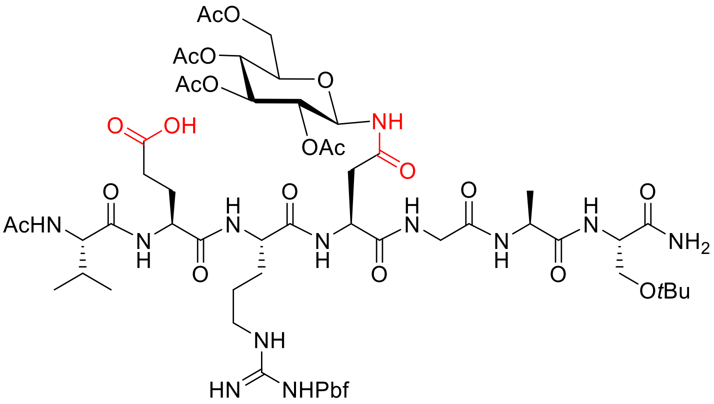

The title compound **15a** was prepared from thiopeptide **14a** (5 mg, 0.005 mmol) according to General Procedure G. After HPLC purification, the title compound **15a** was obtained as a white solid (1.1 mg, 16%). **MS** (ESI) *m/z* 1411 [$(M+H)^+$,100%]. **HRMS** (ESI, $[M+H]^+$) calcd. for $C_{61}H_{95}N_{12}O_{24}S$ 1411.6297, found 1411.6297.

Ac-Val-Glu-Arg(Pbf)-Asn(Glc(Ac)4)-Phe-Ala-Ser(*t*Bu)-NH2 (15b)

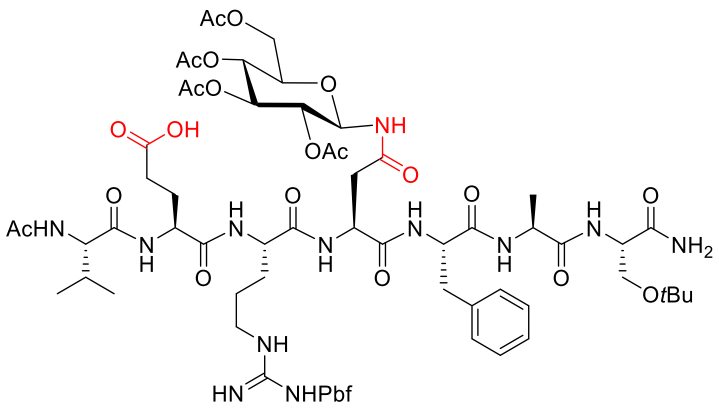

The title compound **15b** was prepared from thiopeptide **14b** (6 mg, 0.005 mmol) according to General Procedure G. After HPLC purification, the title compound **15b** was obtained as a white solid (1.5 mg, 29%). **MS** (ESI) *m/z* 1501 [$(M+H)^+$,100%]. **HRMS** (ESI, $[M+H]^+$) calcd. for $C_{68}H_{101}N_{12}O_{24}S$ 1501.6767, found 1411.6767.

Ac-Val-Glu-Arg(Pbf)-Asn(Glc(Ac)4)-Gly-His-Ser(tBu)-NH2 (15d)

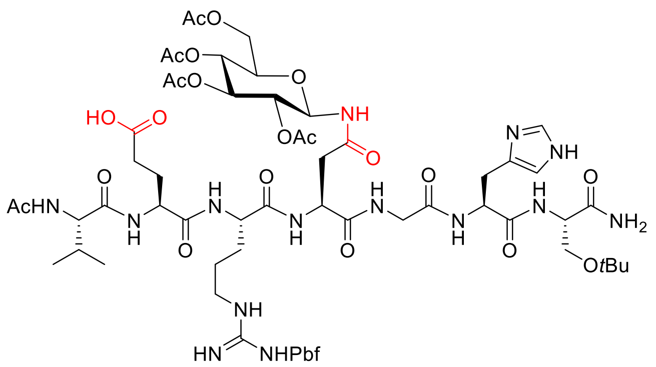

The title compound **15d** was prepared from thiopeptide **14d** (6 mg, 0.005 mmol) and aminosugar **4** (12.5 mg, 0.036 mmol) according to General Procedure G. After HPLC purification, the title compound **15d** was obtained as a white solid (2.9 mg, 39%). **MS** (ESI) *m/z* 1477 [$(M+H)^+$,100%]. **HRMS** (ESI, $[M+H]^+$) calcd. for $C_{64}H_{97}N_{14}O_{24}S$ 1477.6515, found 1477.6512.

Fig S1: ^{1}H NMR of **S2** (400 MHz, $CDCl_3$)

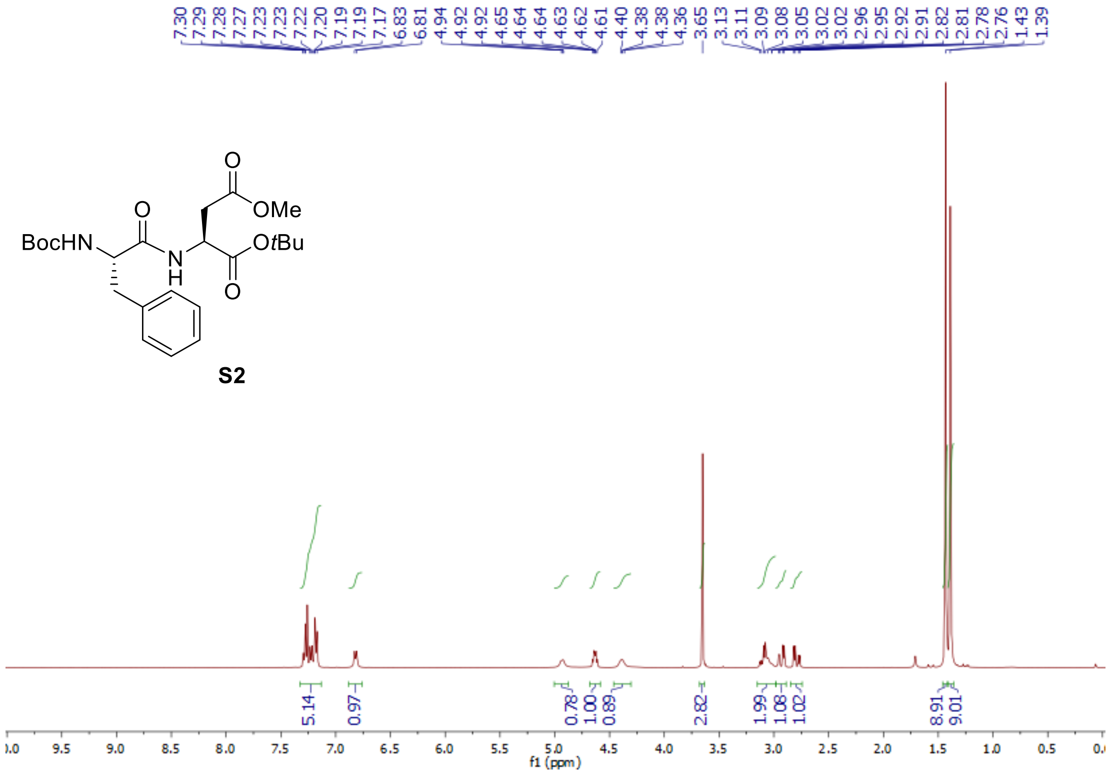

Fig S2: ^{13}C NMR of **S2** (100 MHz, $CDCl_3$)

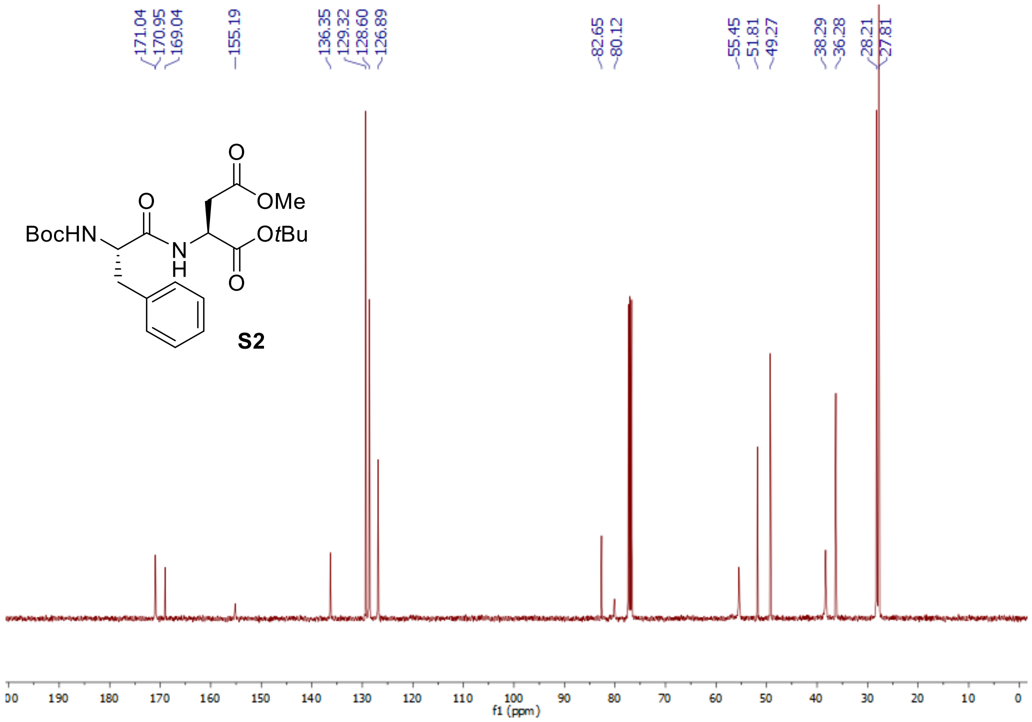

Fig S3: ^{1}H NMR of **S3** (400 MHz, $CDCl_3$)

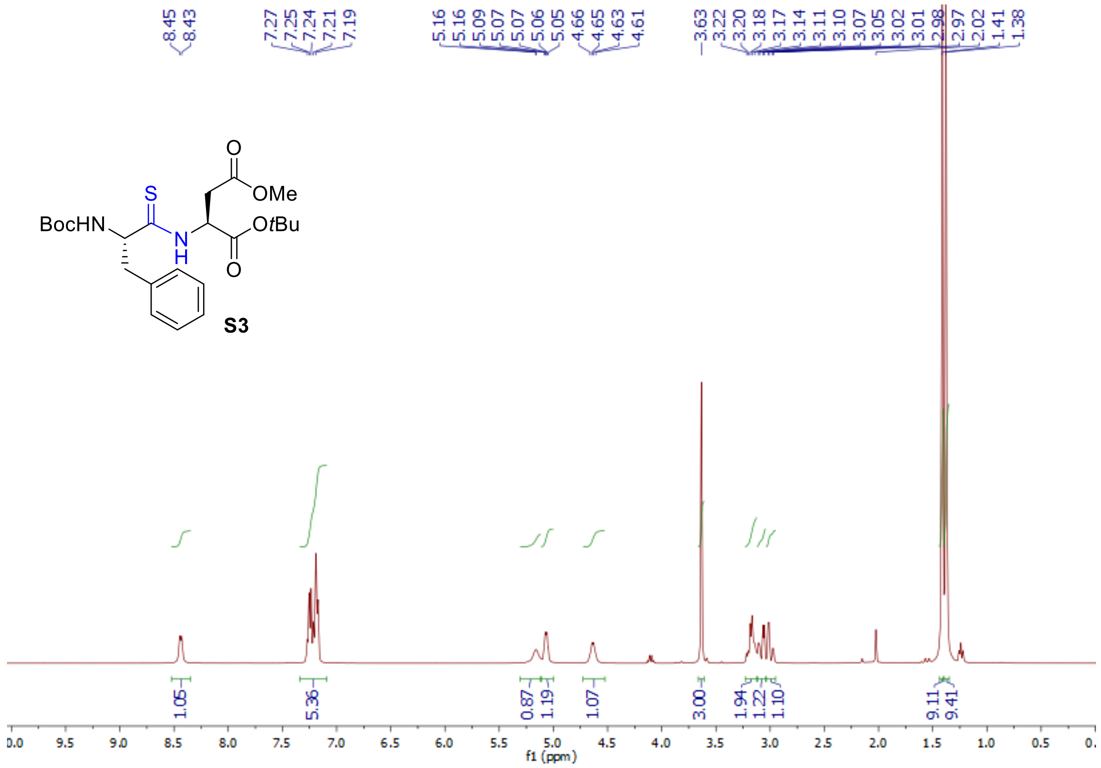

Fig S4: ^{13}C NMR of **S3** (100 MHz, $CDCl_3$)

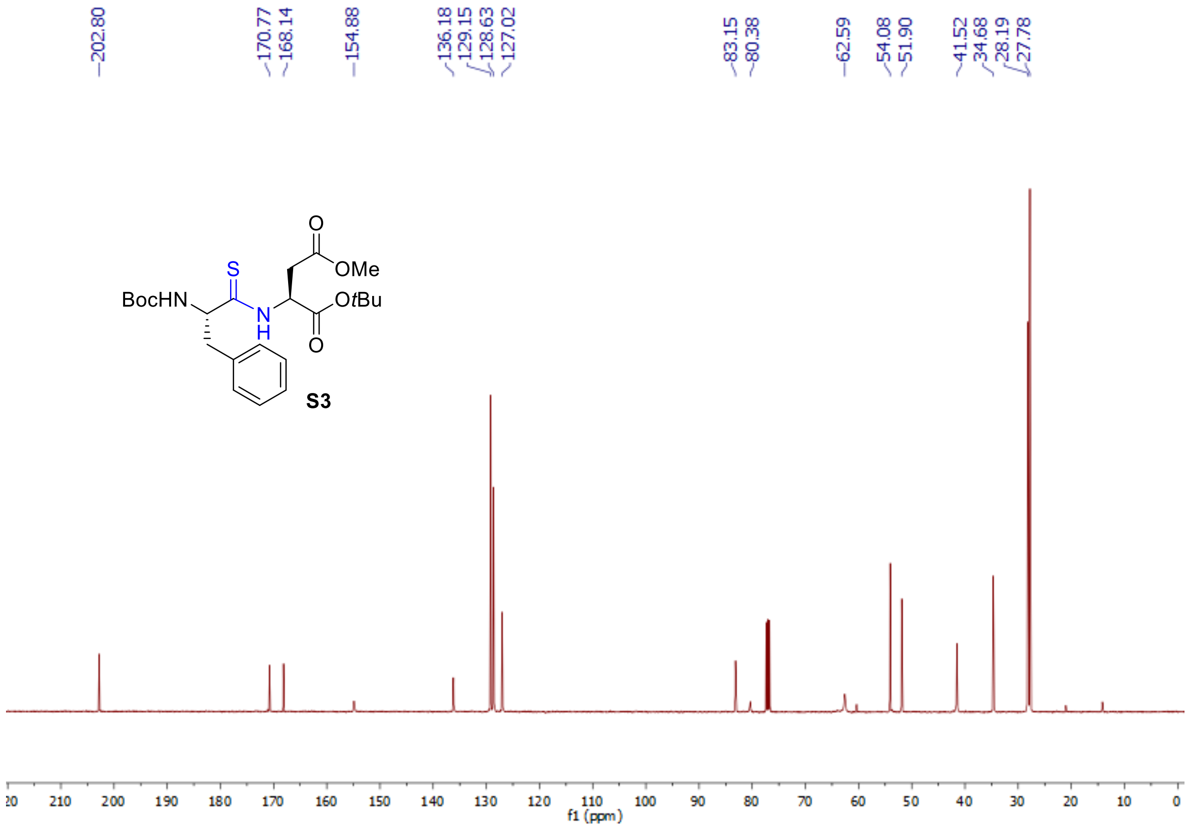

Fig S5: ^{1}H NMR of **5** (400 MHz, $CDCl_3$)

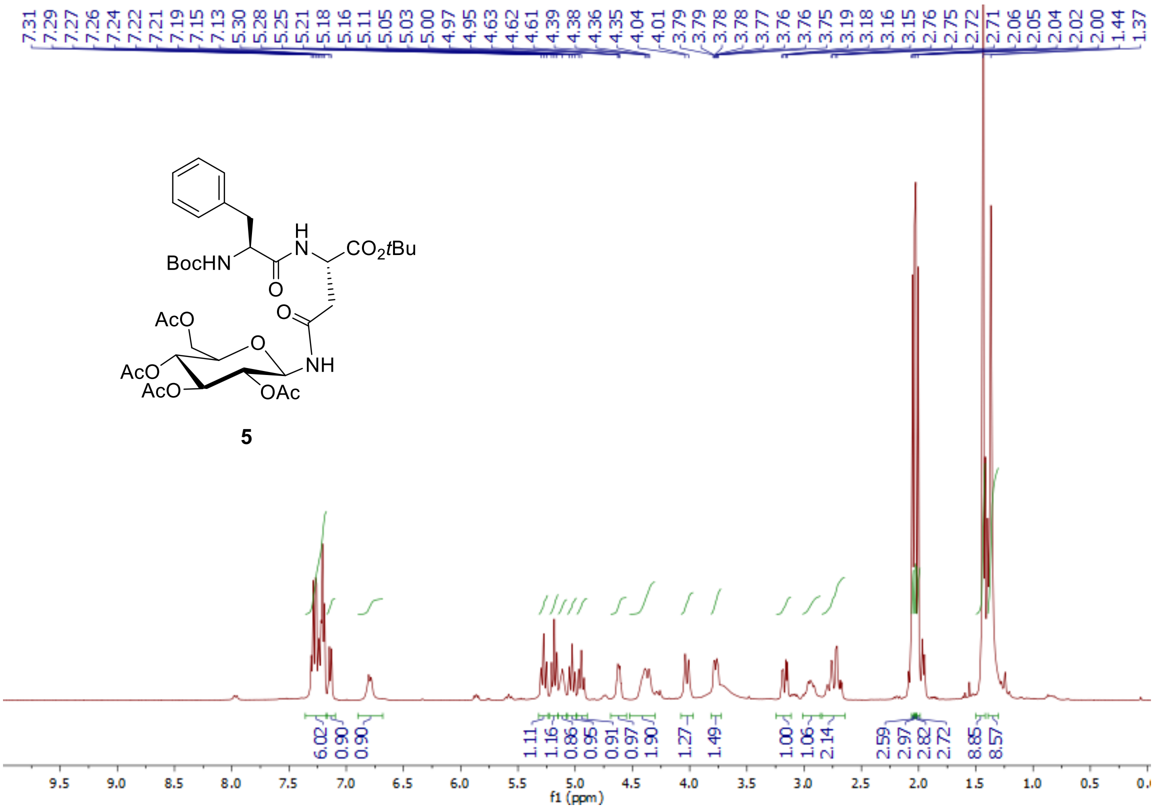

Fig S6: ^{13}C NMR of **5** (100 MHz, $CDCl_3$)

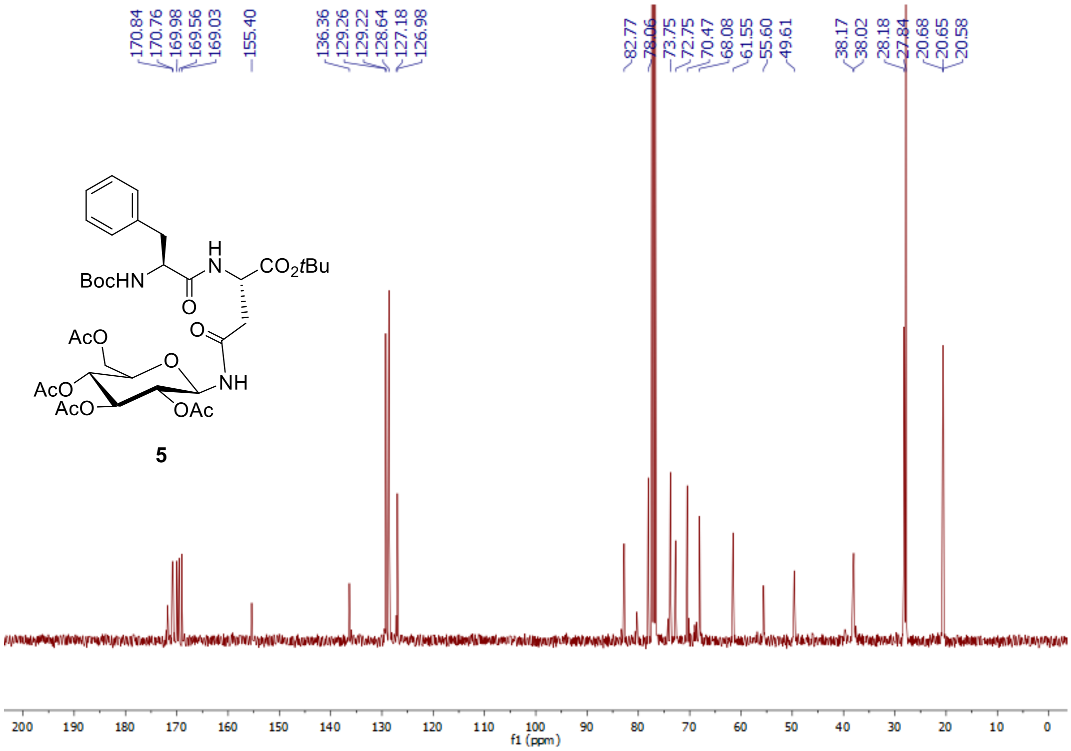

Fig S7: ^{1}H NMR of **S6a** (400 MHz, DMSO-d$_6$)

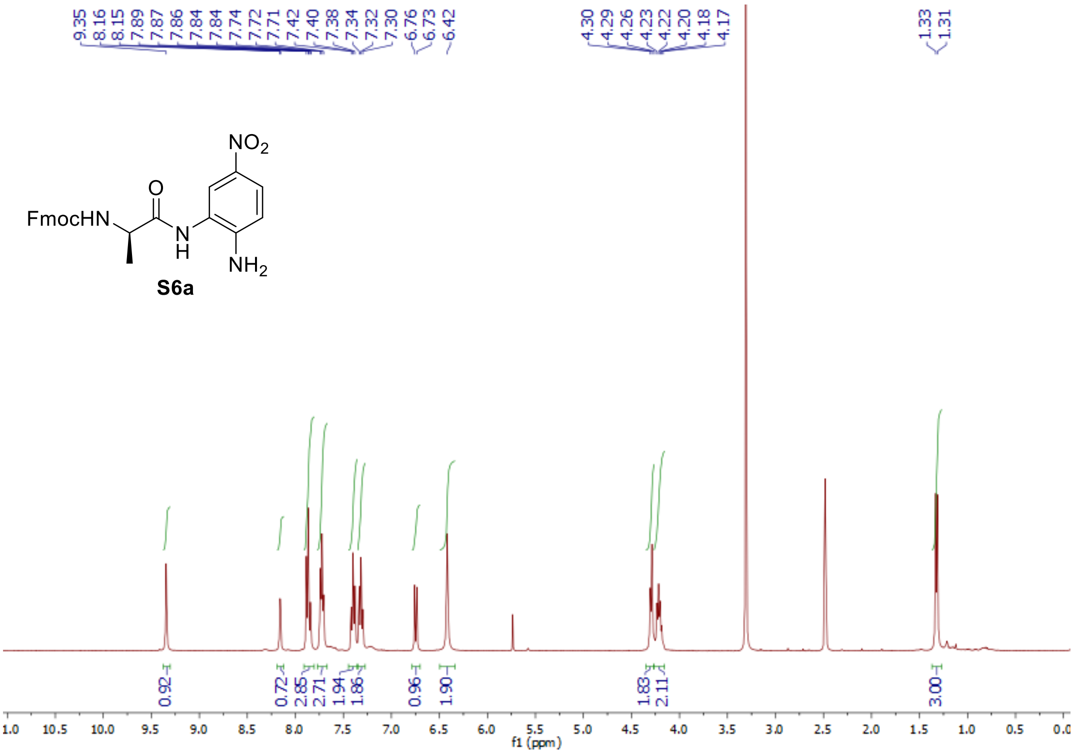

Fig S8: ^{13}C NMR of **S6a** (100 MHz, DMSO-d$_6$)

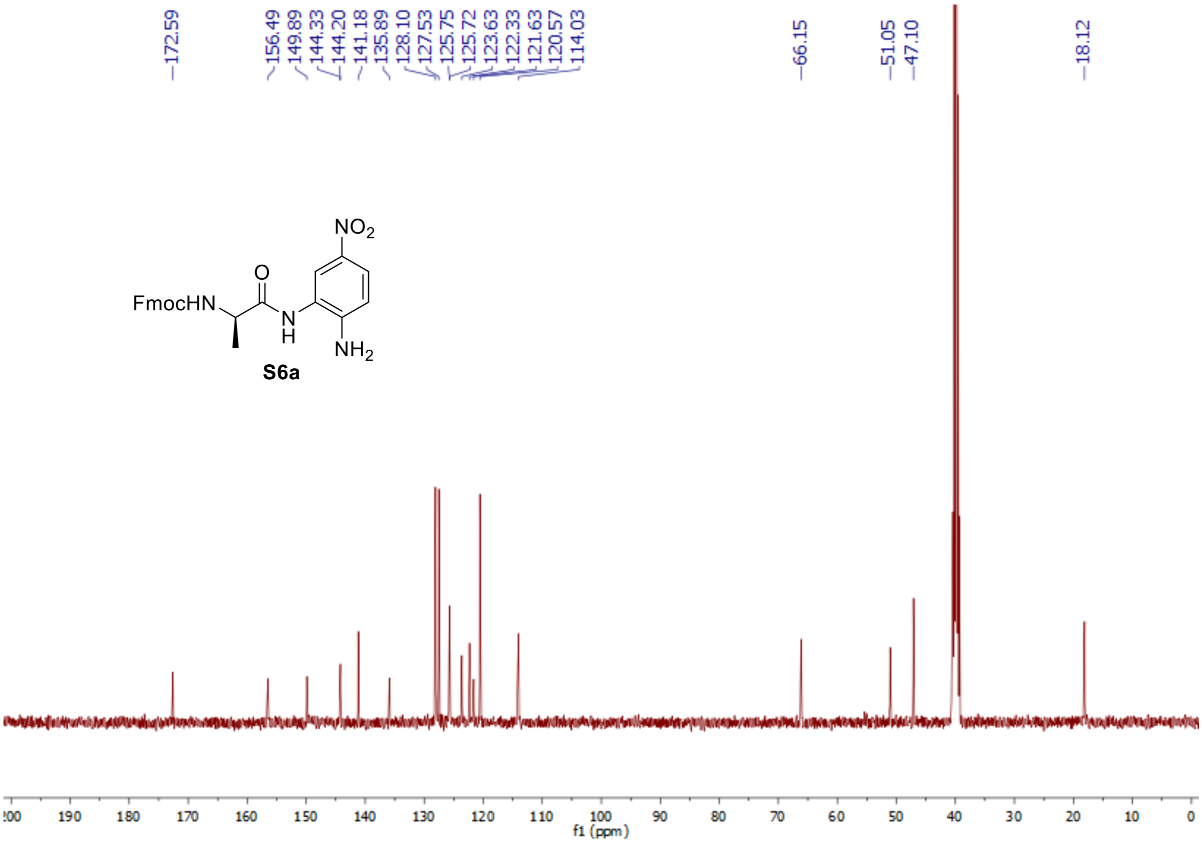

Fig S9: ^{1}H NMR of **S6b** (400 MHz, DMSO-d$_6$)

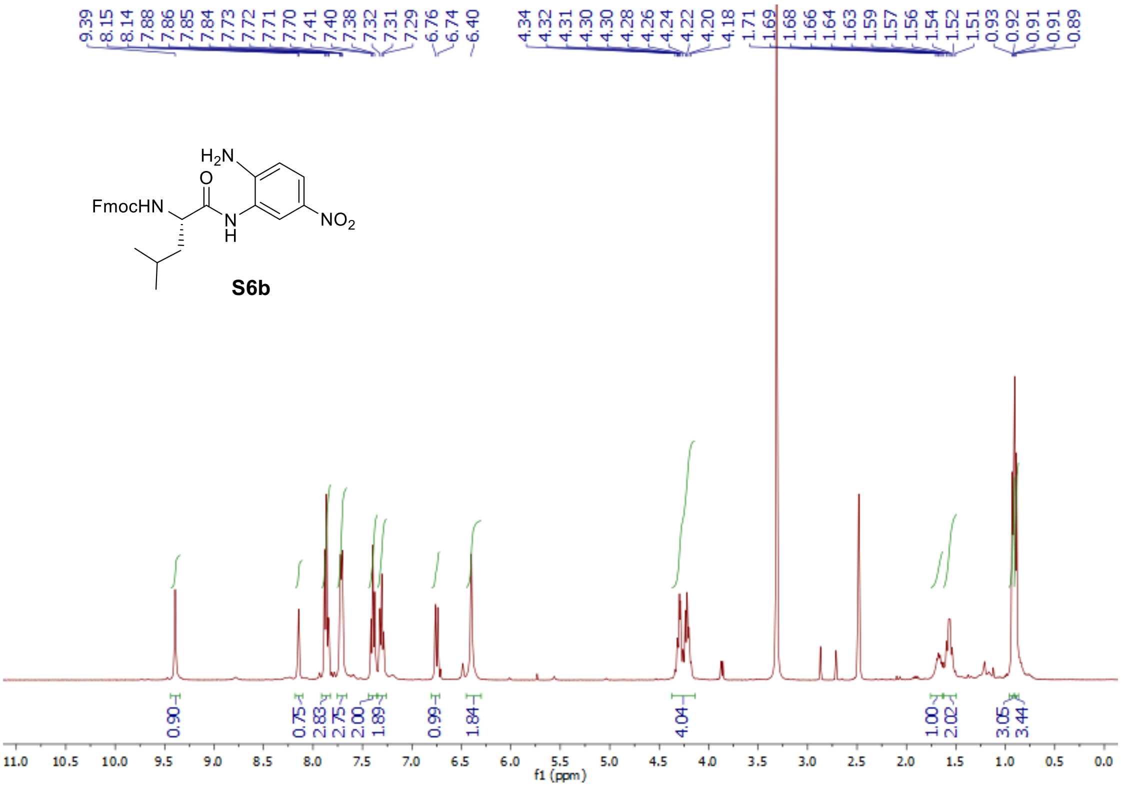

Fig S10: ^{13}C NMR of **S6b** (100 MHz, DMSO-d$_6$)

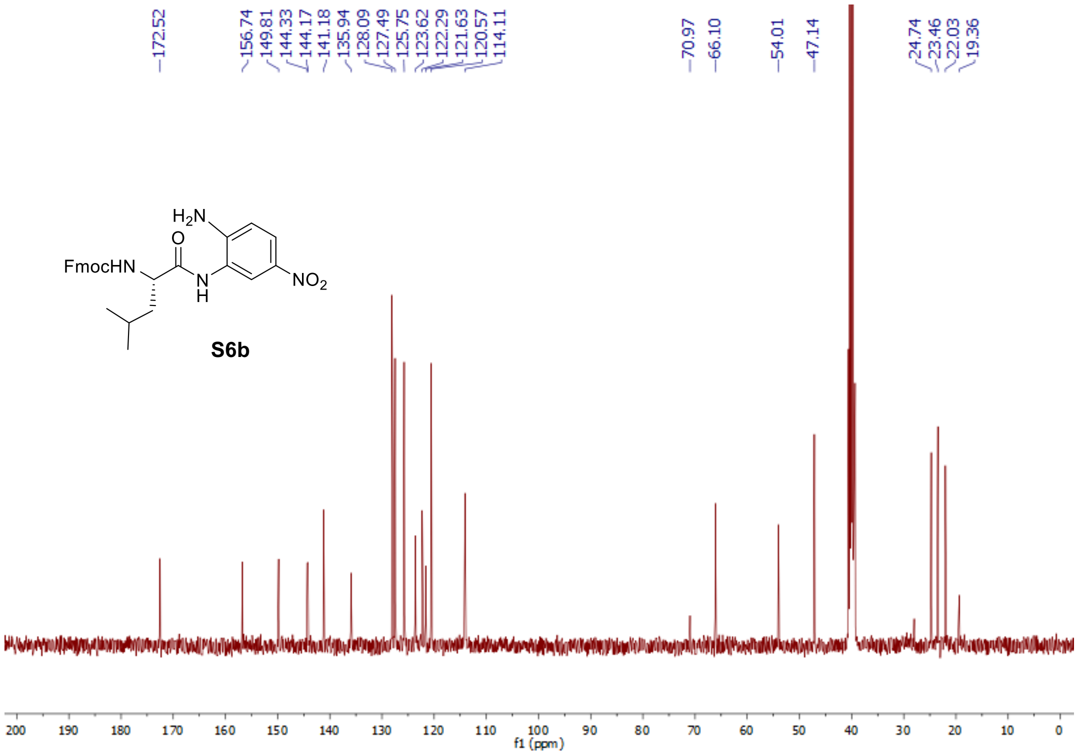

Fig S11: ^{1}H NMR of **S6c** (400 MHz, DMSO-d_6)

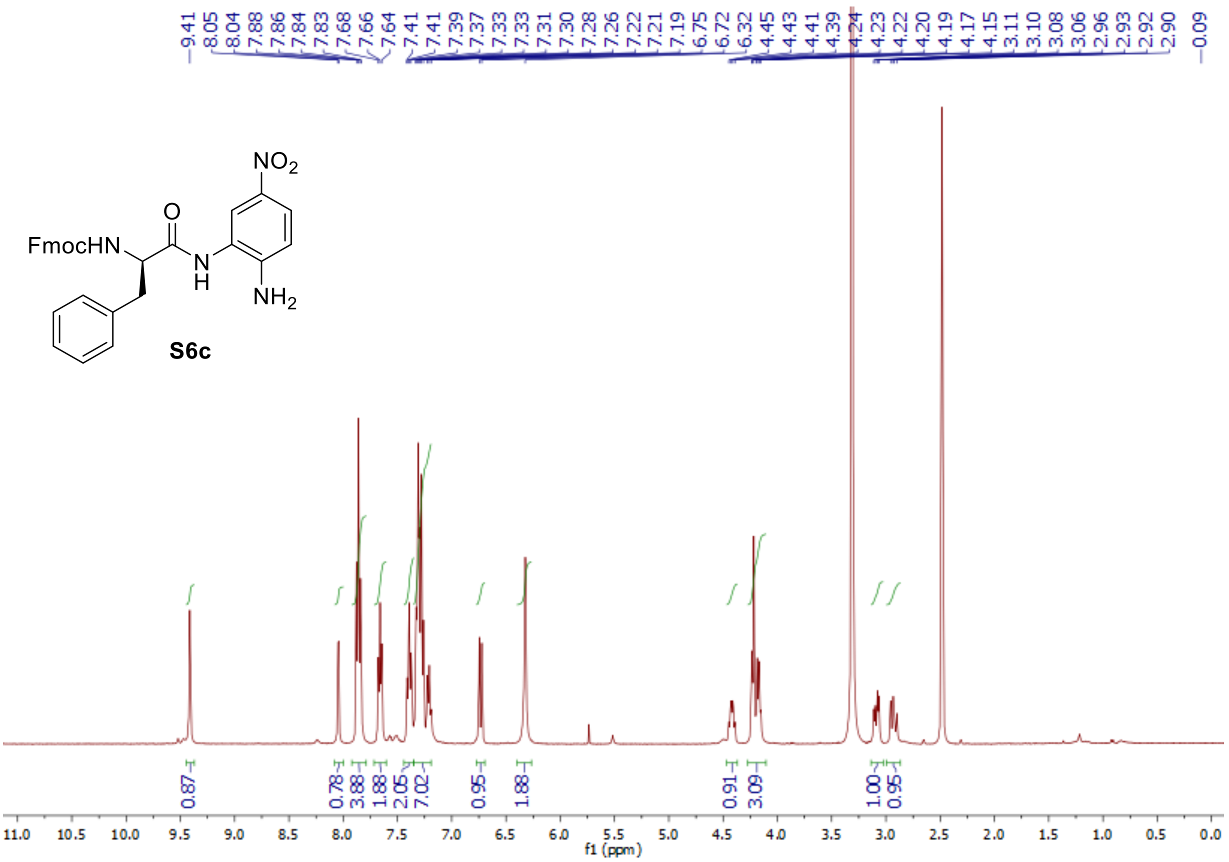

Fig S12: ^{13}C NMR of **S6c** (100 MHz, DMSO-d_6)

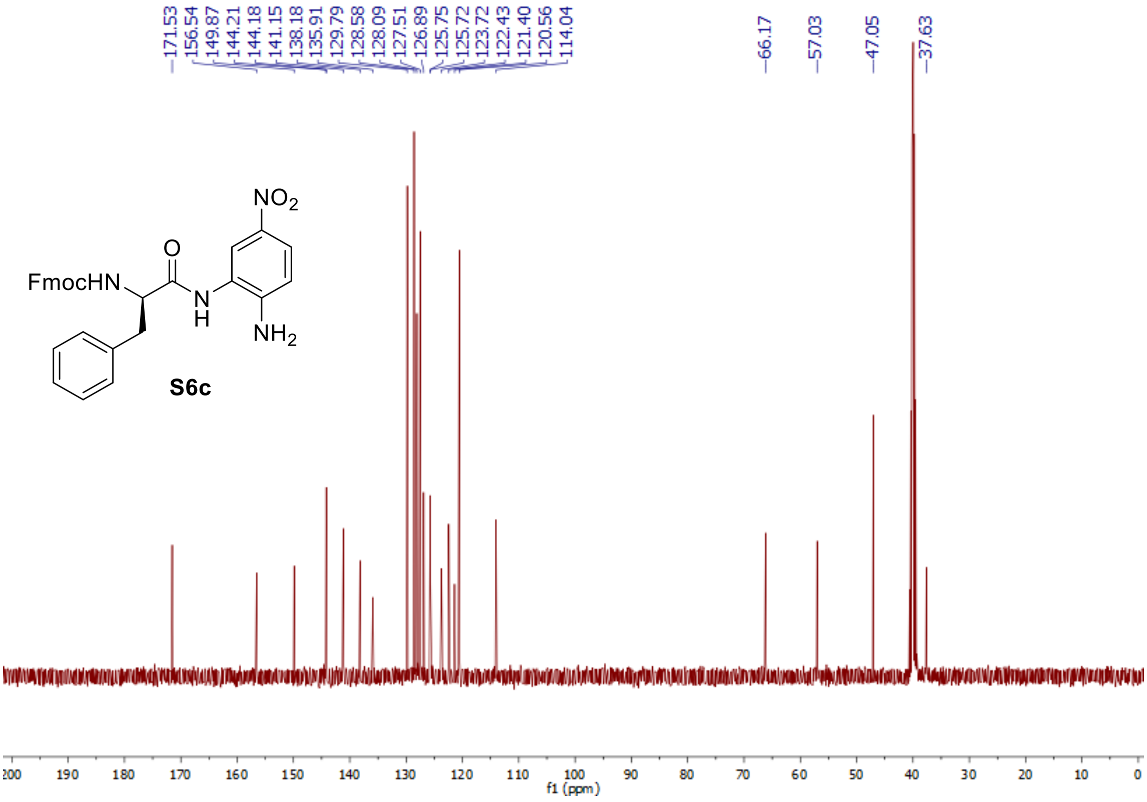

Fig S13: ^{1}H NMR of **S6d** (400 MHz, DMSO-d_6)

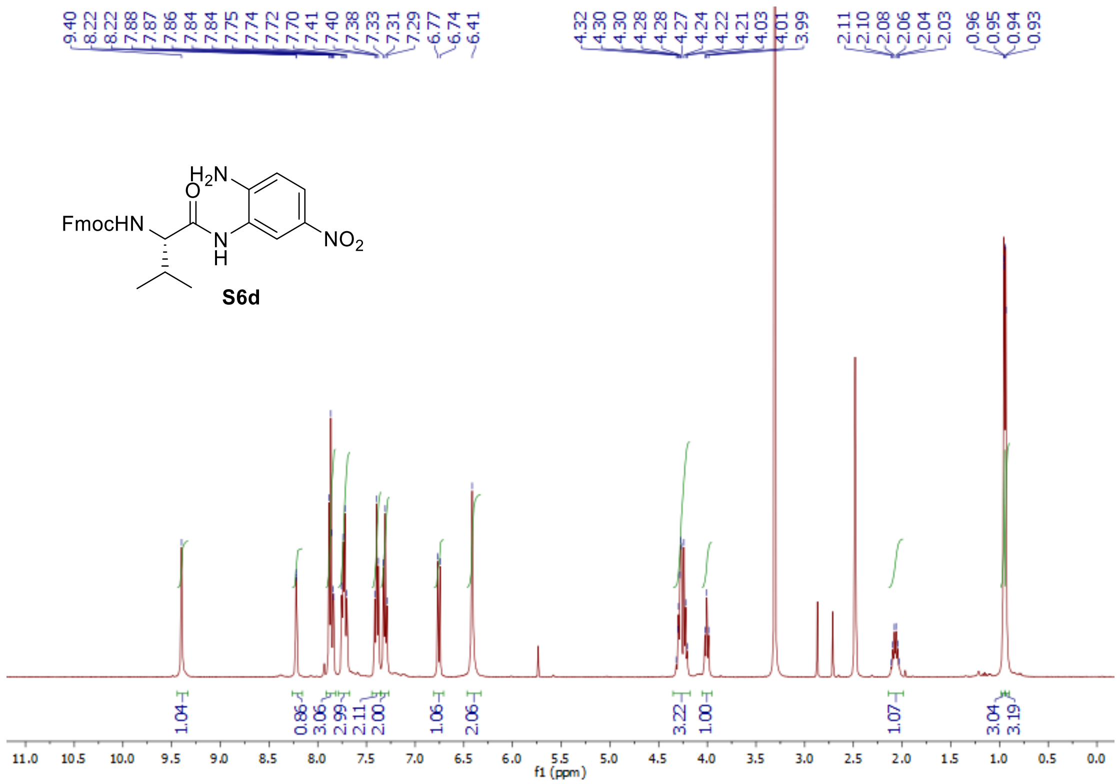

Fig S14: ^{13}C NMR of **S6d** (100 MHz, DMSO-d_6)

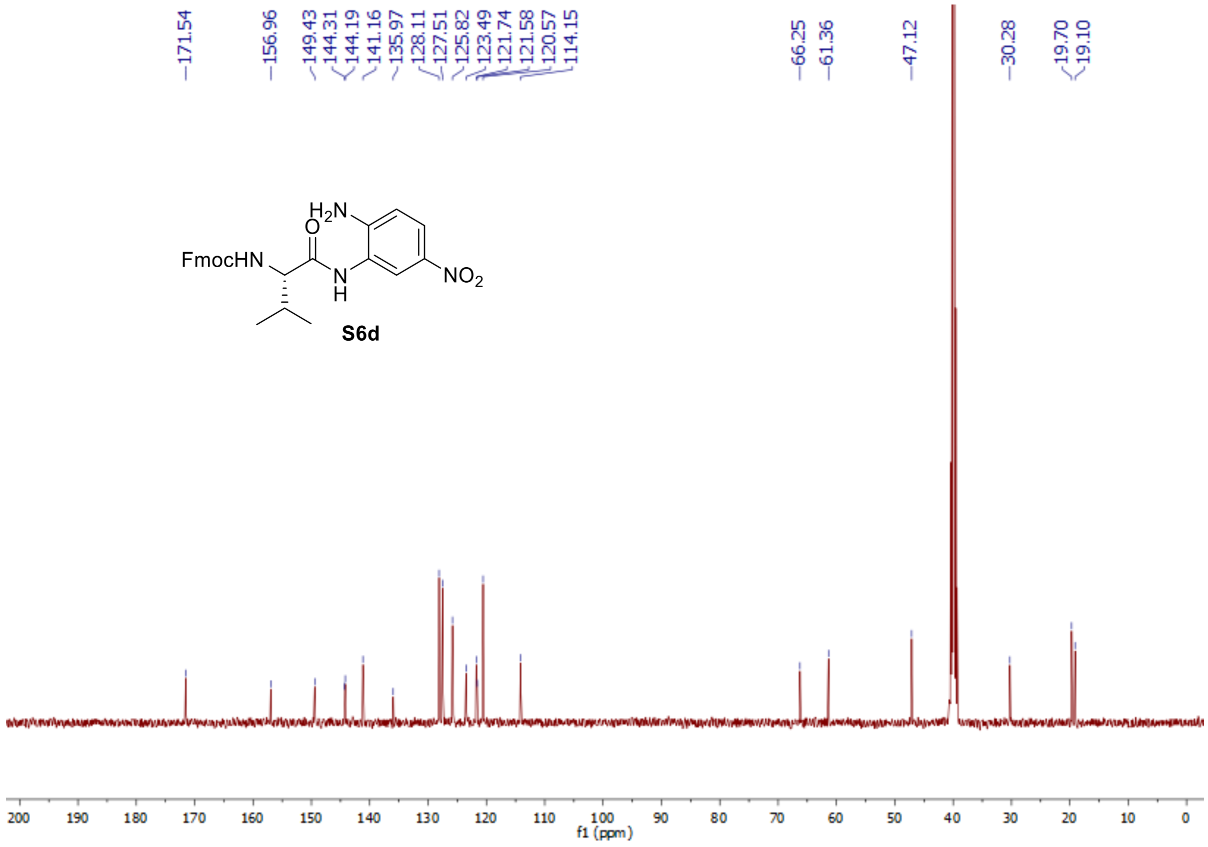

Fig S15: ^{1}H NMR of **S6e** (400 MHz, DMSO-d$_6$)

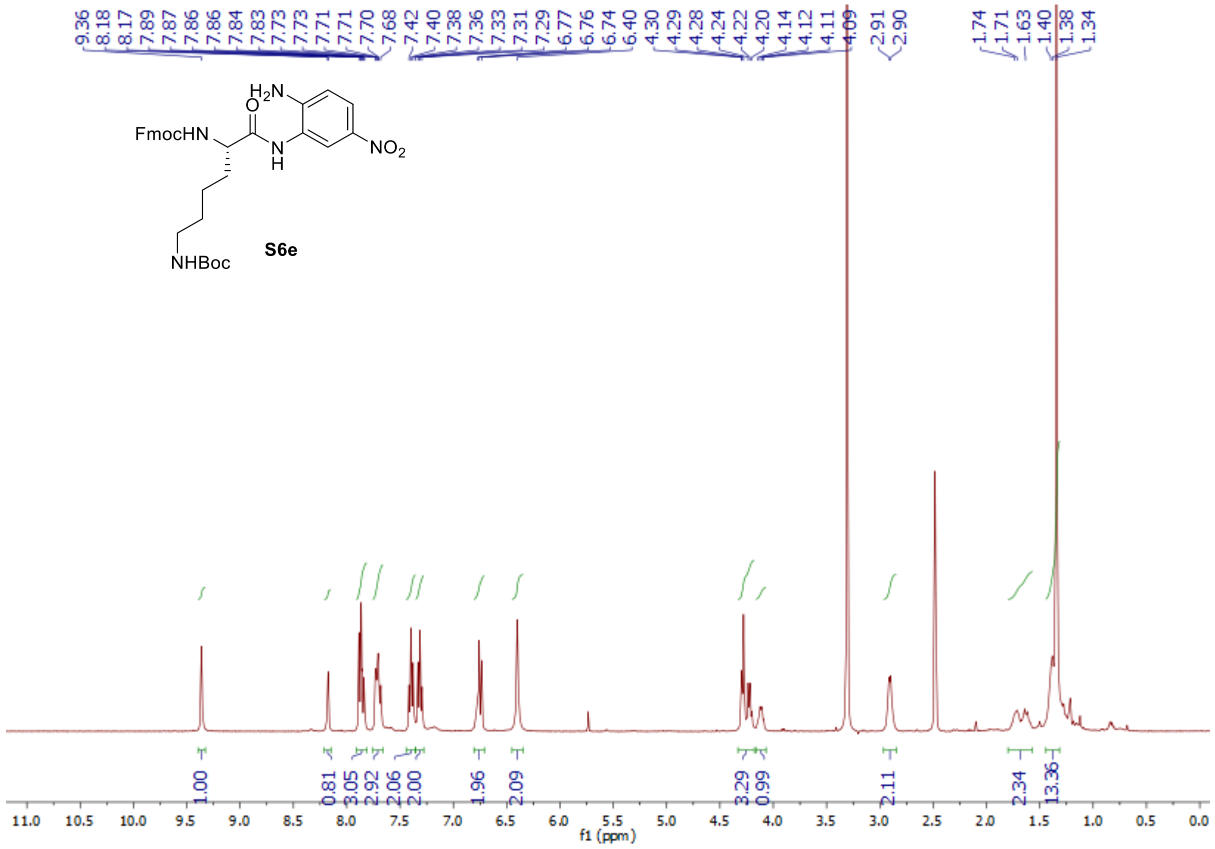

Fig S16: ^{13}C NMR of **S6e** (100 MHz, DMSO-d$_6$)

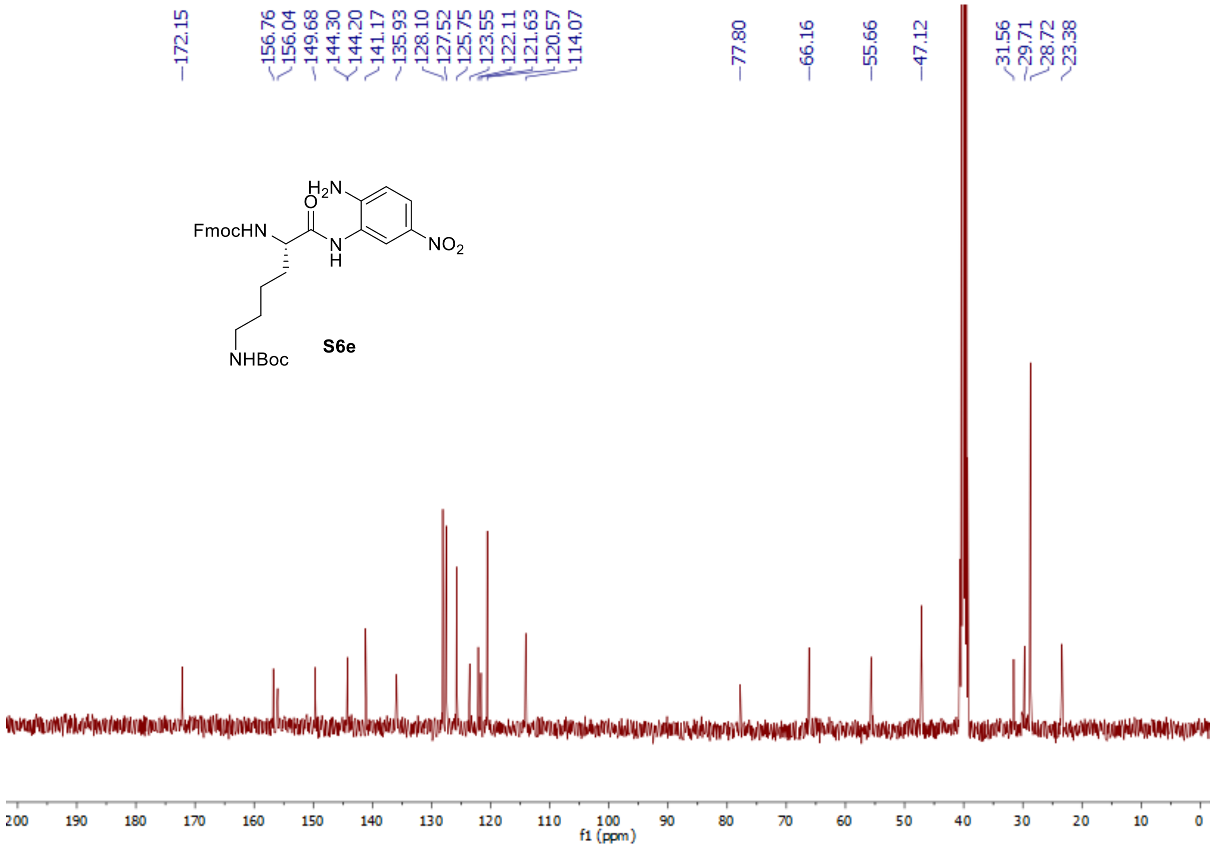

Fig S17: ^{1}H NMR of **S6f** (400 MHz, DMSO-d_6)

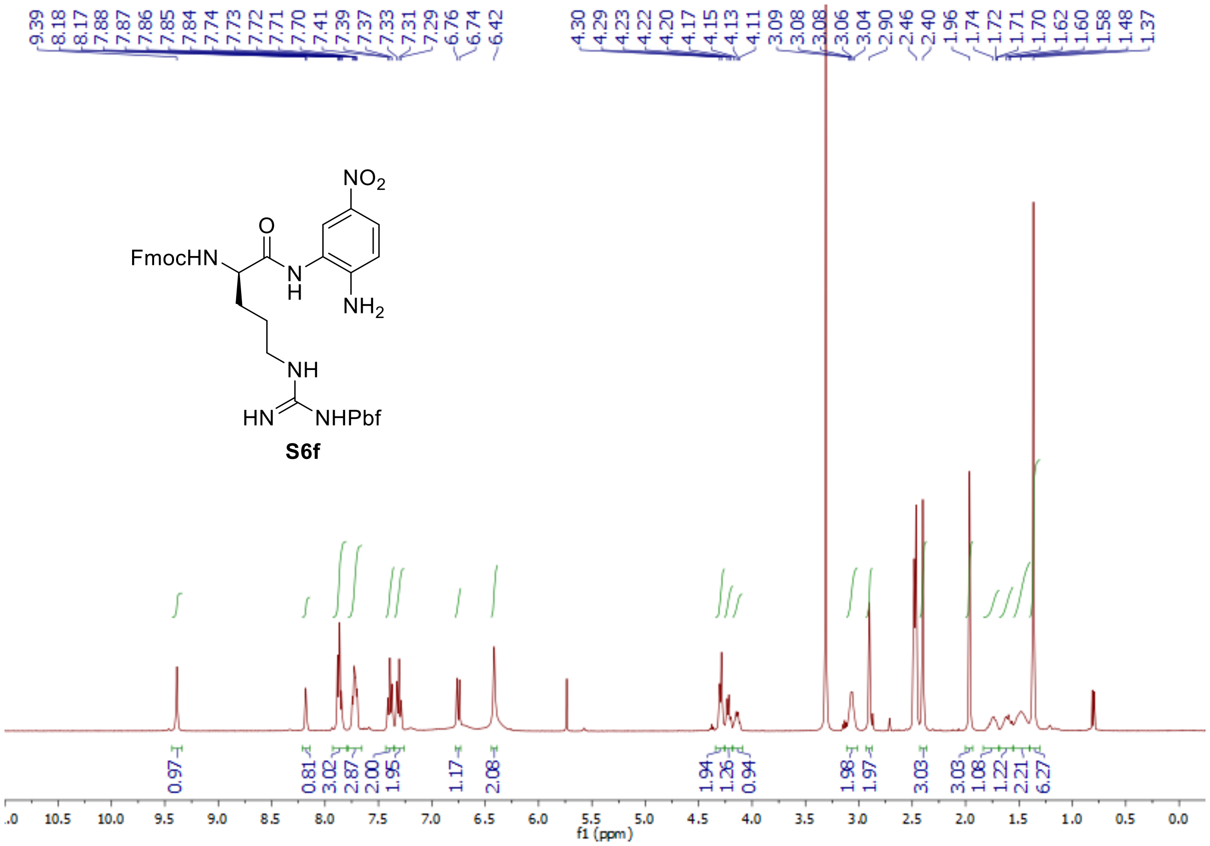

Fig S18: ^{13}C NMR of **S6f** (100 MHz, DMSO-d_6)

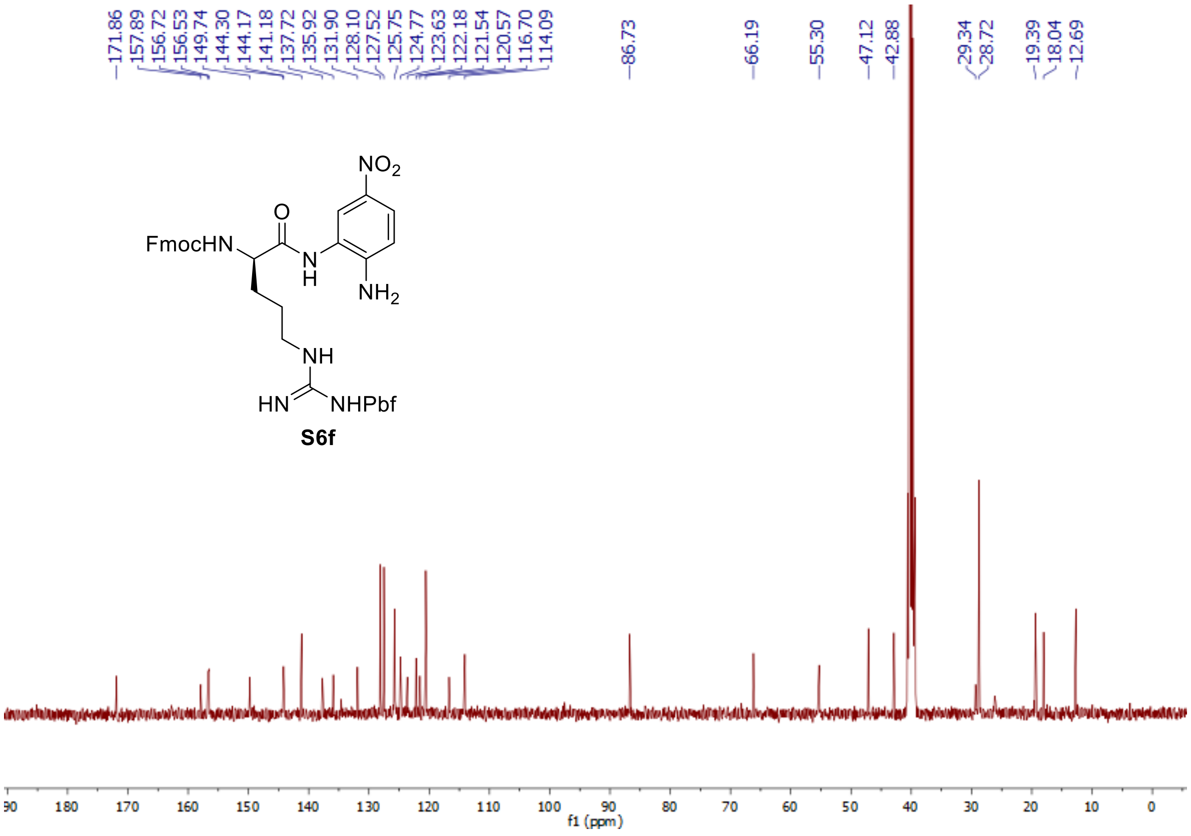

Fig S19: ^{1}H NMR of **S7a** (400 MHz, DMSO-d_6)

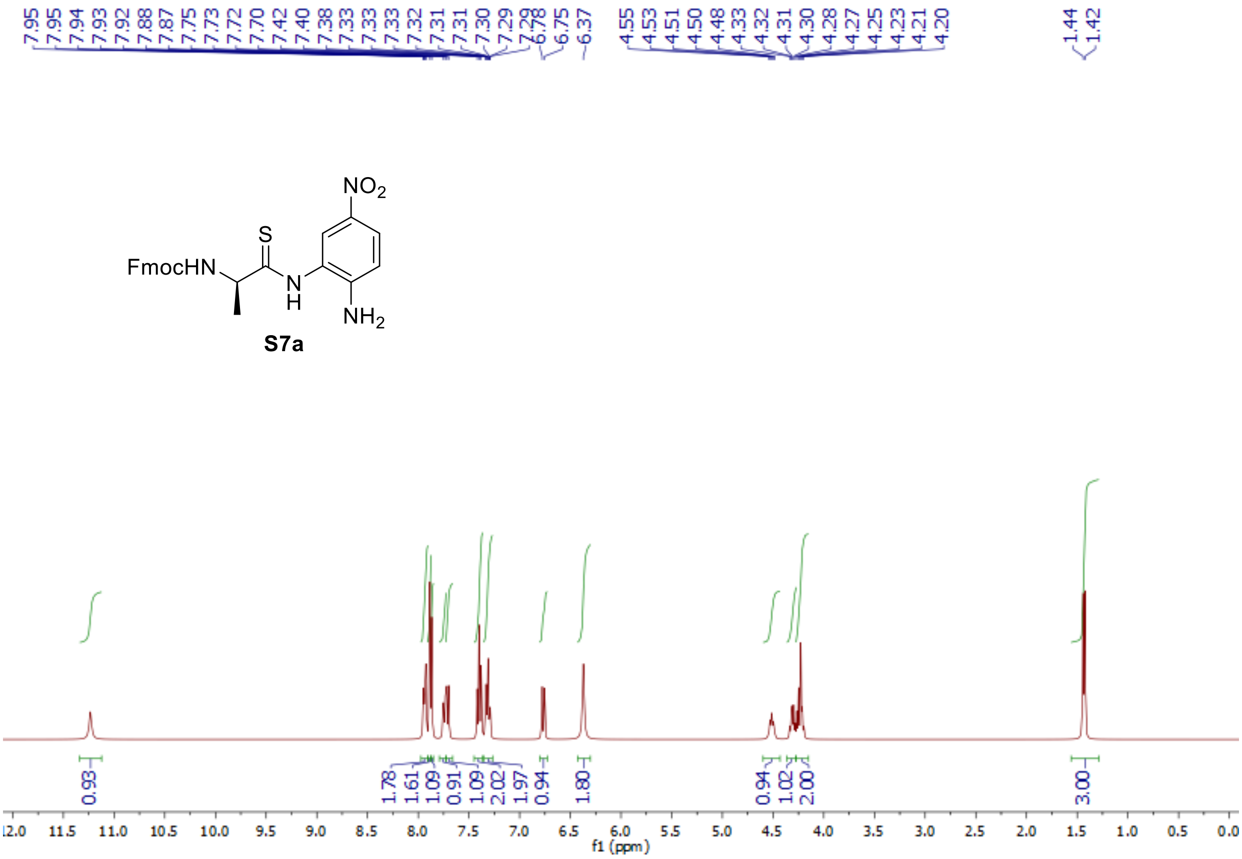

Fig S20: ^{1}H NMR of **S7b** (400 MHz, DMSO-d_6)

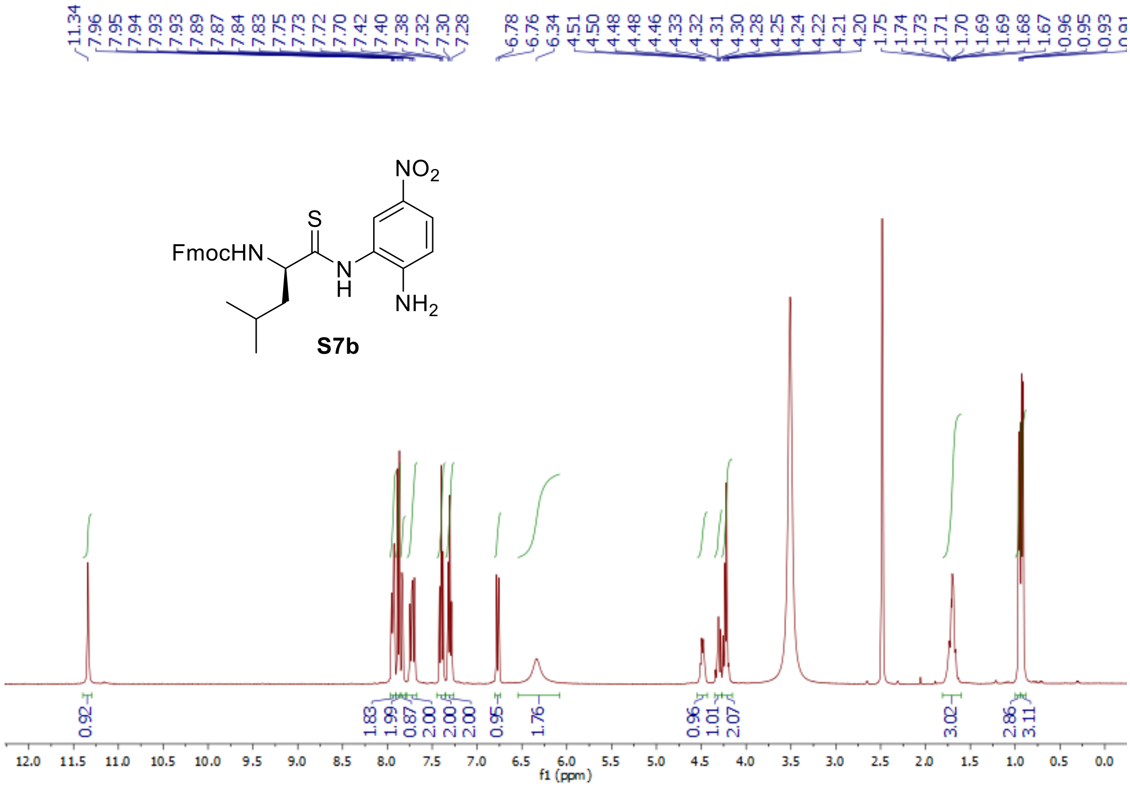

Fig S21: ^{1}H NMR of **S7c** (400 MHz, DMSO-d$_6$)

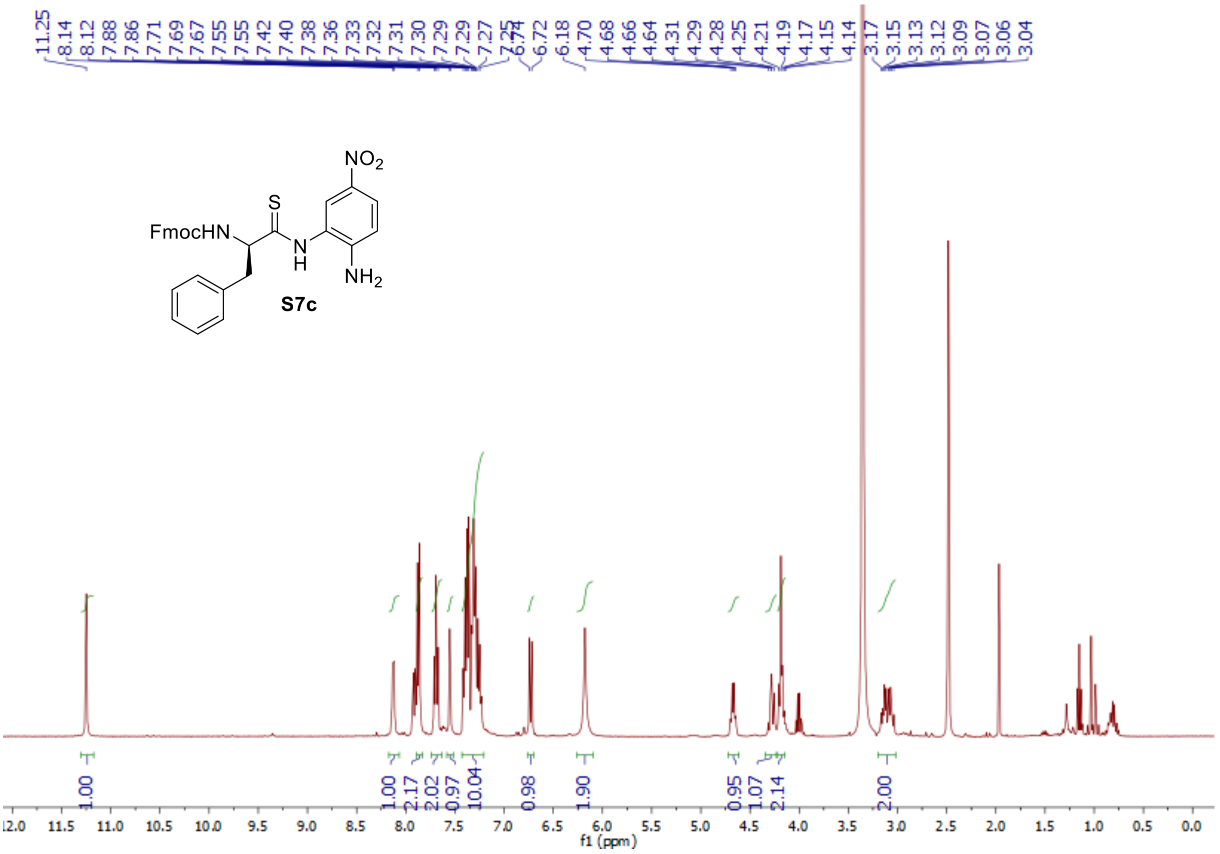

Fig S22: ^{1}H NMR of **S7d** (400 MHz, DMSO-d$_6$)

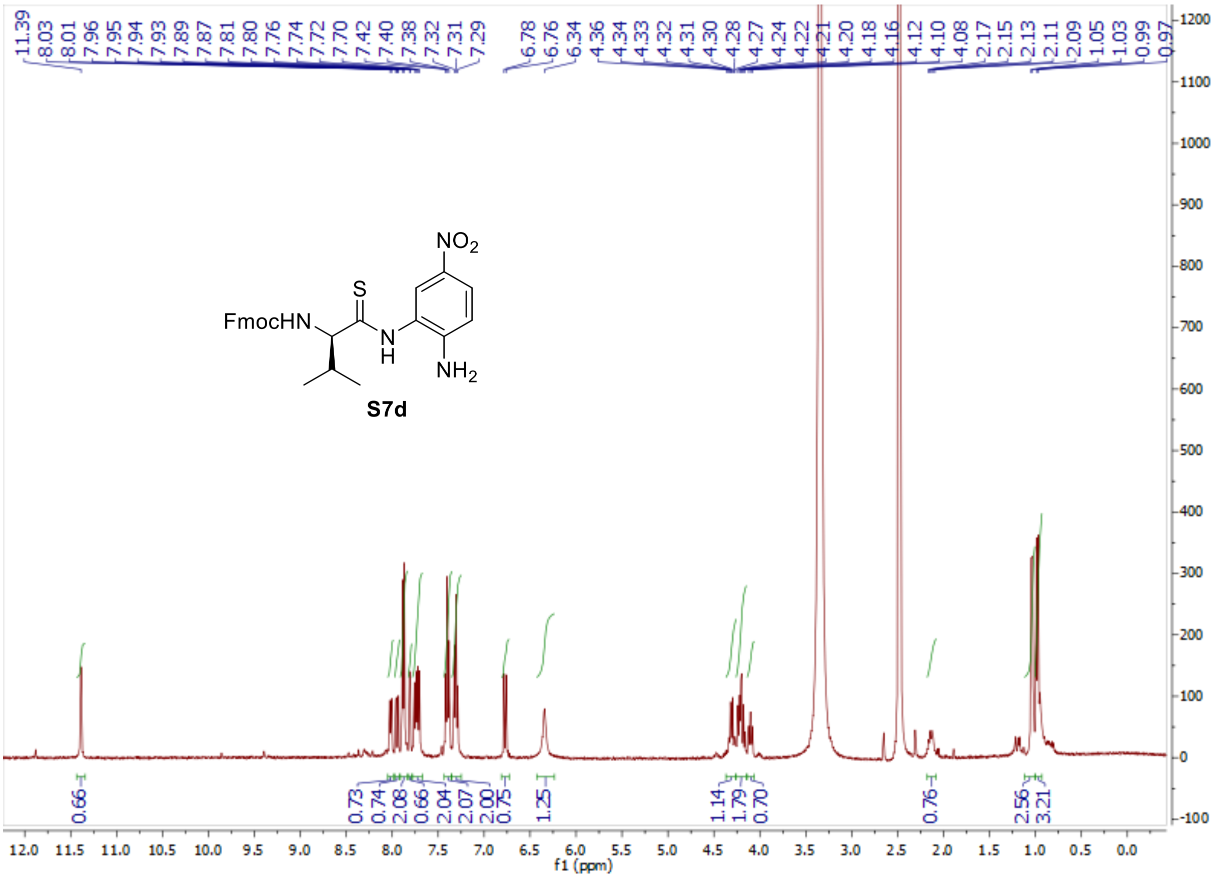

Fig S23: ^{1}H NMR of **S7e** (400 MHz, DMSO-d$_6$)

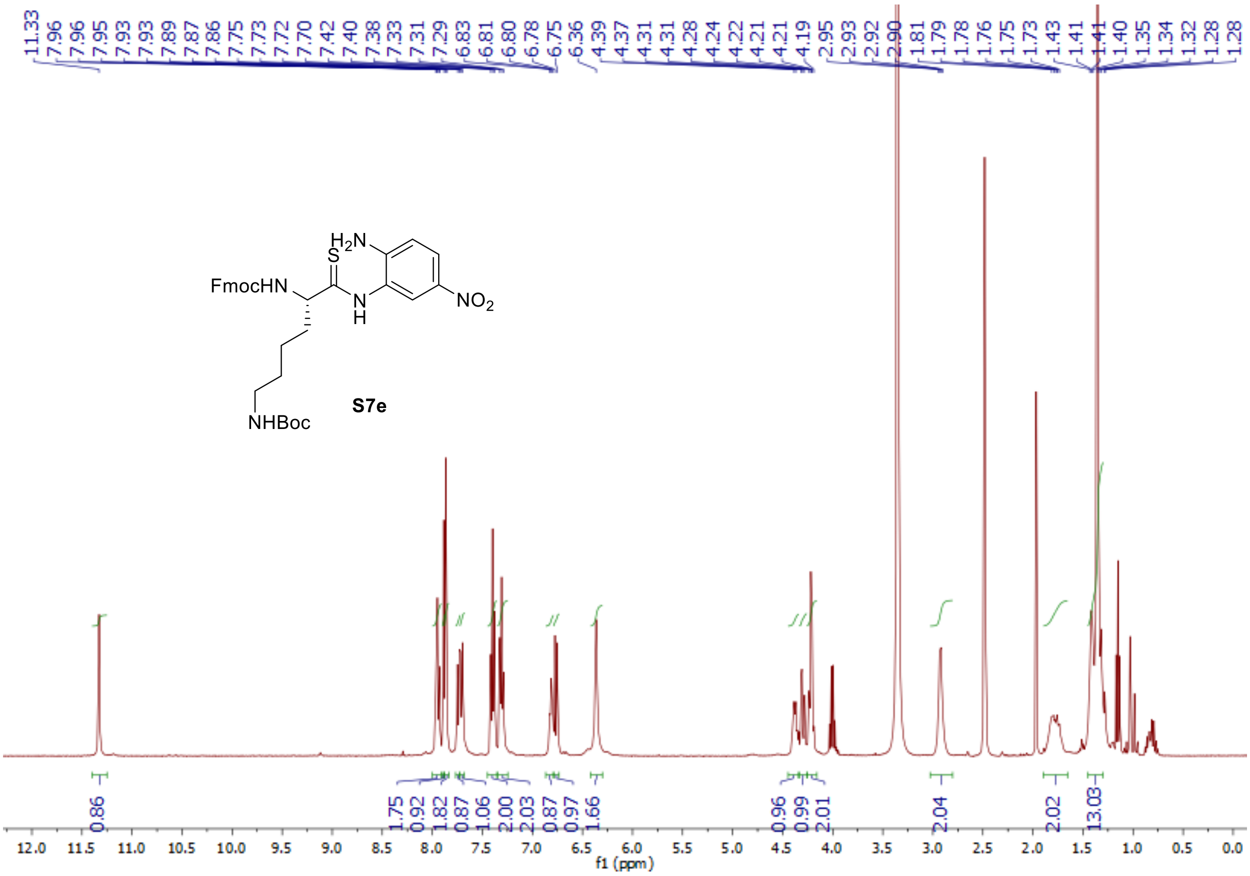

Fig S24: ^{1}H NMR of **S7f** (400 MHz, DMSO-d$_6$)

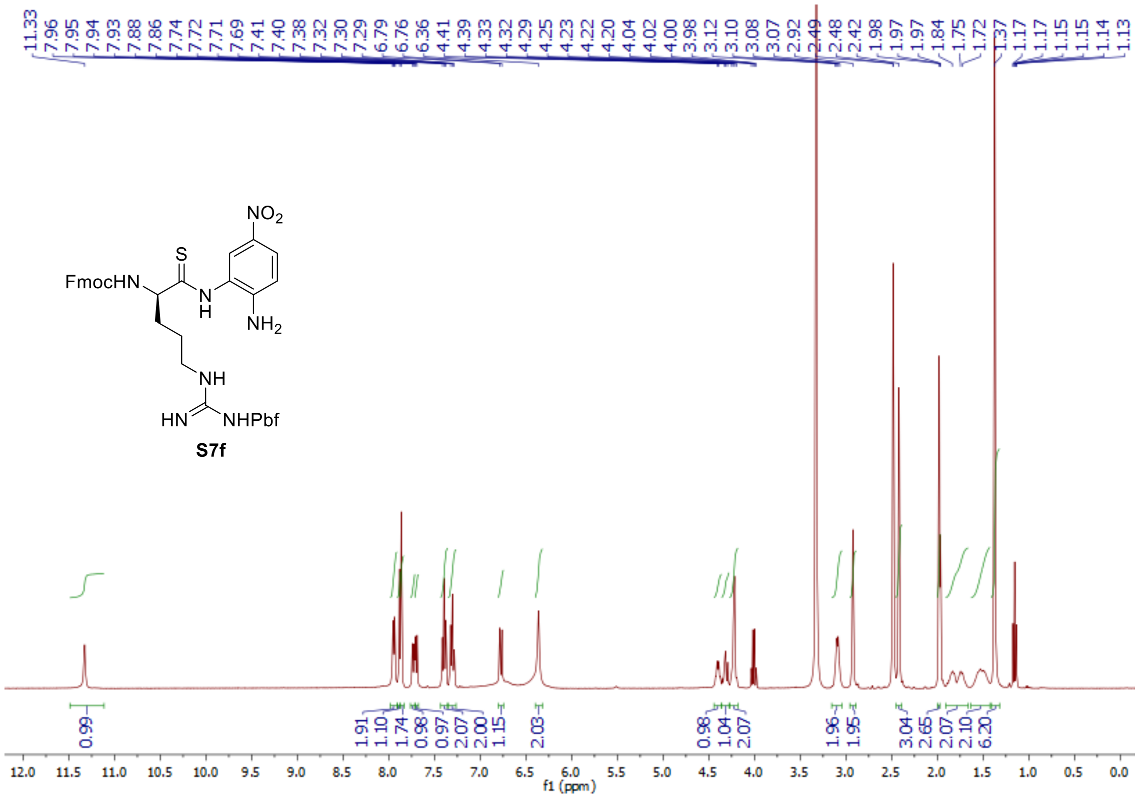

Fig S25: ^{1}H NMR of **10a** (500 MHz, DMSO-d$_6$)

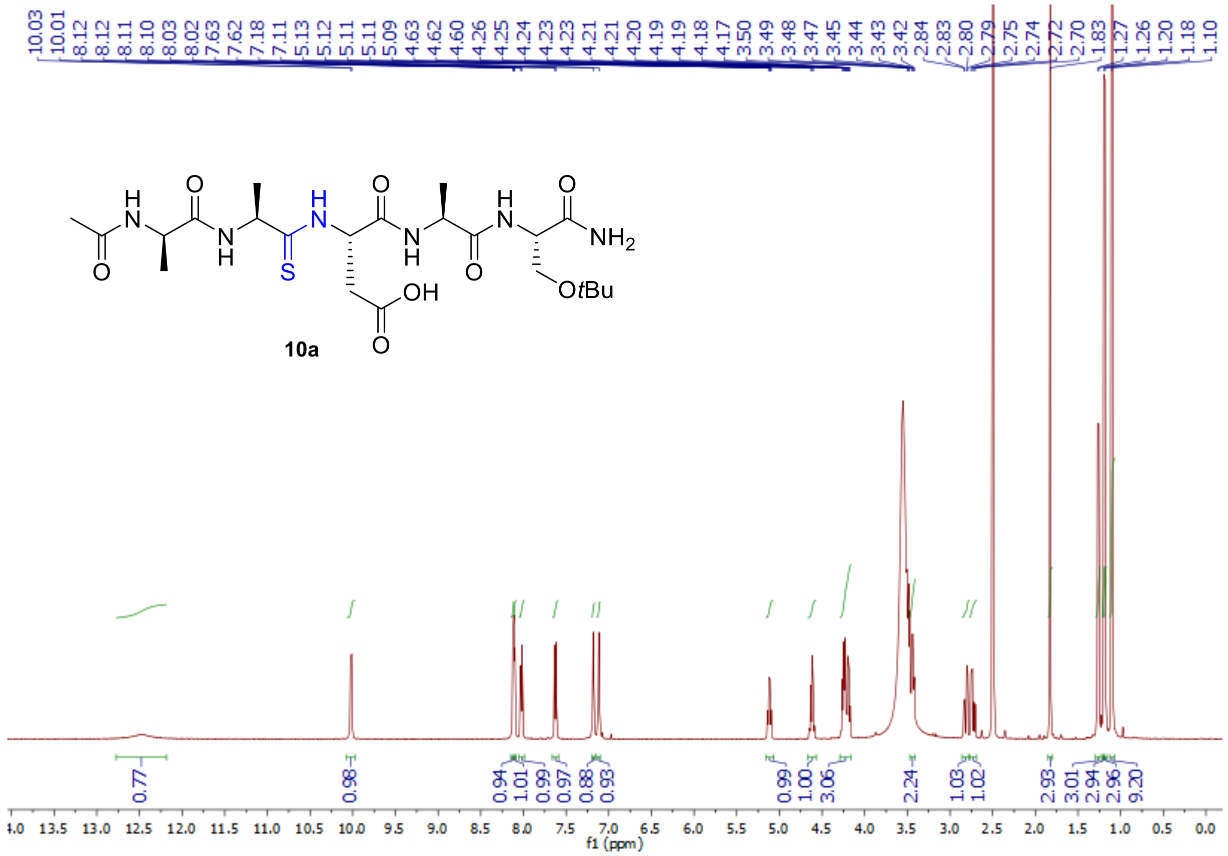

Fig S26: ^{1}H NMR of **10b** (500 MHz, DMSO-d$_6$)

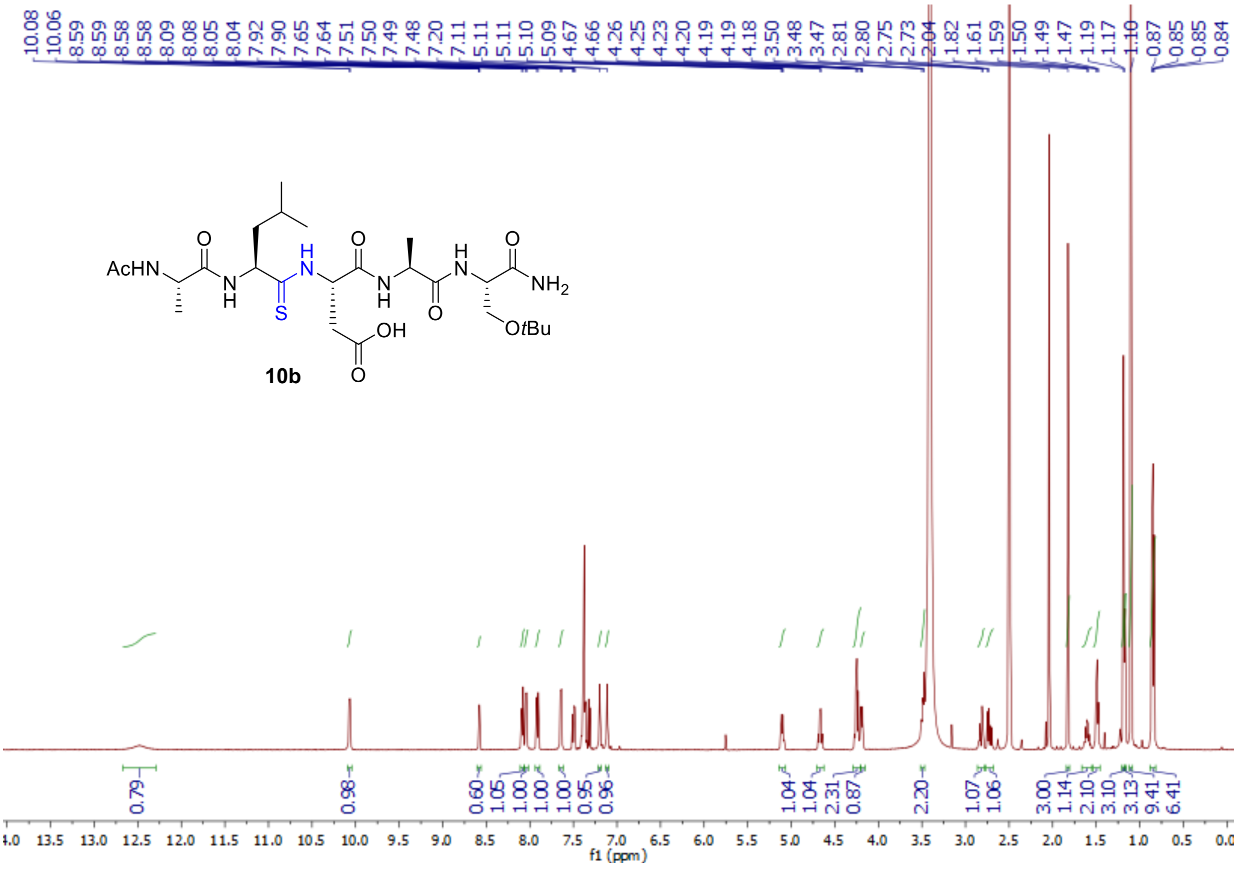

Fig S27: ^{1}H NMR of **10c** (500 MHz, DMSO-d$_6$)

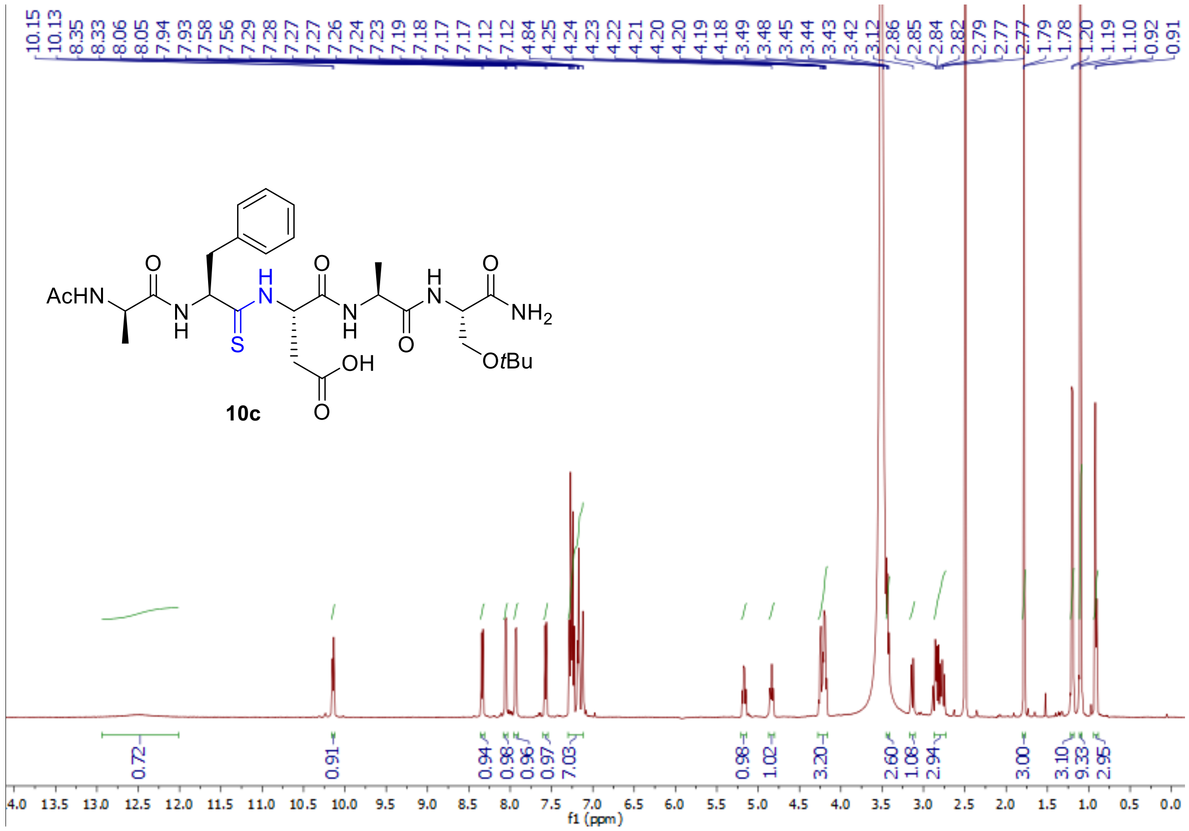

Fig S28: ^{1}H NMR of **10d** (500 MHz, DMSO-d$_6$)

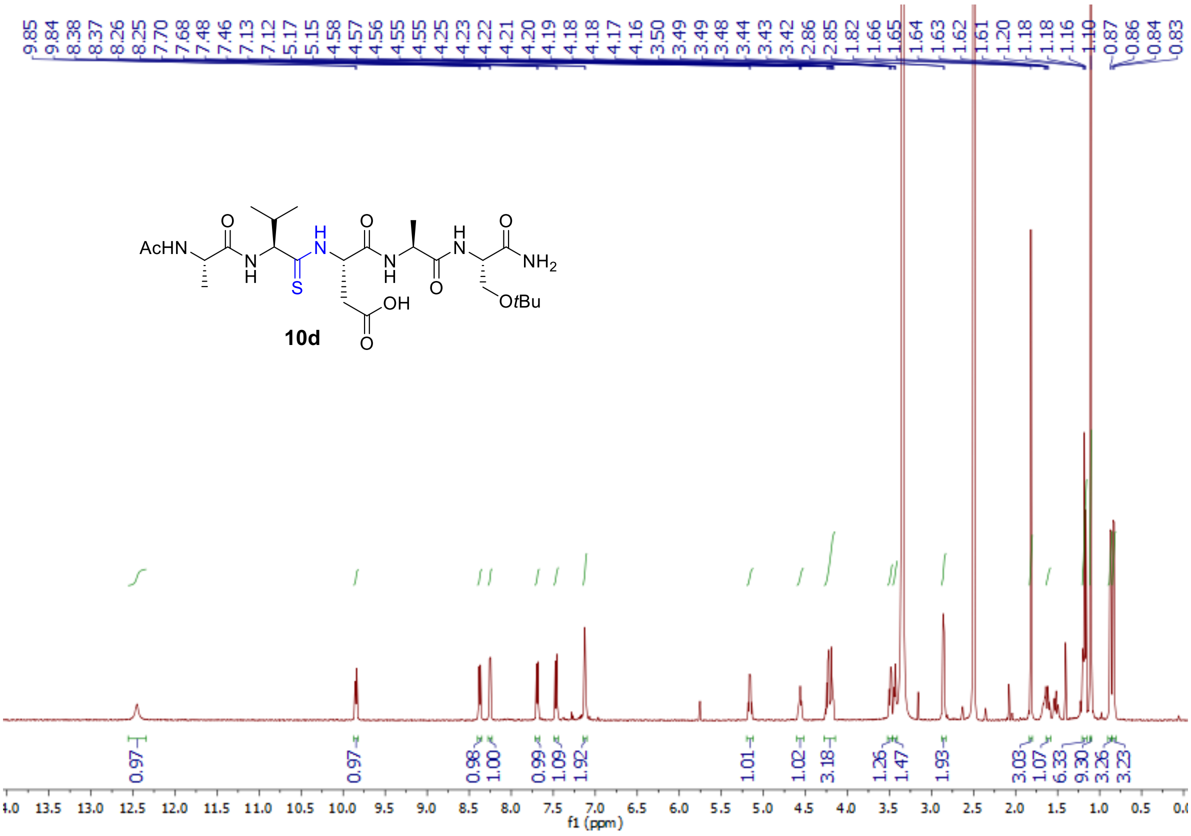

Fig S29: ^{1}H NMR of **10e** (500 MHz, DMSO-d_6)

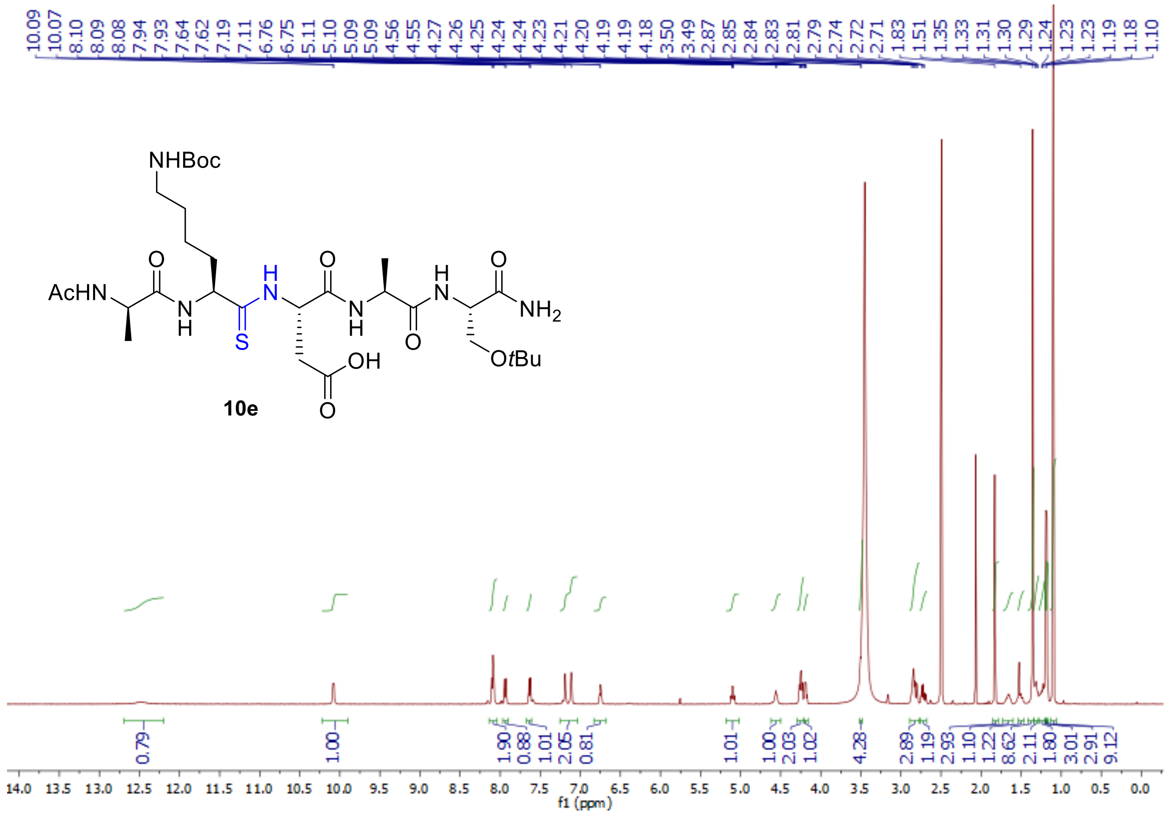

Fig S30: ^{1}H NMR of **10f** (500 MHz, DMSO-d_6)

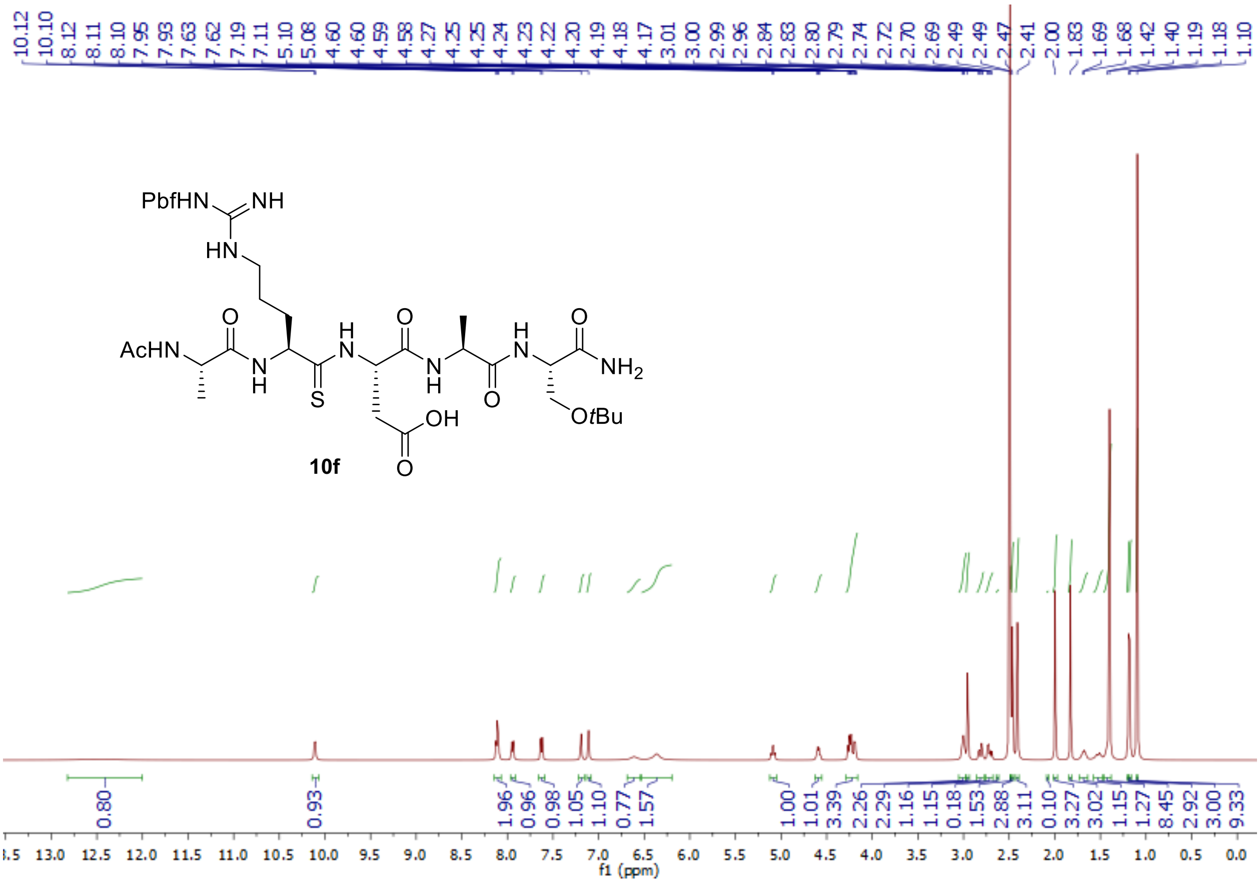

Fig S31: ^{1}H NMR of **11a** (500 MHz, DMSO-d$_6$)

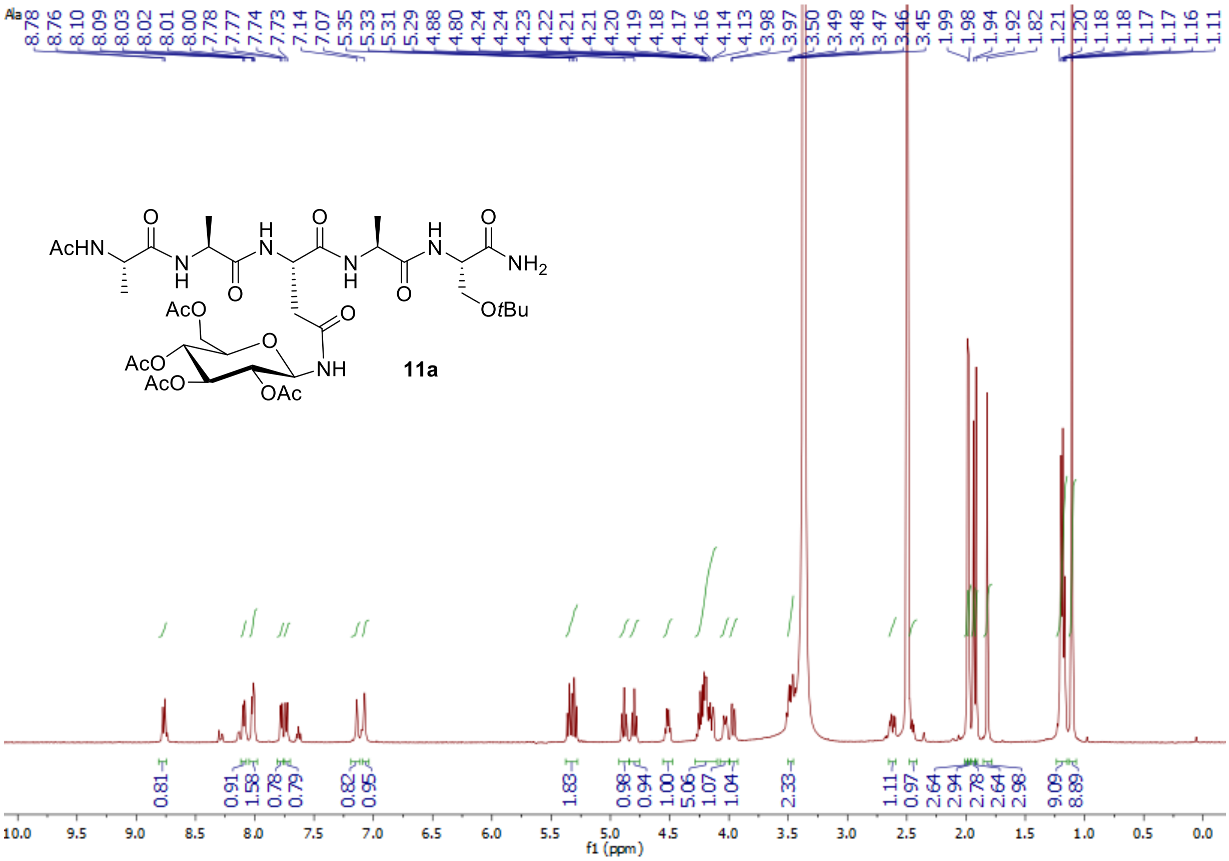

Fig S32: ESI MS of **11a**

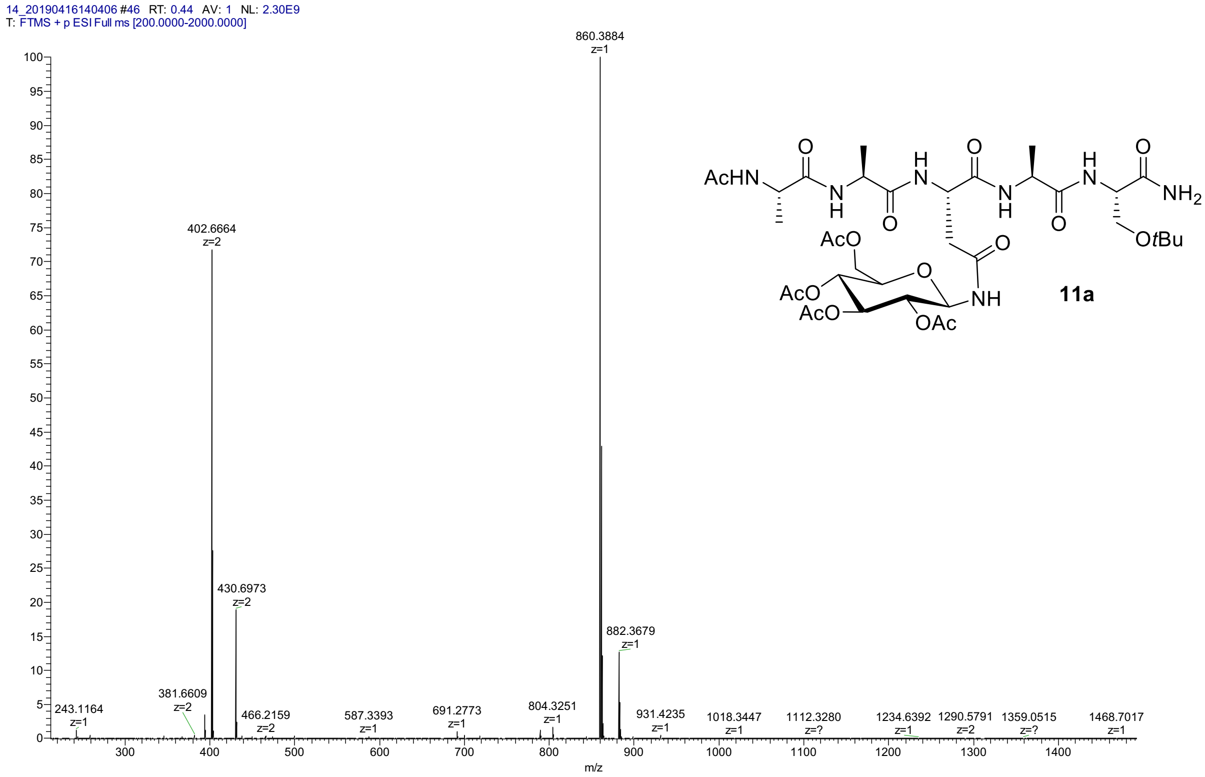

Fig S33: ^{1}H NMR of **11b** (500 MHz, DMSO-d$_6$)

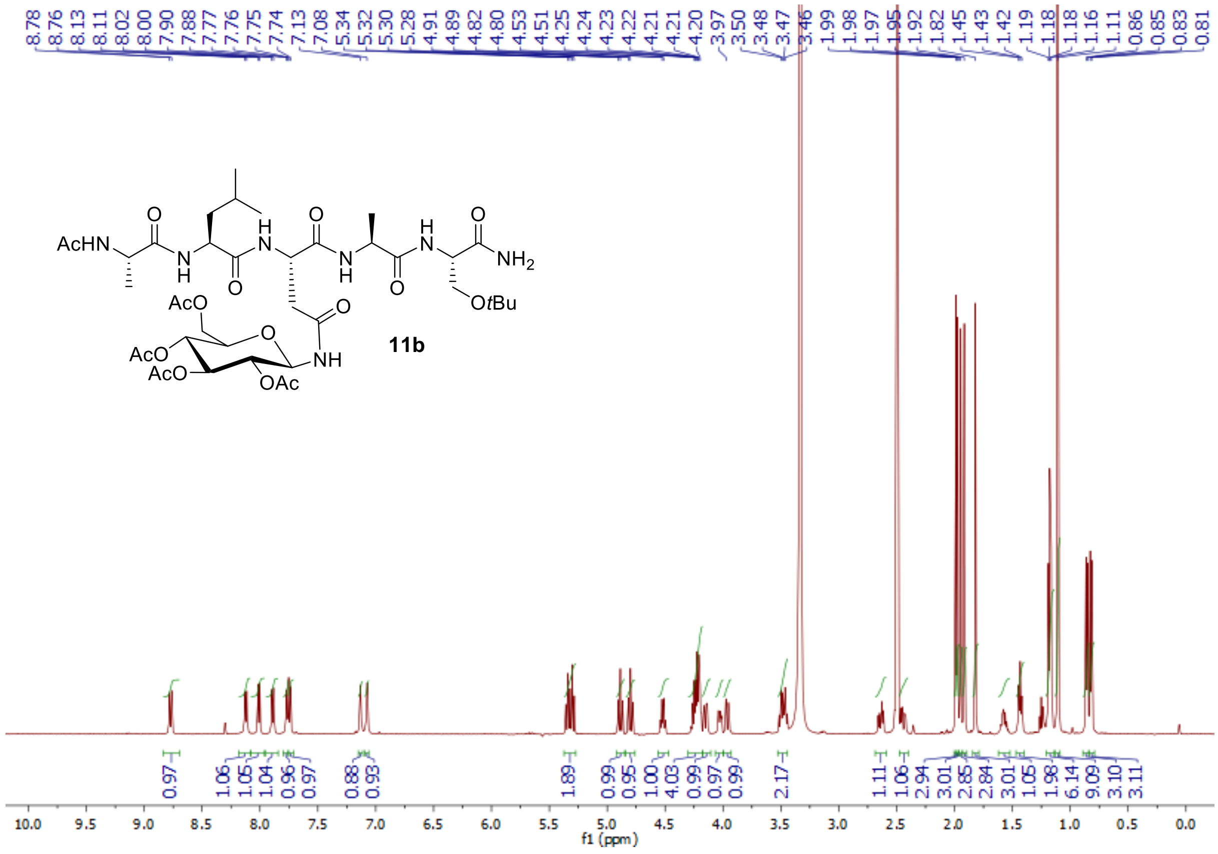

Fig S34: ESI MS of **11b**

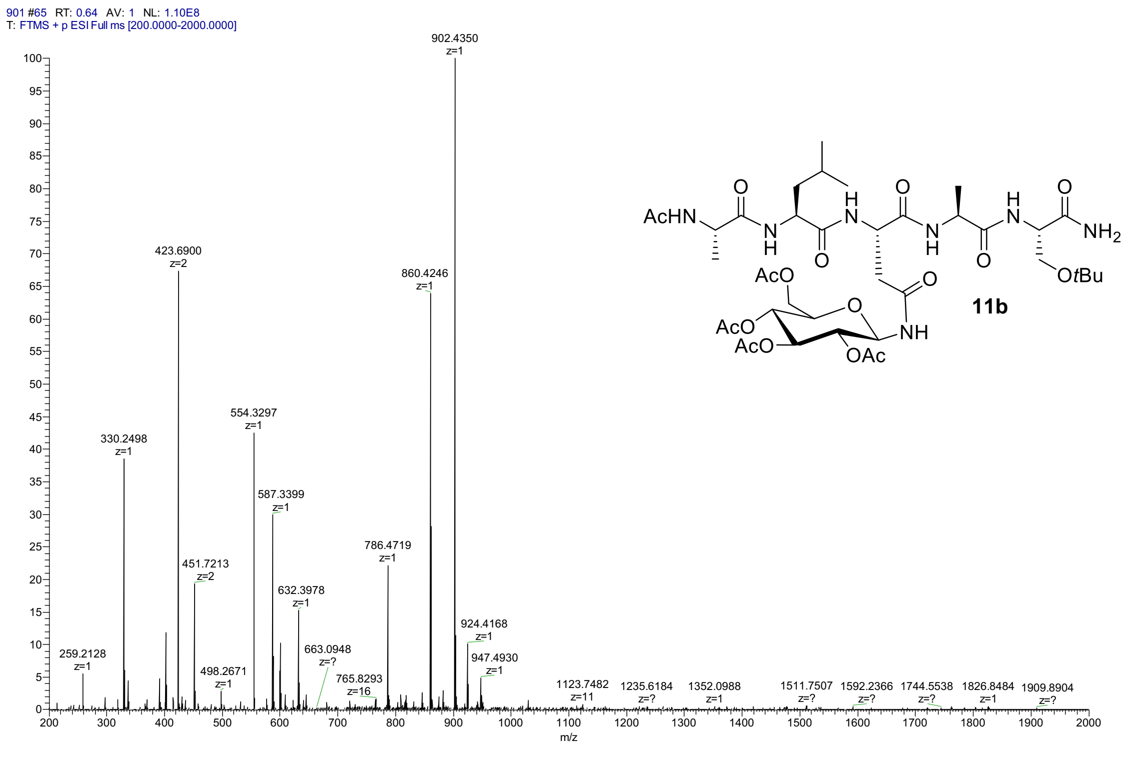

Fig S35: ^{1}H NMR of **11c** (500 MHz, DMSO-d$_6$)

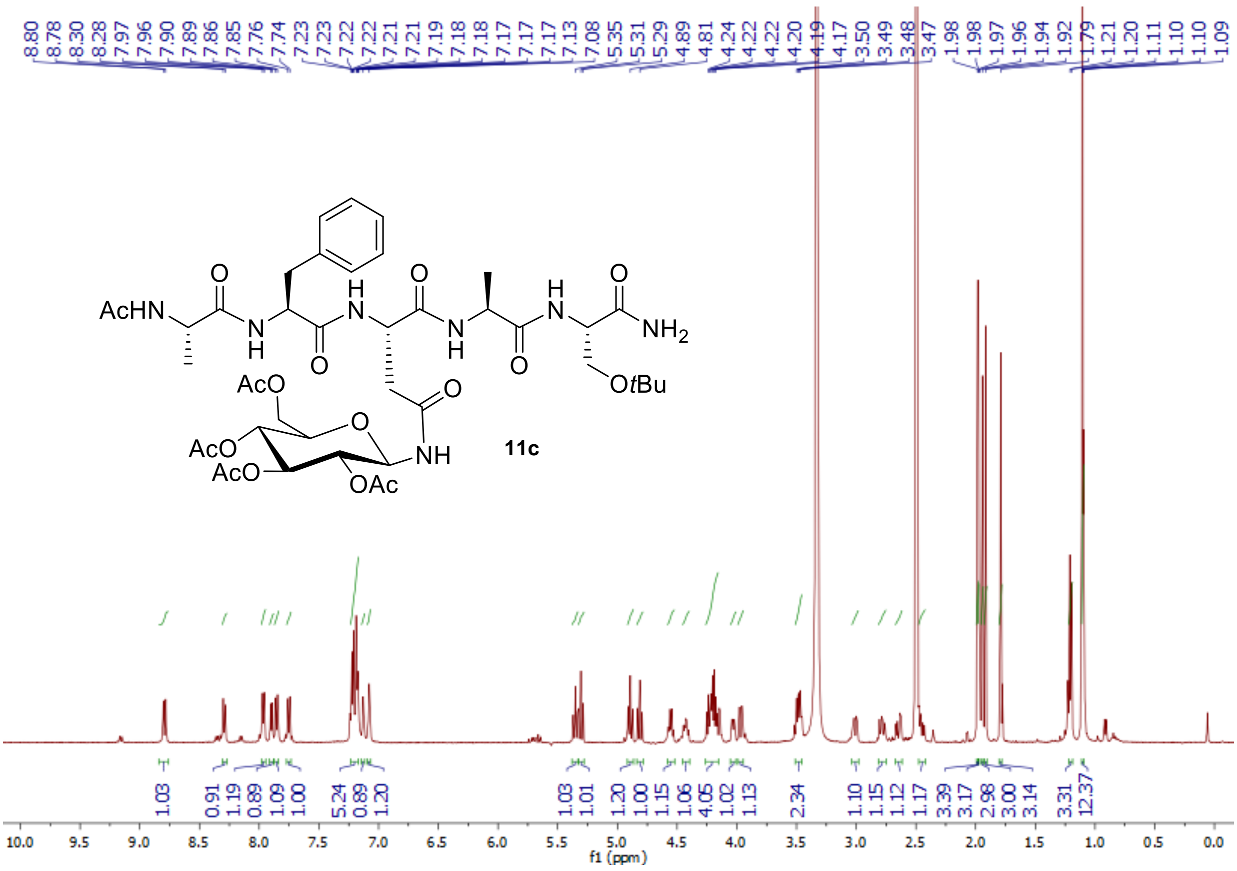

Fig S36: ESI MS of **11c**

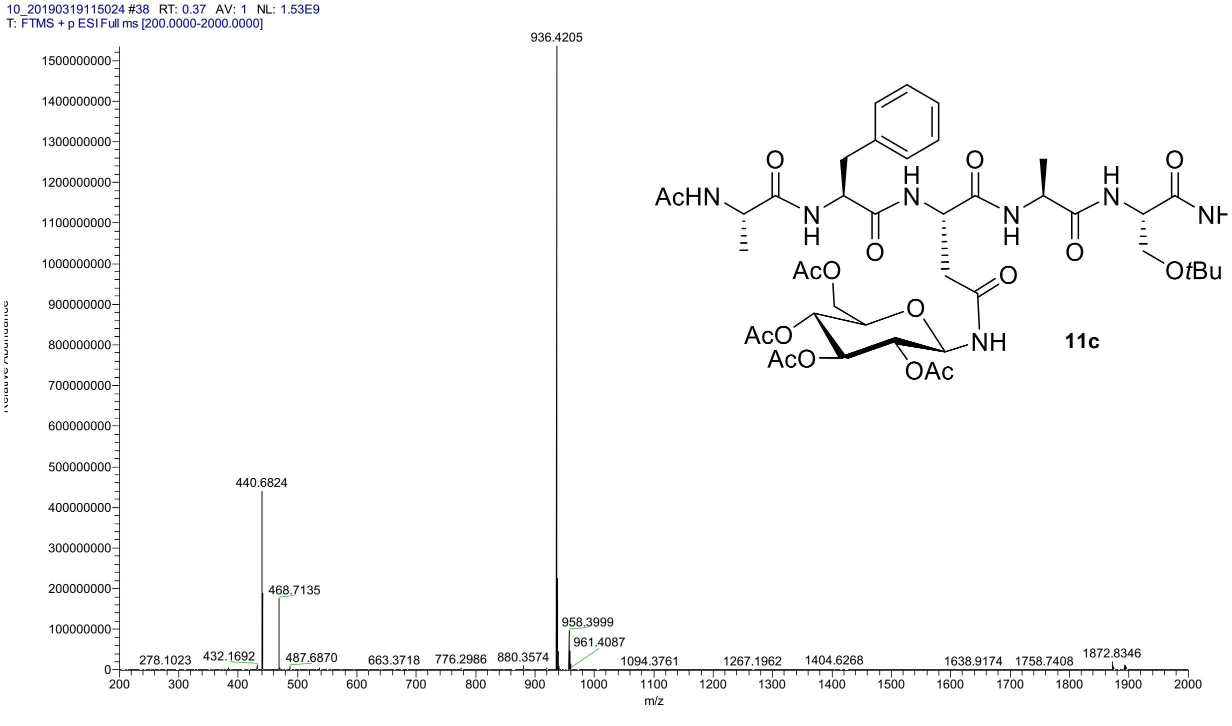

Fig S37: ^{1}H NMR of **11d** (500 MHz, DMSO-d$_6$)

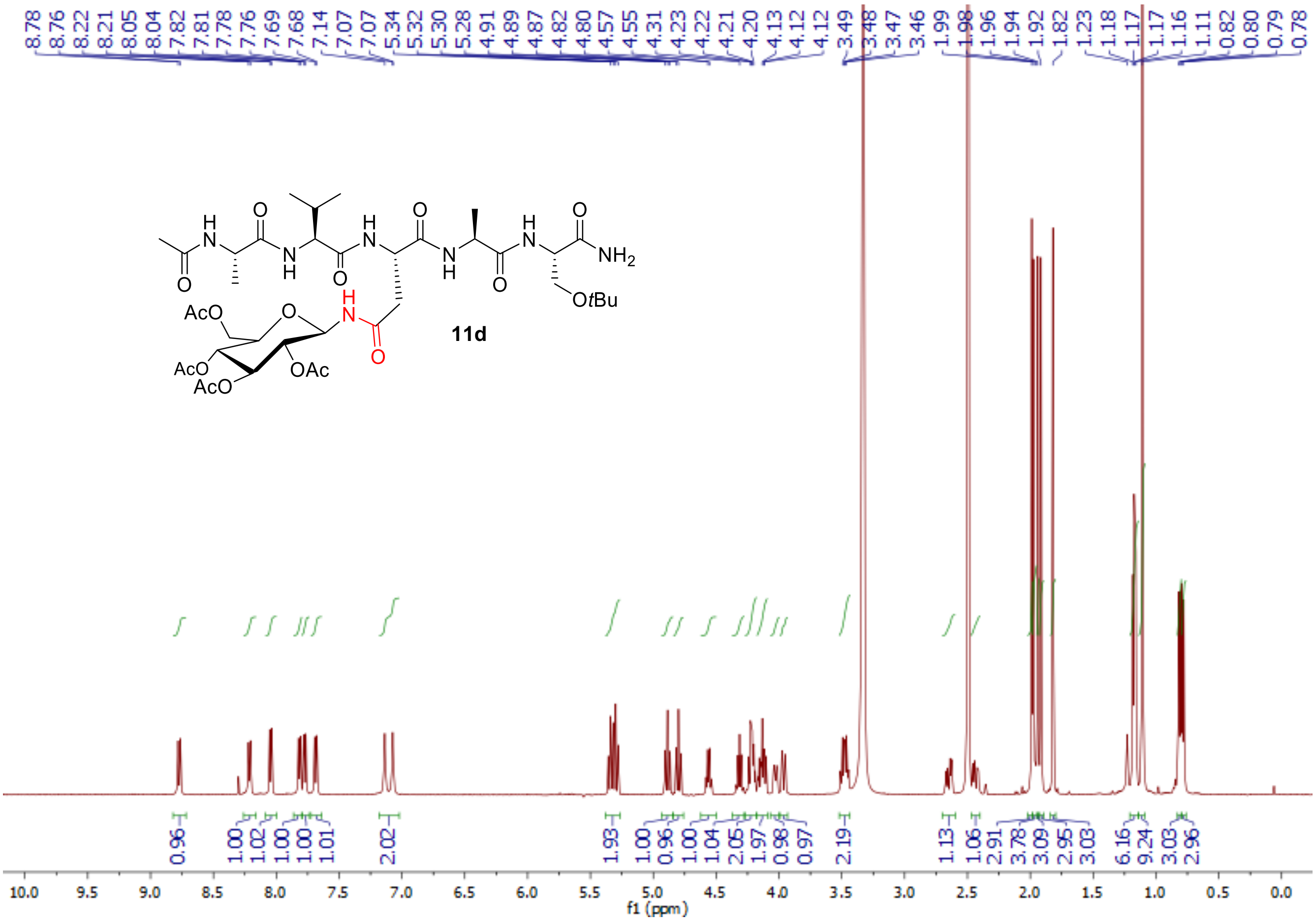

Fig S38: ESI MS of **11d**

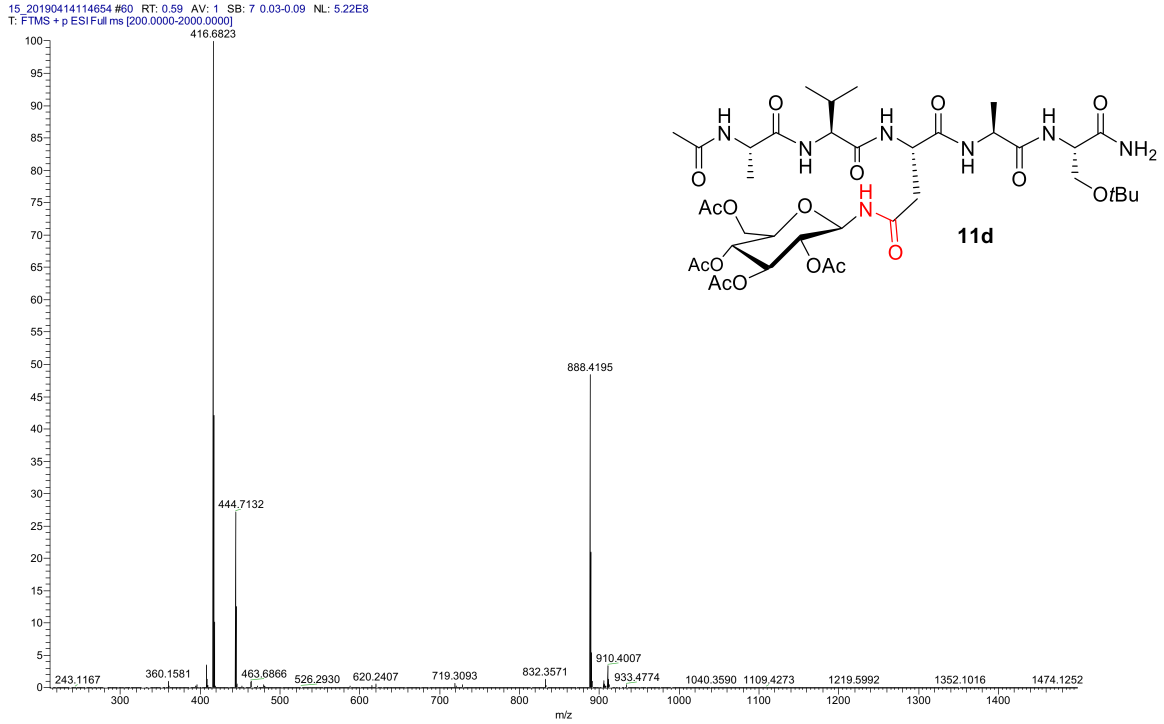

Fig S39: ^{1}H NMR of **11c** (500 MHz, DMSO-d$_6$)

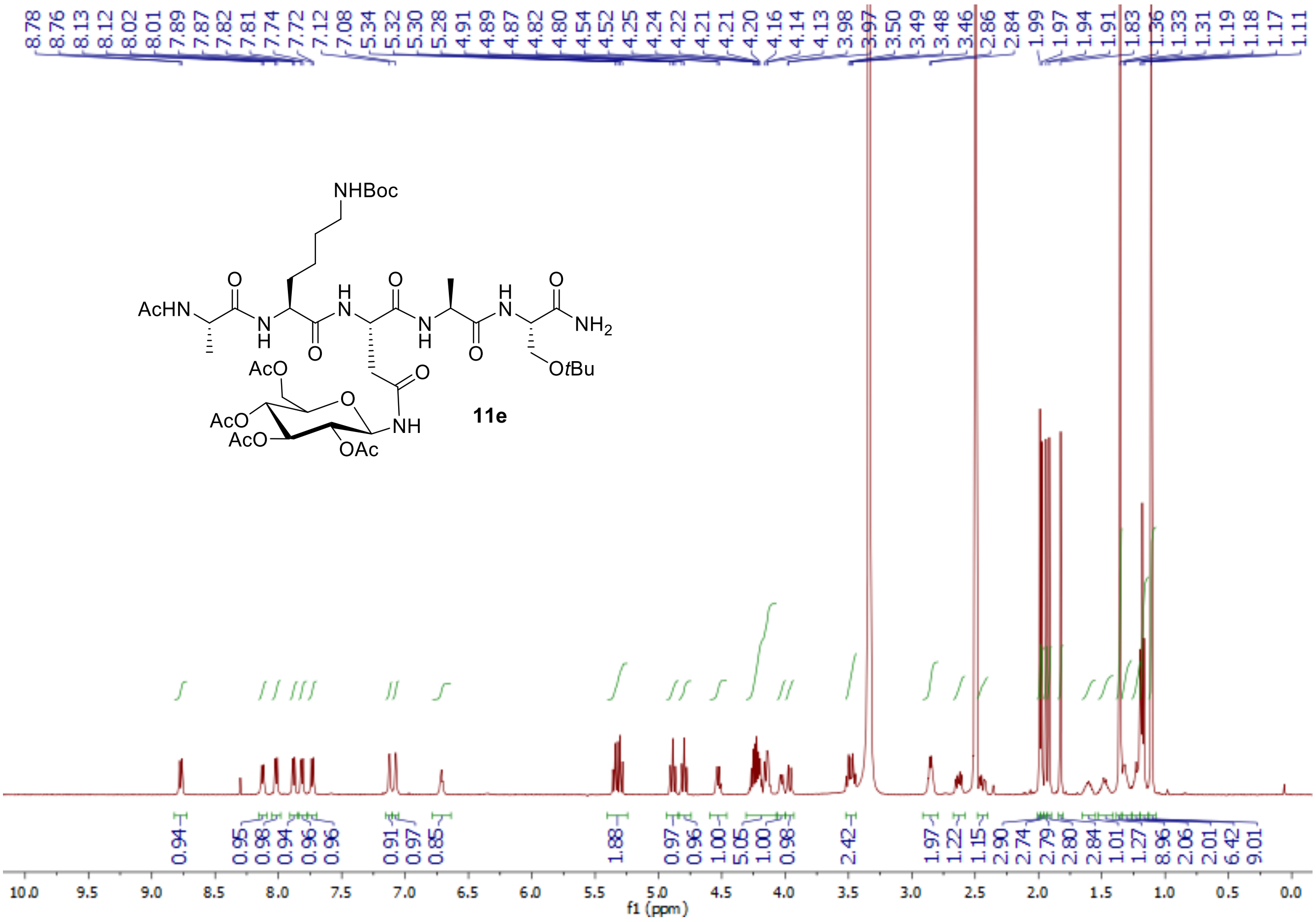

Fig S40: ESI MS of **11e**

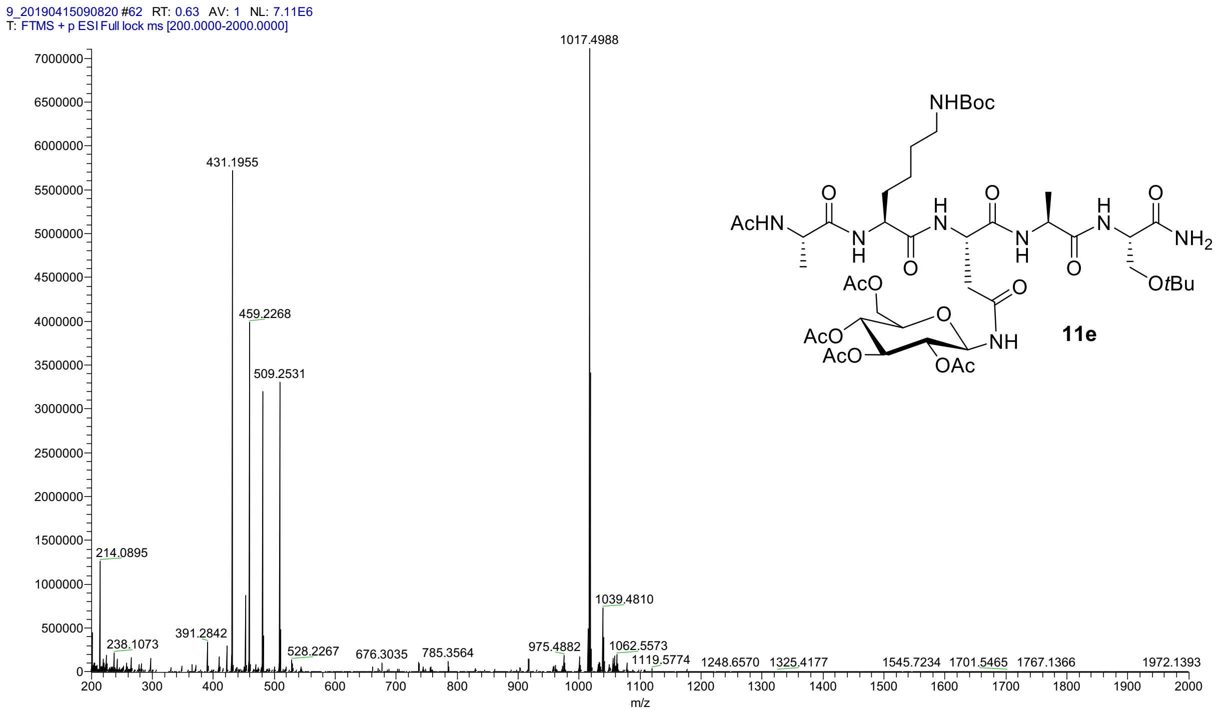

Fig S41: ^{1}H NMR of **11f** (500 MHz, DMSO-d_6)

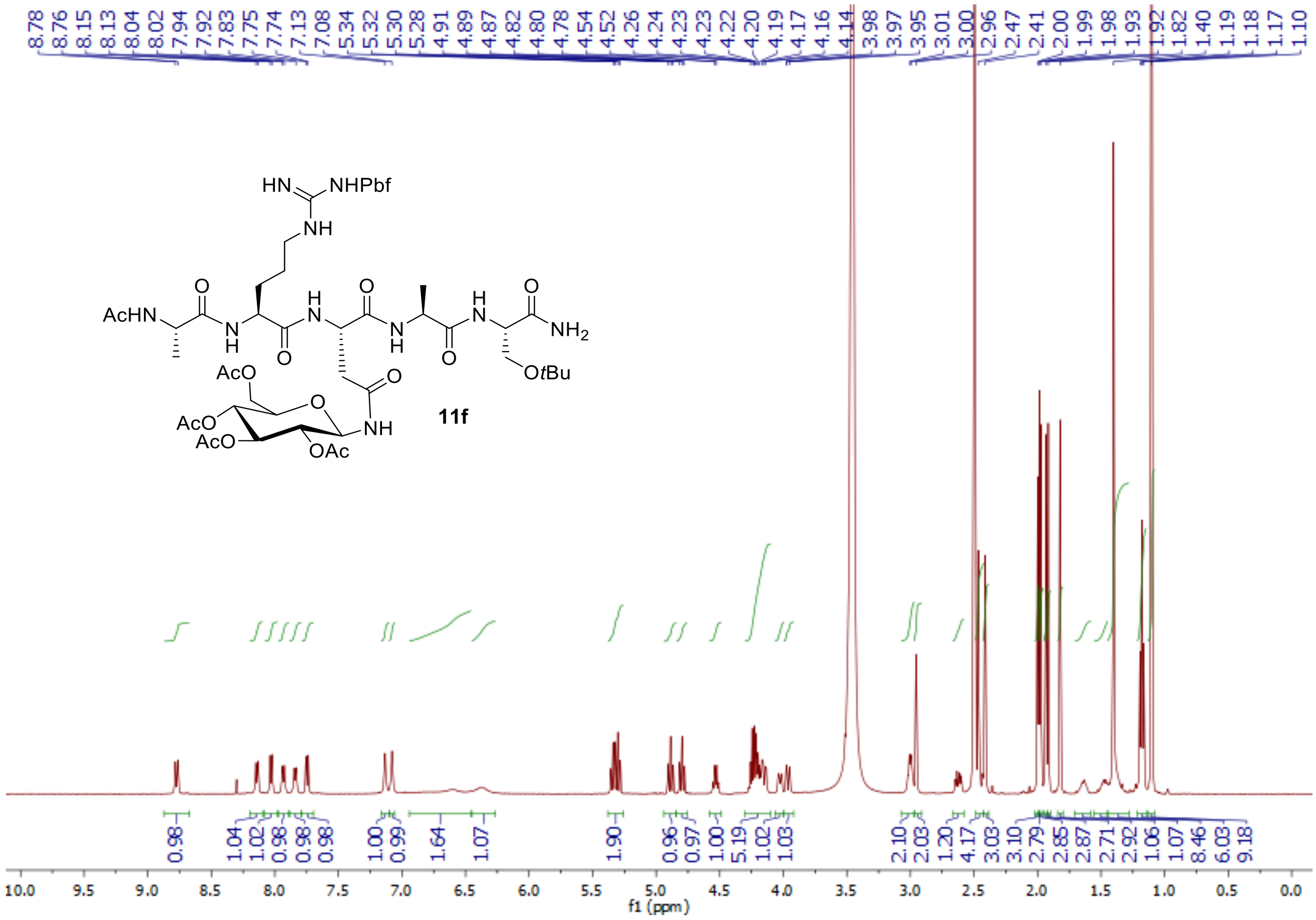

Fig S42: ESI MS of **11f**

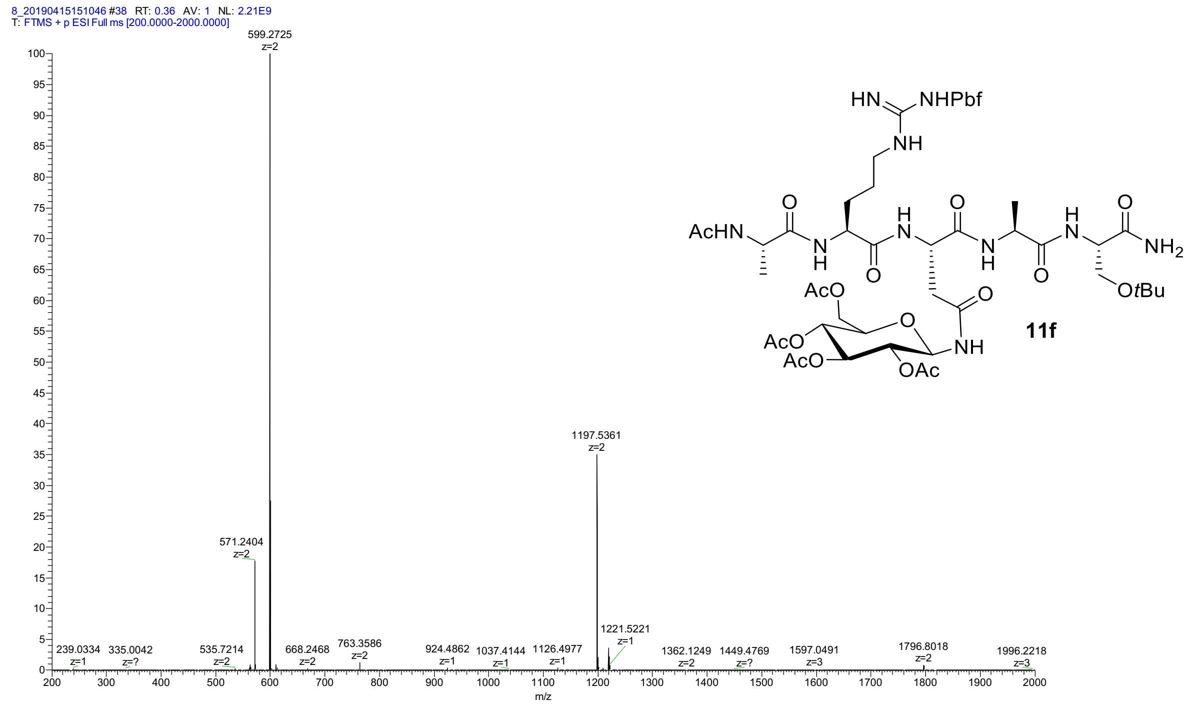

Fig S43: ^{1}H NMR of **13** (500 MHz, DMSO-d$_6$)

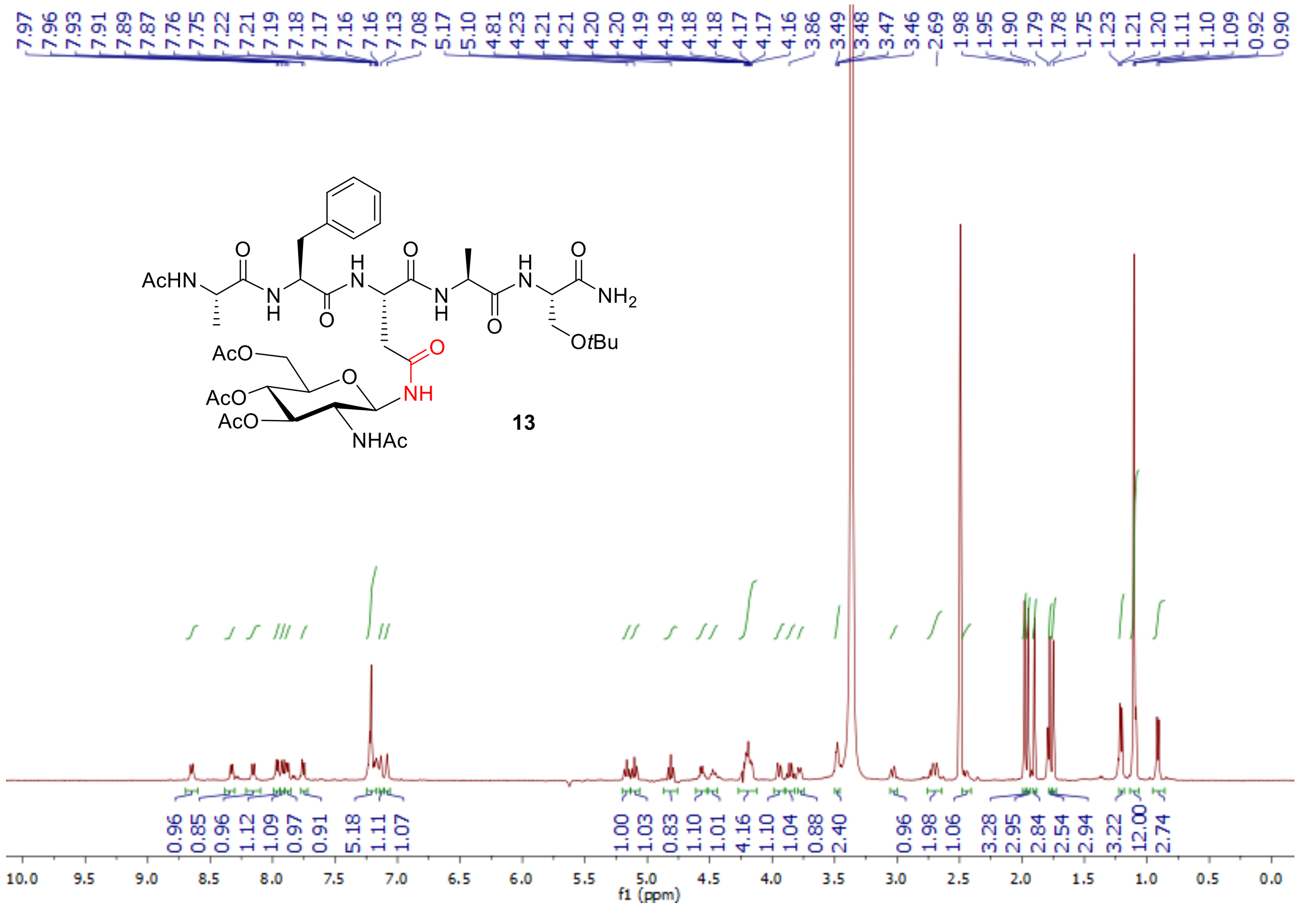

Fig S43: ESI MS of **13**

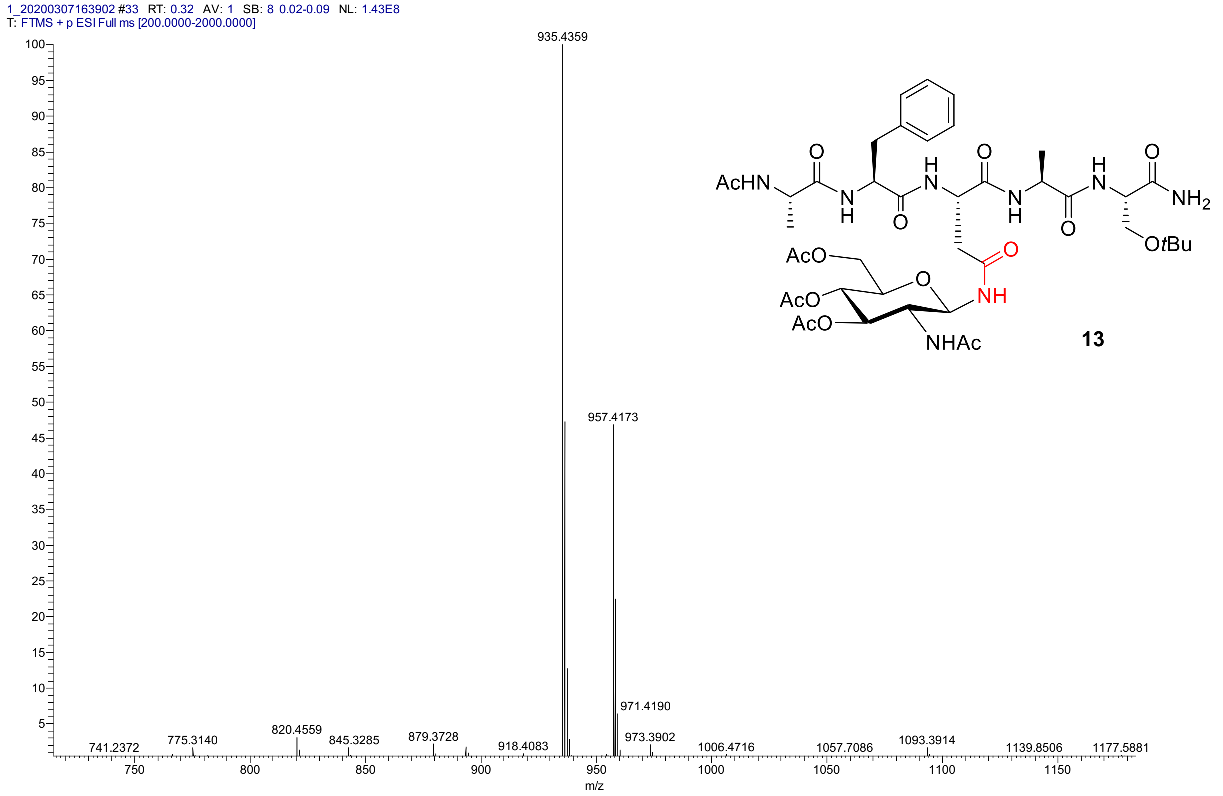

Fig S45: HPLC trace of purified peptide thioamide **14a**

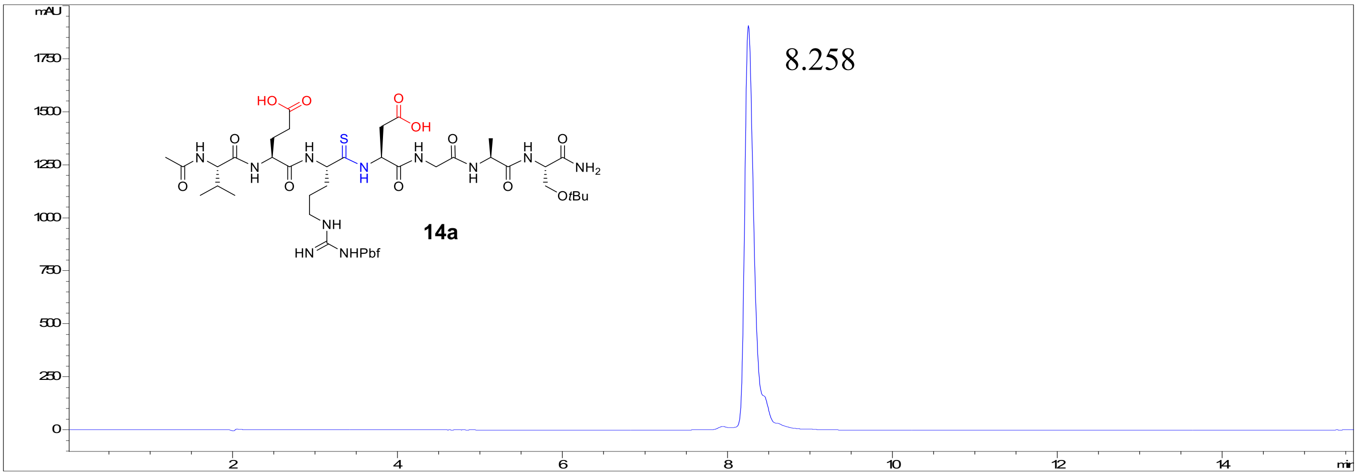

Fig S45: ESI MS of purified peptide thioamide **14a**

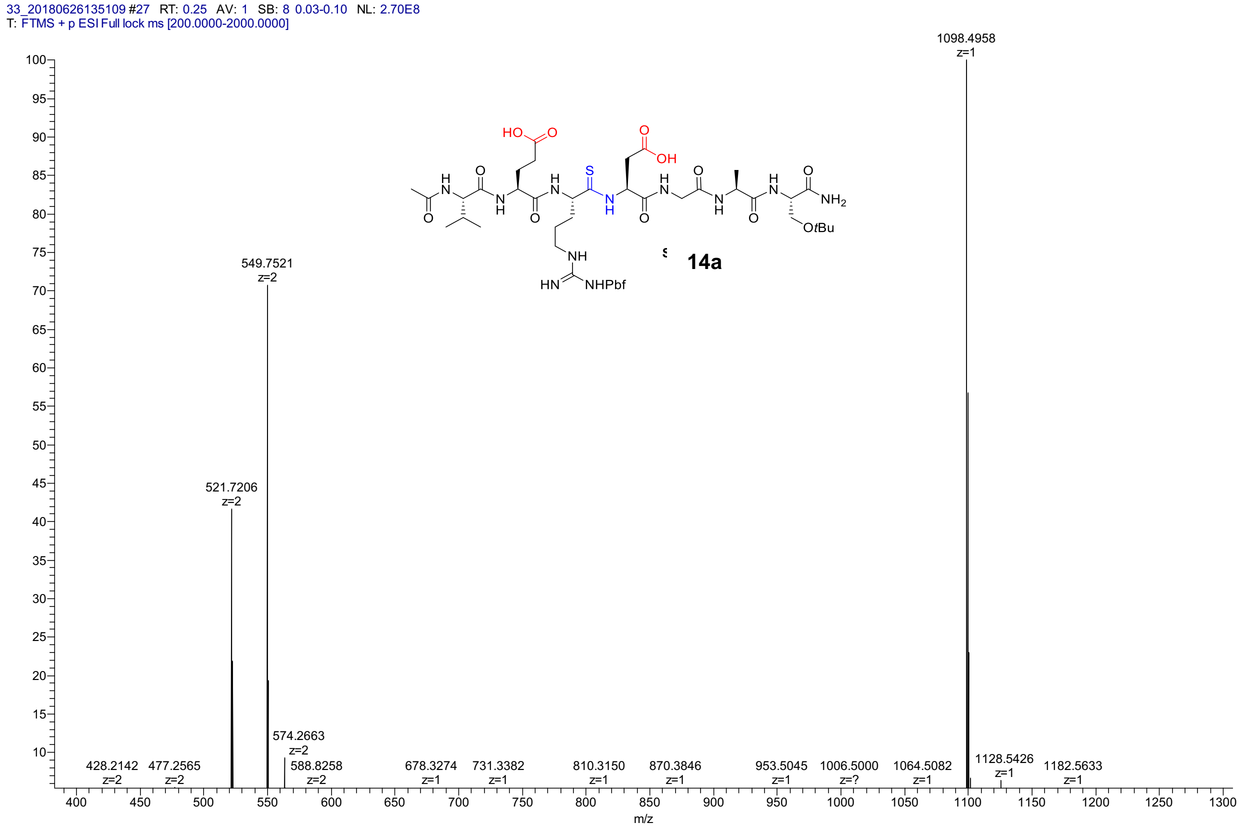

Fig S46: HPLC trace of crude glycopeptide **15a**

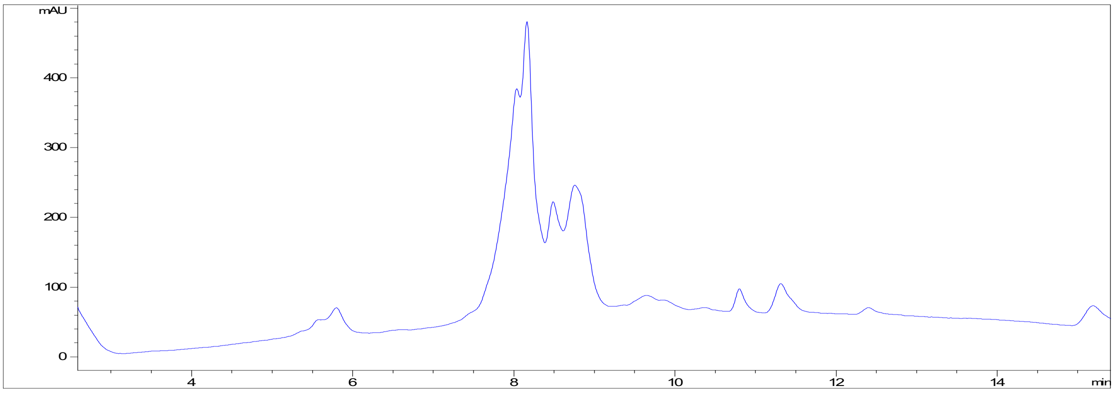

Fig S47: HPLC trace of purified glycopeptide **15a**

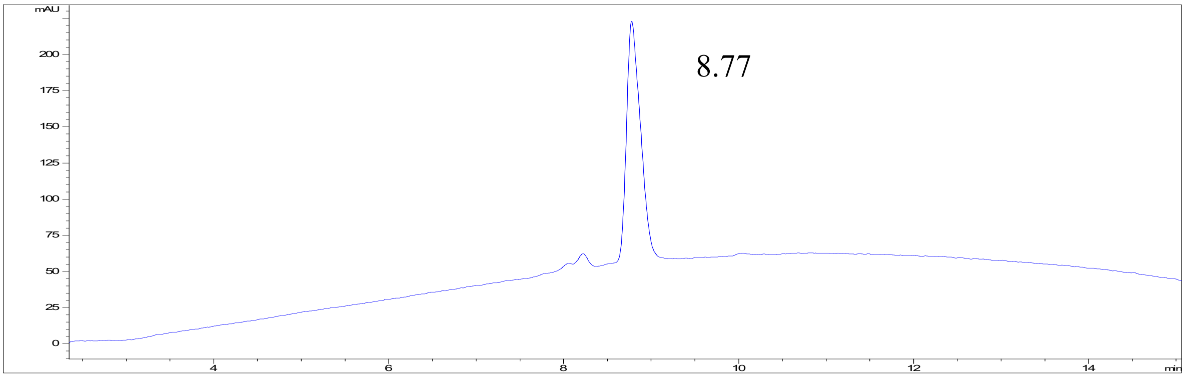

Fig S48: ESI MS of purified glycopeptide **15a**

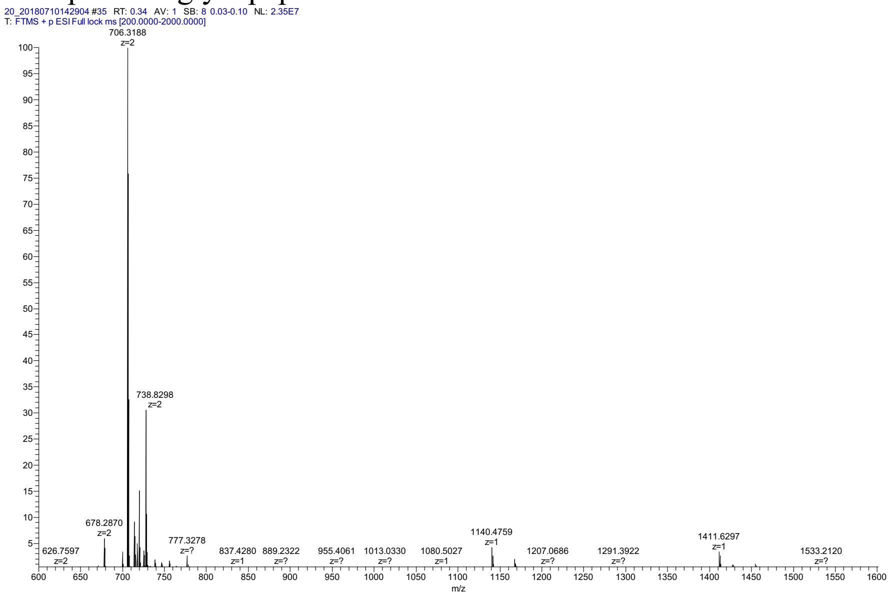

Fig S49: HPLC trace of purified peptide thioamide **14b**

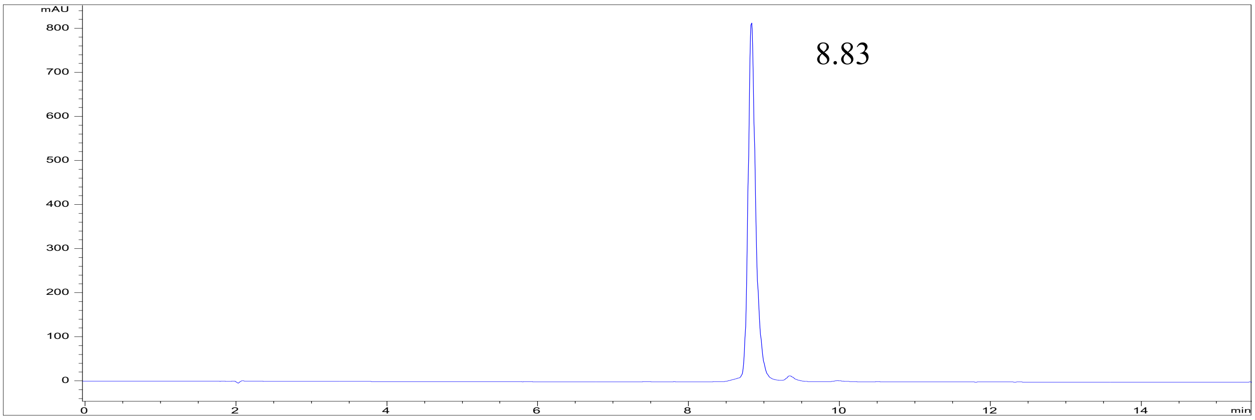

Fig S50: ESI MS of purified peptide thioamide **14b**

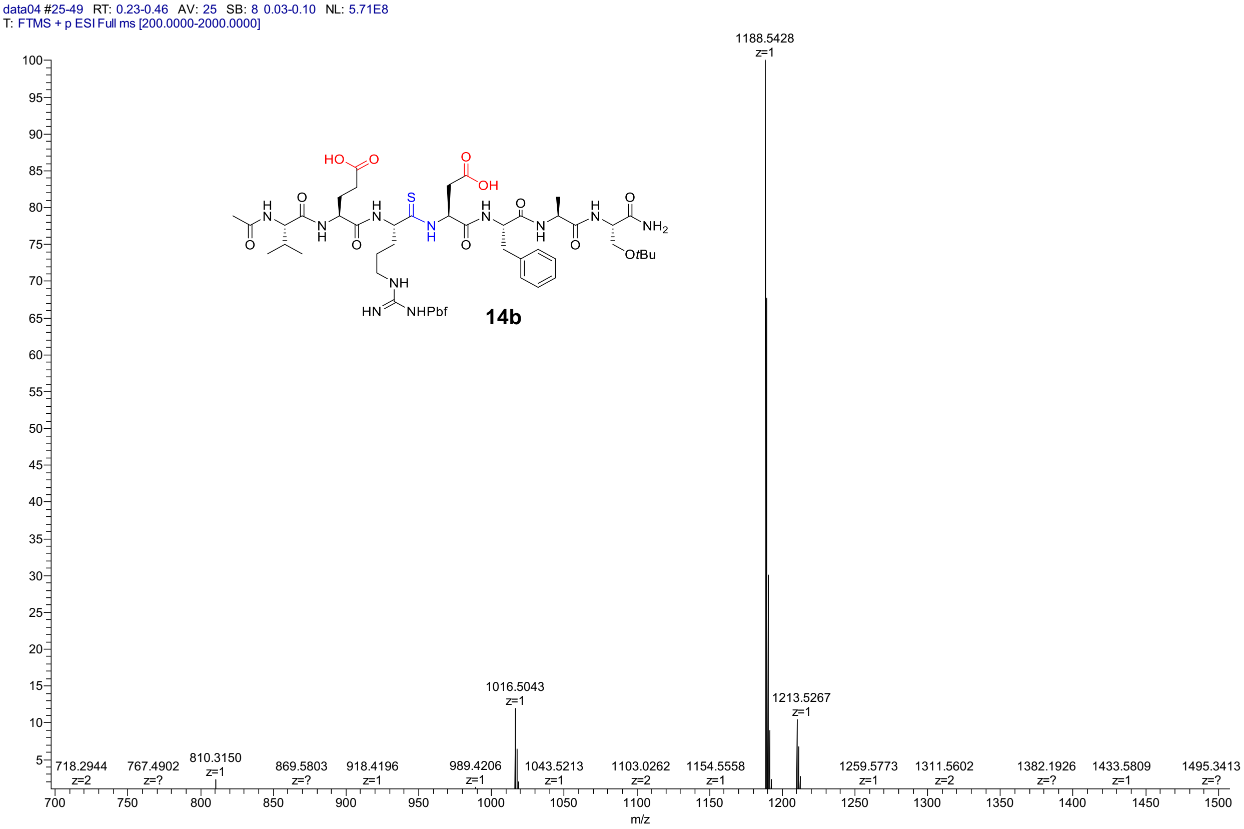

Fig S51: HPLC trace of crude peptide thioamide **15b**

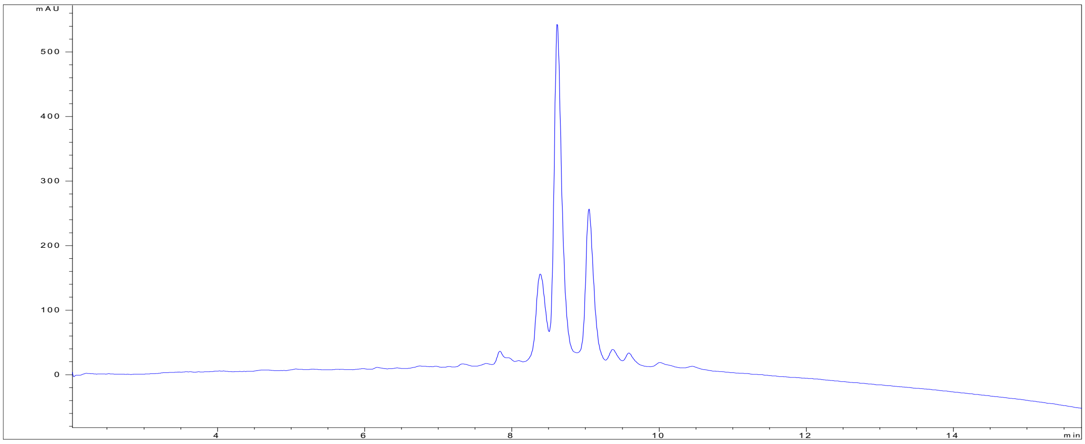

Fig S52: HPLC trace of purified peptide thioamide **15b**

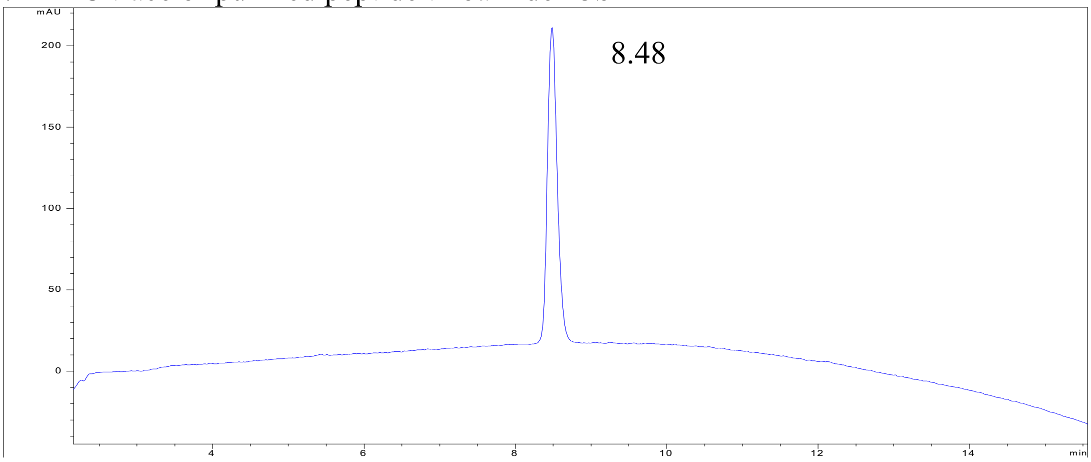

Fig S53: ESI MS of purified peptide thioamide **15b**

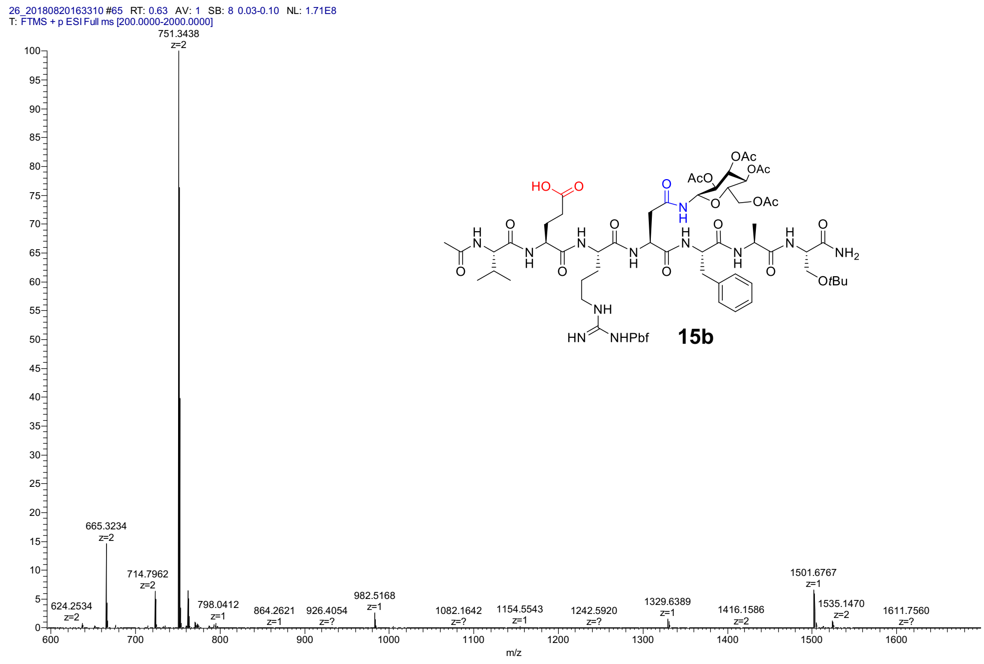

Fig S54: HPLC trace of purified peptide thioamide **14d**

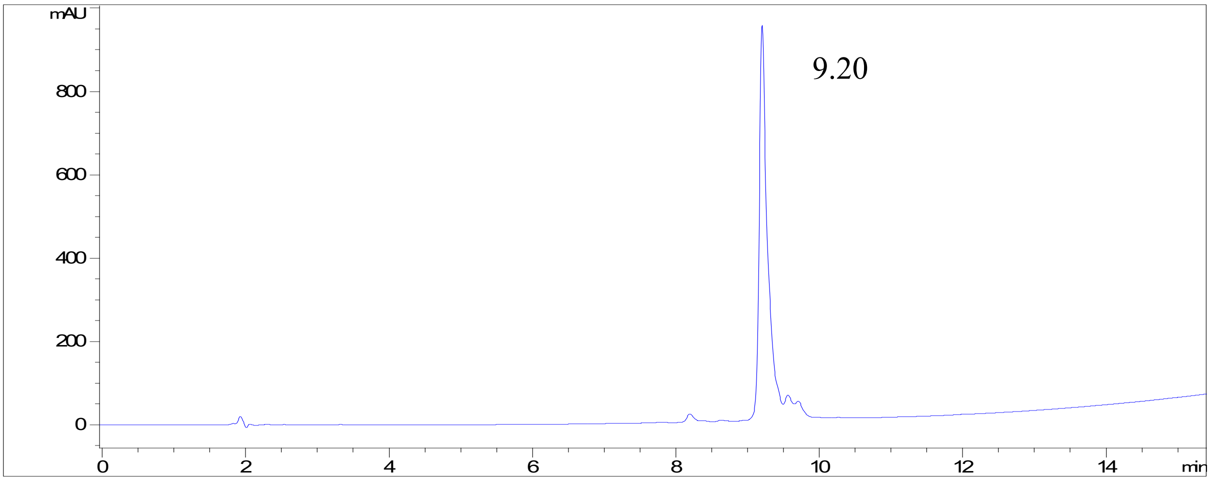

Fig S55: ESI MS of purified peptide thioamide **14d**

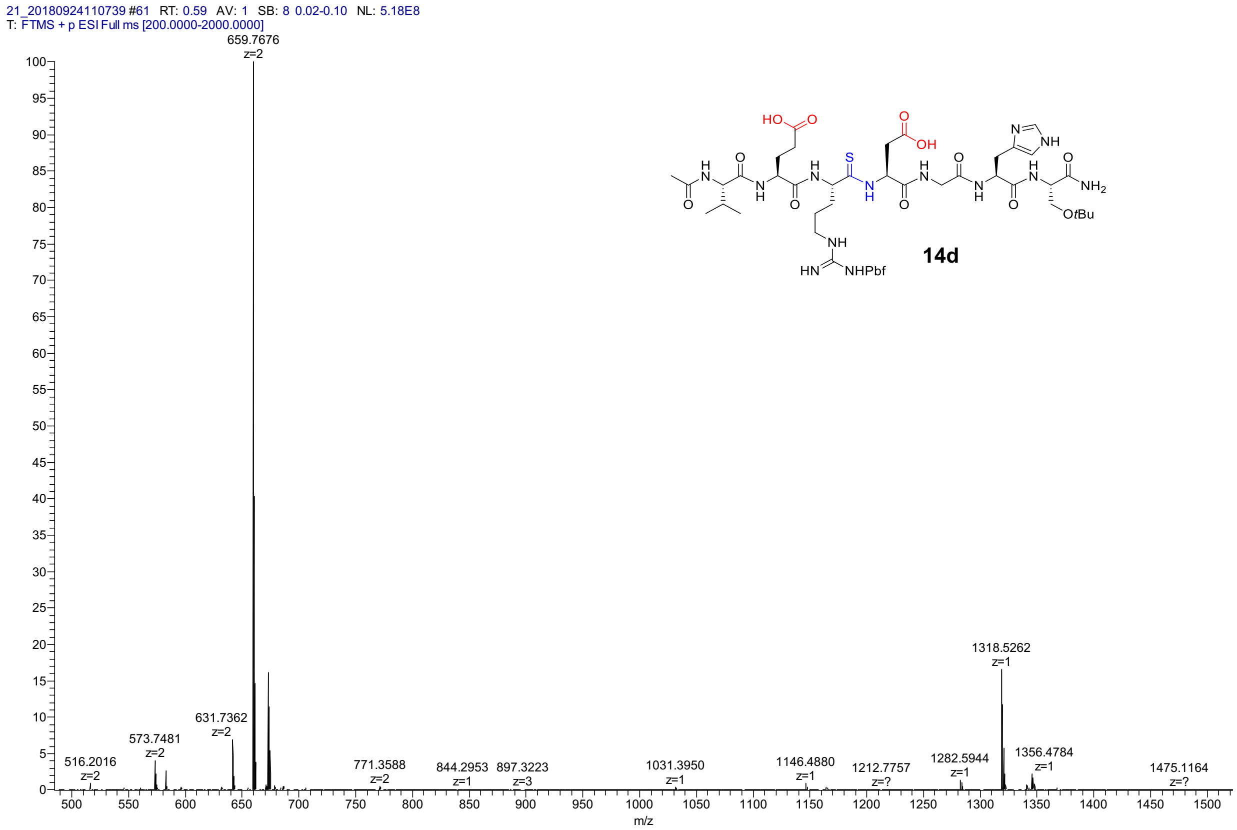

Fig S56: HPLC trace of purified peptide thioamide **15d**

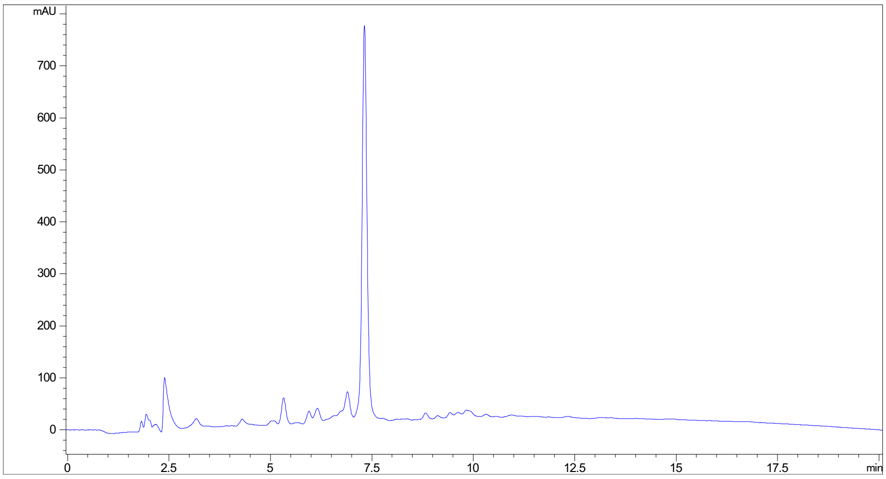

Fig S57: HPLC trace of purified peptide thioamide **15d**

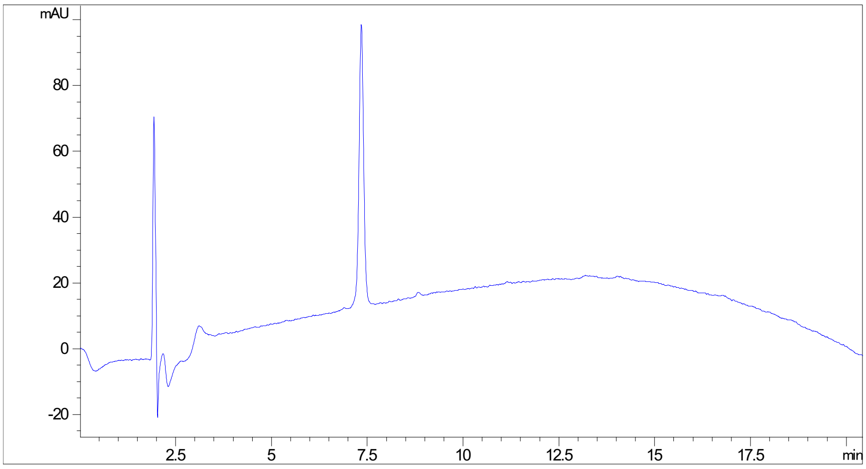

Fig S58: ESI MS of purified peptide thioamide **15d**

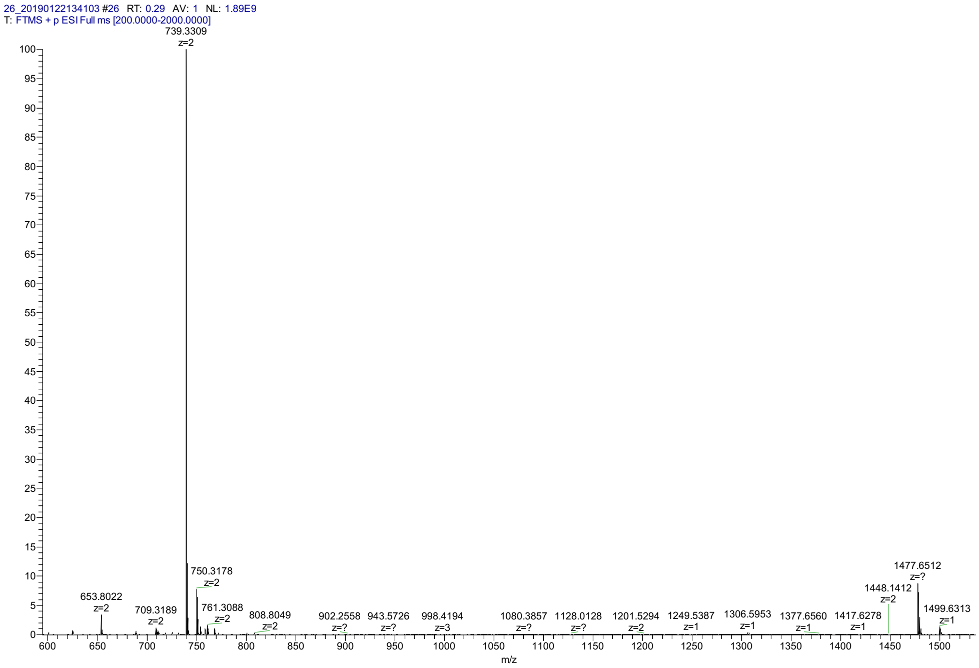

References:

1. Mukherjee, S.; Verma, H.; Chatterjee, J. *Org. Lett.* **2015**, *17*, 3150–3153.
2. Mukherjee, S.; Chatterjee, J. *J. Pept. Sci.* **2016**, *22*, 664–672.
3. Murphy, P. V.; Bradley, H.; Tosin, M.; Pitt, N.; Fitzpatrick, G. M.; Glass, W. K., *J. Org. Chem.* **2003**, *68*, 5692-5704.
